# Structure-Based Drug Design of ADRA2A Antagonists Derived from Yohimbine

**DOI:** 10.1021/acs.jmedchem.4c00323

**Published:** 2024-06-10

**Authors:** Artem Chayka, Michal Česnek, Erika Kužmová, Jaroslav Kozák, Eva Tloušt'ová, Alexandra Dvořáková, Timotej Strmeň, Břetislav Brož, Zuzana Osifová, Martin Dračínský, Helena Mertlíková-Kaiserová, Zlatko Janeba

**Affiliations:** Institute of Organic Chemistry and Biochemistry of the Czech Academy of Sciences, Flemingovo nám. 2, Prague 6 160 00, Czech Republic

## Abstract

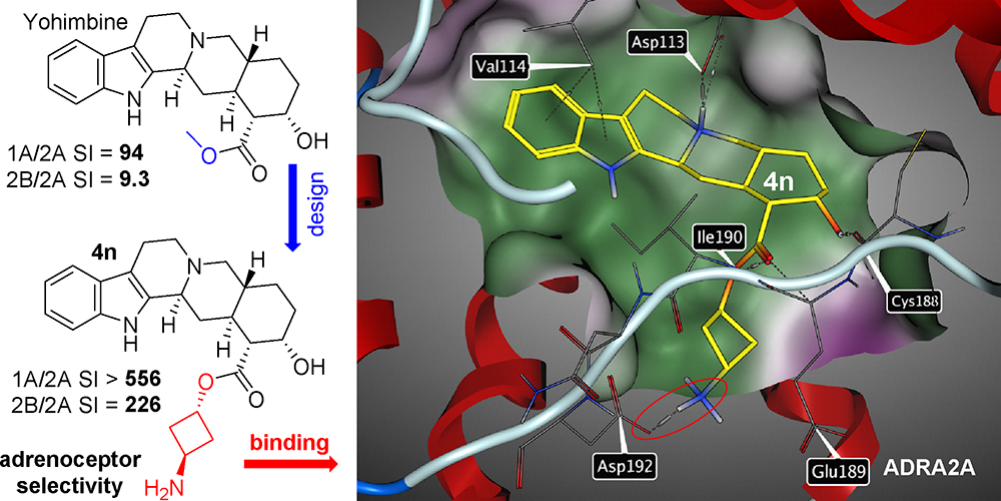

Yohimbine, a natural indole alkaloid and a nonselective adrenoceptor antagonist, possesses potential benefits in treating inflammatory disorders and sepsis. Nevertheless, its broader clinical use faces challenges due to its low receptor selectivity. A structure–activity relationship study of novel yohimbine analogues identified amino esters of yohimbic acid as potent and selective ADRA2A antagonists. Specifically, amino ester **4n**, in comparison to yohimbine, showed a 6-fold higher ADRA1A/ADRA2A selectivity index (SI > 556 for **4n**) and a 25-fold higher ADRA2B/ADRA2A selectivity index. Compound **4n** also demonstrated high plasma and microsomal stability, moderate-to-low membrane permeability determining its limited ability to cross the blood–brain barrier, and negligible toxicity on nontumor normal human dermal fibroblasts. Compound **4n** represents an important complementary pharmacological tool to study the involvement of adrenoceptor subtypes in pathophysiologic conditions such as inflammation and sepsis and a novel candidate for further preclinical development to treat ADRA2A-mediated pathologies.

## 1 Introduction

Around 11 million sepsis-related deaths are reported every year, which make acute sepsis and septic shock one of the leading causes of death in intensive care units around the world.^1^ Septic shock is characterized by multiple organ failure, which is generally initiated by liver inflammation.^2−4^ An extensive release of proinflammatory cytokines, such as IL-6, IL-1β, IL-10, and tumor necrosis factor-alpha (TNF-alpha), results from an activation of alpha-2A adrenergic receptor (α_2A_ adrenoceptor, ADRA2A) in Kupffer cells.^5,6^ These receptors are activated by noradrenaline (norepinephrine), which is released excessively in the gut during sepsis.^6,7^ Importantly, it has been shown that intraportal infusion of noradrenaline to rats led to increased production of the above-mentioned cytokines, and this effect was reversed upon coinfusion of ADRA2 antagonists yohimbine (nonselective) or BRL-44408 maleate (ADRA2A selective).^6,8^ Furthermore, yohimbine demonstrated significant anti-inflammatory and antifibrotic activity in both *in vitro* (hepatic endothelial and stellate cells and hepatocytes) and *in vivo* (hepatic inflammation/fibrosis) models.^9^

**Figure 1:**
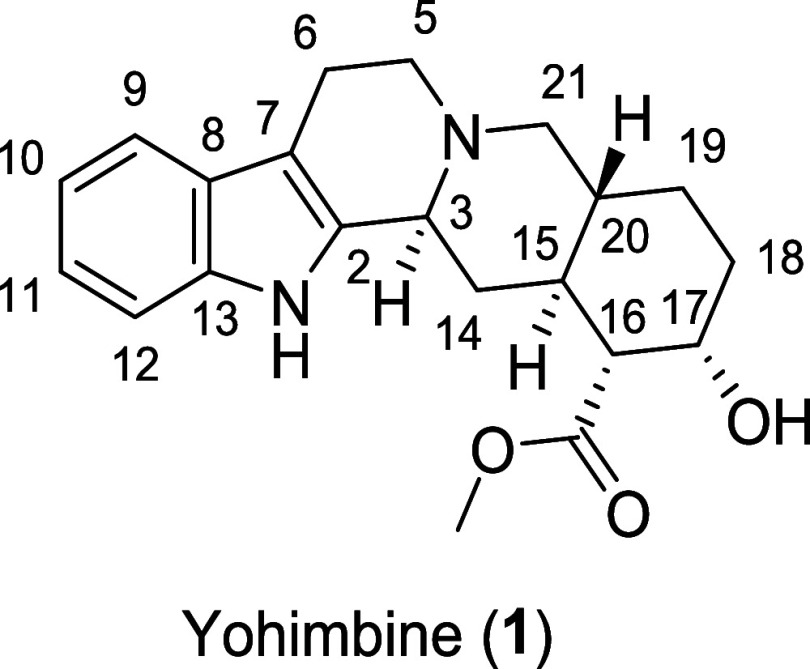
Structure of yohimbine (**1**).

Yohimbine (**1**, Figure 1) is a naturally occurring indole alkaloid found in several plants.^10,11^ Yohimbine can be beneficial in erectile dysfunction, myocardial dysfunction, and inflammatory disorders, and it also has antidiuretic, mydriatic, and serotonin antagonistic properties.^12,13^ Yohimbine exhibits affinity to several types of receptors, namely, ADRA2 > 5-HT_1D_ > 5-HT_1A_ > ADRA1 > D2 > D3, among which the highest affinity is toward ADRA2 receptors (ADRA2A pKi = 8.2–8.5; ADRA2B pKi = 8.7; ADRA2C pKi = 9.6). Moderate affinity of yohimbine is observed also against different subtypes of 5-HT receptors (5-HT_1A_ pKi = 7.3; 5-HT_1B_ pKi = 6.8; 5-HT_1D_ pKi = 7.6), toward ADRA1 receptors (ADRA1A pKi = 6.7; ADRA1B pKi = 6.8; ADRA1D pKi = 6.8), and toward the D2 receptor (pKi = 6.4), whereas the affinity toward the D3 receptor is negligible.^14^

A variety of side effects can be caused by antagonism of the mentioned off-target receptors. Blockage of ADRA1 receptors can lead to hypotension,^15,16^ which can worsen hypotension caused by acute sepsis^17^ and lead to ischemic insult to major organs.^18^ Other undesirable side effects include increased heart rate and tremulousness.^19^ ADRA1 blockers are also contraindicated if the patient has cerebrovascular disease, coronary artery disease, or current respiratory infection,^19^ where the last one is frequent for septic patients.^20^ Blockage of 5-HT_1_ receptors might have other undesirable effects that include serotonin syndrome,^21^ anxiety,^22^ and gastrointestinal disturbances such as nausea, vomiting, and diarrhea.^23^ There is less information about the selective inhibition of individual ADRA2 receptor subtypes. However, there is evidence of an antinociception effect of ADRA2B and C receptor agonists; thus, their blockage might be undesirable.^24^ Considering all possible side effects, the low selectivity of yohimbine represents a substantial obstacle for the application of yohimbine in clinical use.

An effective approach to improve the selectivity of a ligand is structure-based drug design on several receptors, where differences between receptors of interest are considered and chemical modifications of the ligand are made accordingly.^25−27^ In our study, we performed molecular docking of yohimbine (**1**) into available crystal structures (see below) of the receptors mentioned above, and we proposed chemical modifications of the ligand based on this analysis. A series of novel derivatives were synthesized, and their potency and basic selectivity were primarily tested in functional (calcium flux) assays using cells overexpressing ADRA2A or ADRA1A. The most promising compound **4n** was also tested for its selectivity over other ADRA2 family members and the 5-HT1 receptor. The prepared compounds were then evaluated for their cytotoxicity on noncancerous normal human dermal fibroblasts (NHDF), together with the metabolic stability, solubility, and intestinal and blood–brain barrier (BBB) permeability. The ultimate aim of this study was to identify a yohimbine analogue with improved properties as a potential preclinical candidate for treatment of sepsis and liver inflammation.^28^

## 2 Results and Discussion

### 2.1 Prediction of Ligand Selectivity for alpha-Adrenergic (ADRA) and Serotonin (5-HT) Receptors Using Molecular Docking

**Figure 2:**
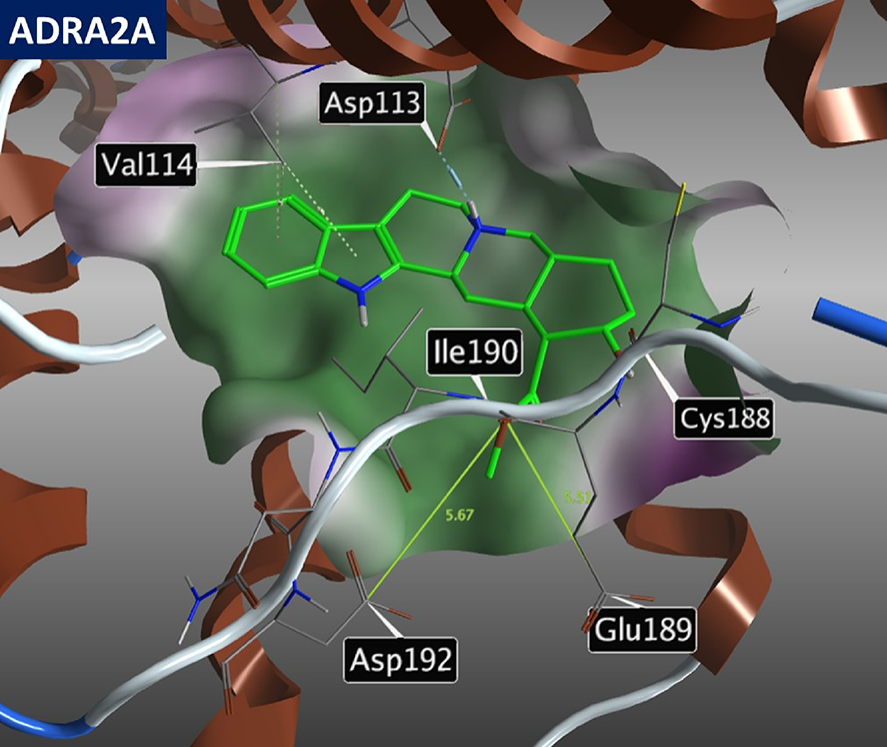
Image showing the molecular docking of yohimbine (**1**) into the binding pocket of ADRA2A (PDB 6KUX) and depicting key amino acids in close proximity to yohimbine.

To improve the selectivity of yohimbine derivatives toward the ADRA2 family of receptors over ADRA1 and 5-HT families, the differences in their binding pockets were examined. The pocket structure was determined for receptors with the following published crystal structures: ADRA2A (6KUX),^29^ ADRA2B (6K41),^30^ ADRA2C (6KUW),^31^ ADRA1A (AlphaFold model),^32^ and 5-HT_1A_ (7E2Z).^33^ No crystal structure in complex with an antagonist is published for the ADRA1A receptor; thus, the AlphaFold model of the human ADRA1A receptor was used for docking. The molecular docking of yohimbine into the ADRA2A binding pocket revealed four main interactions (Figure 2): interaction **A**, the salt bridge between the positively charged tertiary amine of yohimbine and the carboxylate of Asp 113; interaction **B**, the aromatic interaction between the ligand’s indole moiety and the hydrogen atom of Val 114; interaction **C**, the hydrogen bond between the hydroxy group of yohimbine and the amide group of Cys 188; and interaction **D**, the hydrogen bond between the ester group of yohimbine and the amide group of Ile 190.

**Figure 3:**
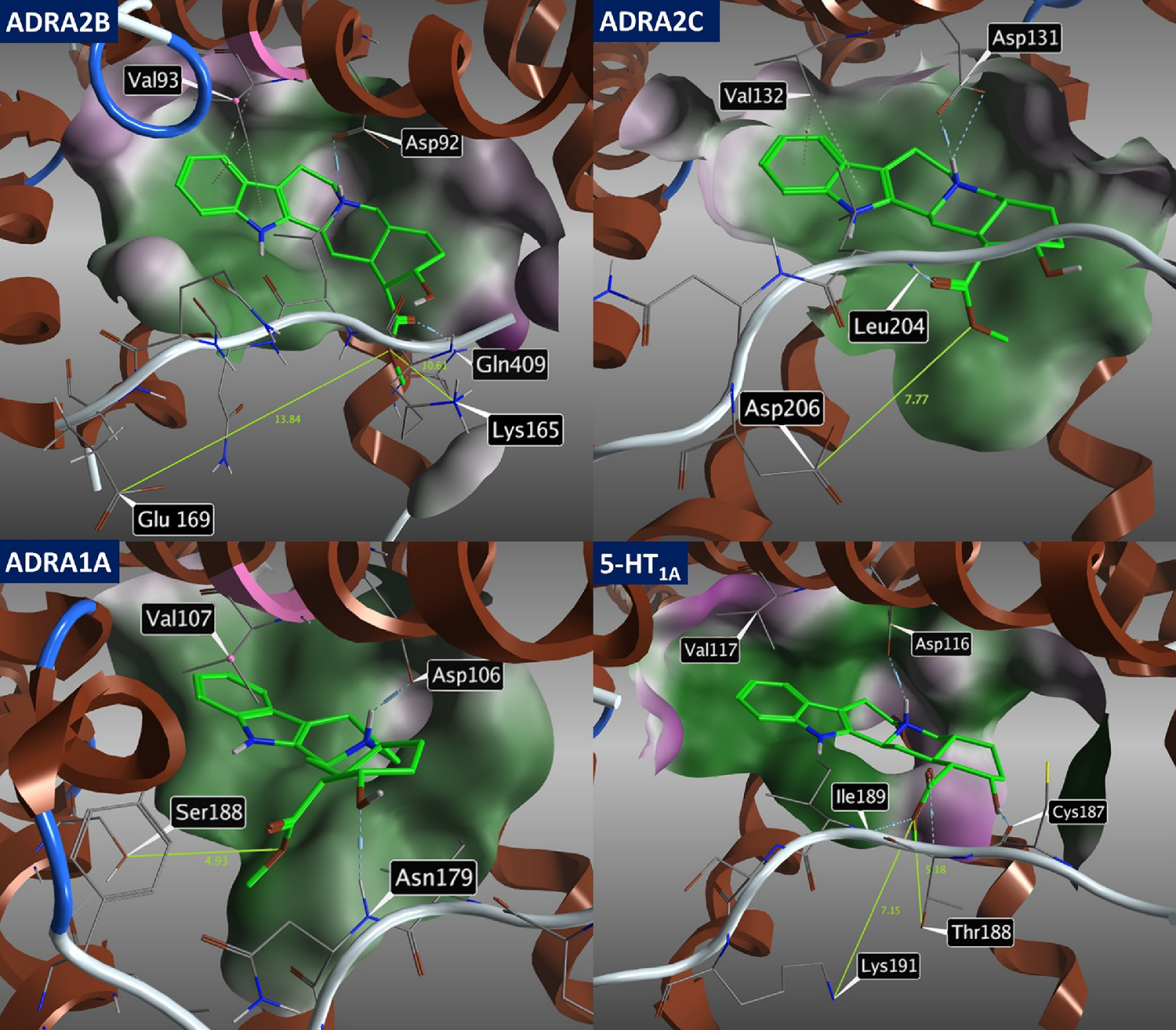
Image showing the molecular docking of yohimbine (**1**) into off-target receptors ADRA2B (6K41), ADRA2C (6KUW), ADRA1A (AlphaFold model), and 5-HT_1A_ (7E2Z) and depicting key amino acids in proximity to yohimbine.

The following interactions of yohimbine with major off-target receptors were also identified (Figure 3), namely, ADRA2B: interactions **A** (Asp 92) and **B** (Val93); ADRA2C: interactions **A** (Asp 131), **B** (Val132), and **D** (Leu 204); ADRA1A: interactions **A** (Asp 106) and **C** (Asn 179); and 5-HT_1A_: interactions **A** (Asp 116), **C** (Cys 187), and **D** (Ile 189).

Whereas the inner parts of the pockets are broadly similar, the outer parts show substantial differences in functional amino acid residues in the proximity of the yohimbine methyl ester group. These features can be exploited to increase the selectivity by introducing modifications of the yohimbine scaffold with the help of rational drug design. Table 1 demonstrates that receptors ADRA2A and ADRA2C have the most similar chemical space around the methyl ester moiety of yohimbine with the acidic residues being less than 10 Å away (14 Å for ADRA2B). This suggests that gaining ADRA2A vs ADRA2C selectivity may be quite problematic. In the corresponding space, other receptors under investigation have only basic or neutral amino acid residues within a 10 Å radius.

**Table 1:** Functional Amino Acid Residues for the Relevant Receptors and Their Distance from the Methyl Ester Group of Yohimbine (**1**)

Receptor type (PDB code)	AA residue (distance)
ADRA2A (6KUX)	Asp192 (5.7 Å), Glu189 (5.5 Å)
ADRA2B (6K41)	Glu169 (13.8 Å), Lys165 (10.6 Å)
ADRA2C (6KUW)	Asp206 (7.8 Å)
ADRA1A (AlphaFold)	Ser188 (4.9 Å)
5-HT_1A_ (7E2Z)	Lys191 (6.2 Å)

Given these differences, selectivity toward the ADRA2 family of receptors might be improved by elongating the methyl ester moiety of yohimbine using a linker ending with an extra amino group. In this scenario, an additional salt bridge between the newly added aliphatic amino group (protonated at the physiological pH) and the amino acid residues of the ADRA2A receptor has the potential to enhance the compound’s binding. On the other hand, the same change might not affect binding to ADRA1A and could lead to a weaker binding to 5-HT_1A_ due to repulsion between both positively charged amines and basic amino acid residue.

Although the three subtypes of the ADRA2 receptor family contain acidic amino acid residues (Asp and Glu; Table 1), the distance from the yohimbine methyl ester is different in each case. This offers a possibility of gaining some selectivity by optimizing both linker length and geometry. For binding of modified yohimbine (**1**) to ADRA2A, the optimal length of the linker between ester oxygen and the amino group was estimated to be two to three atoms long. Thus, the introduction of linkers with four-, five-, or six-membered cycles might afford additional binding specificity. Amide derivatives of yohimbine were not broadly explored, as they were reported to be approximately 2 orders of magnitude less potent on ADRA2 receptors than yohimbine, similarly to yohimbic acid.^34^

To explore the validity of the above-described hypotheses, compounds bearing hydrogen bond acceptors/donors (namely, ethero esters, amino esters, and amino amides), as well as structurally similar compounds with neutral functionality (aliphatic esters), were designed, synthesized, and evaluated for their biological activity.

### 2.2 Synthesis of Modified Yohimbines

**Scheme 1:**
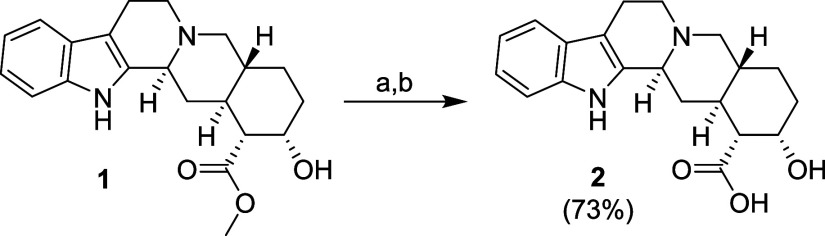
Synthesis of Yohimbic Acid (**2**) Reagents and conditions: (a) LiOH·H_2_O, H_2_O/dioxane (1:1), RT; (b) HCl, RT (to pH = 7).

The key starting material, yohimbic acid (**2**, Scheme 1), was prepared by hydrolysis of commercially available yohimbine hydrochloride under basic conditions.^35^ Yohimbic acid was isolated in its free form, which was needed for the subsequent Mitsunobu reaction.

**Scheme 2:**
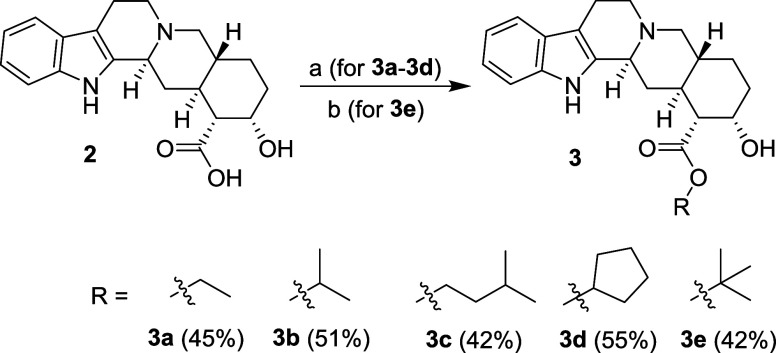
Synthesis of Yohimbic Acid Esters **3a**–**3e** Reagents and conditions: (a) H_2_SO_4_ (cat.), ROH, 78 °C; (b) *N*,*N*-dimethylformamide di-tert-butyl acetal, toluene, 90 °C.

Simple aliphatic esters **3a**–**d** (Scheme 2) were synthesized by the direct esterification of compound **2** in the appropriate alcohol under acidic conditions at elevated temperature. Compound **3e** (Scheme 2) was prepared by the reaction of compound **2** with *N*,*N*-dimethylformamide di*tert*-butyl acetal in toluene.

**Scheme 3:**
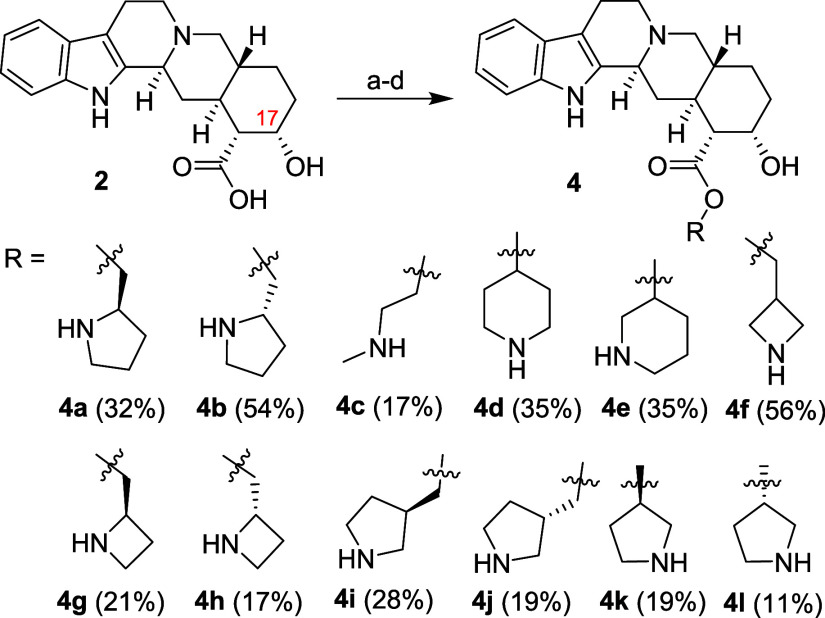
Synthesis of Yohimbic Acid Amino Esters **4a**–**4l** Reagents and conditions: (a) BSA, 1,5-diazabiciclo(5.4.0)undec-7-ene (DBU), anhydrous THF, 60 °C; (b) Ph_3_P, ROH (where R is *N*-Boc-protected amino alkyl), 60 °C; (c) DIAD, 60 °C; (d) TFA, 60 °C.

As the first series of esters containing an amino group (simply amino esters), compounds **4a**–**4l** (Scheme 3) were synthesized via the Mitsunobu reaction in a one-pot procedure. To prevent its elimination, the hydroxy group at the C-17 position of starting compound **2** was protected *in situ* with the trimethylsilyl (TMS) moiety using bis(trimethylsilyl)acetamide (BSA).^36^ In parallel, triphenylphosphine and *N*-Boc-protected amino alcohol (alkanolamine) were mixed, codistilled with toluene to remove moisture, dissolved in dry THF, and added to TMS-protected yohimbic acid in THF. The resulting mixture was heated to 60 °C, and DIAD was added dropwise.^37^ This sequence of reagent addition afforded the best overall yields (optimization not shown). After reaction completion, the solvent was evaporated, and the protecting groups were removed simultaneously by treatment with trifluoroacetic acid (TFA).^38^ This method afforded low-to-moderate yields of desired products **4a**–**4l** (Scheme 3) together with a small amount of the dehydration side product in several cases (not isolated, observed in UPLC–MS).

**Scheme 4:**
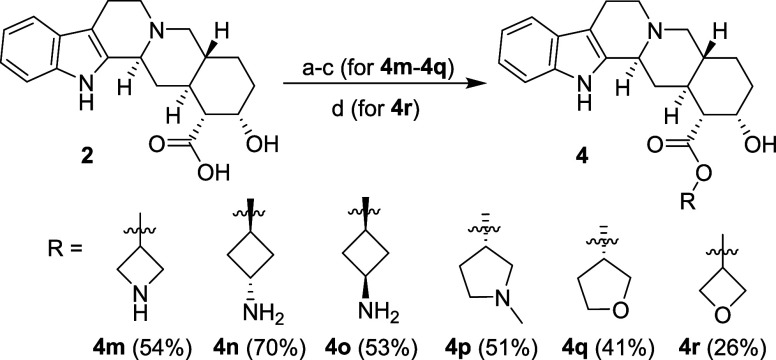
Synthesis of Yohimbic Acid Amino Esters **4m**–**4p** and Ethero Esters **4q** and **4r** Reagents and conditions: (a) Cs_2_CO_3_, DMF, 80 °C; (b) MsOR (where R is *N*-Boc protected amino alkyl or other functionalized alkyl with no acidic hydrogens; Ms is mesyl), Et_3_N, DMF, 110 °C; (c) TFA, 60 °C; (d) EDC·HCl, DMAP, CH_3_CN, under Ar, RT, 2.5 h.

Because the preparation of yohimbic acid esters by the above-described synthetic route using alcohols with the hydroxy group attached directly to a four-membered cycle proved to be problematic, a different synthetic approach was needed. *N*-Boc-protected amino alcohols were converted into alkylating agents using mesyl chloride and subsequently treated with the cesium salt of yohimbic acid (**2**, Scheme 4) according to described procedures.^39,40^ Removal of protecting groups and reaction workup were performed in the same way as above. This approach afforded good yields of target compounds **4m**–**4q** (Scheme 4), and their purification was also found to be easier. The synthesis of oxetane derivative **4r** by this method afforded only traces of the product (observed in UPLC–MS), and therefore, a new synthetic procedure was developed utilizing EDC·HCl and DMAP as a base, affording product **4r** in a 26% yield (Scheme 4).

**Scheme 5:**
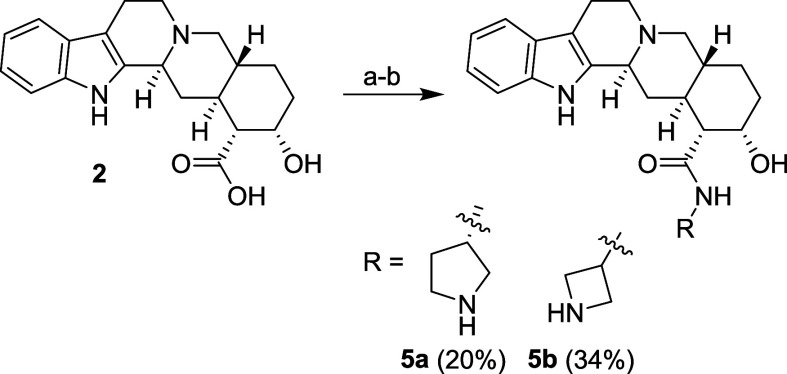
Synthesis of Yohimbic Acid Amino Amides **5a** and **5b** Reagents and conditions: (a) RNH_2_ (where R is *N*-Boc protected amino alkyl), DBU, HATU, DCM, RT; (b) evaporation until constant volume, TFA, 60 °C.

Amides containing an amino group (simply amino amides), **5a** and **5b**, were synthesized via the coupling reaction^41^ of compound **2** with properly protected diamines followed by removing the Boc-protecting group using TFA (Scheme 5).

**Scheme 6:**
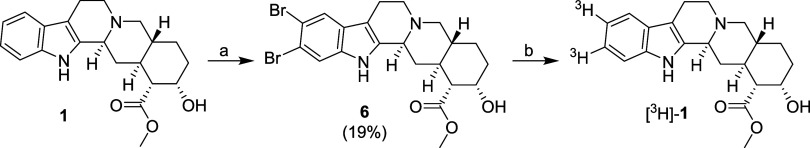
Synthesis of Tritiated Yohimbine ([^3^H]-**1**) Reagents and conditions: (a) Br_2_, FeCl_3_·6H_2_O, 0 °C, and CHCl_3_, 10 min; (b) ^3^H_2_, Pd/C (10%), TEA, MeOH, RT, 2.5 h.

To perform a radioligand binding assay, tritiated yohimbine ([^3^H]-**1**) was prepared (Scheme 6). First, yohimbine (**1**) was brominated to afford dibromo derivative **6** (19%), which upon tritiation in methanol gave the desired [^3^H]-yohimbine ([^3^H]-**1**).

### 2.3 ADRA2A and ADRA1A Antagonist Activity Assay

All prepared compounds were tested for their antagonistic activity using calcium flux assays on cells overexpressing the ADRA2A or ADRA1A adrenergic receptor as a primary screen (Table 2). A calcium-sensitive fluorescent probe (Fluo-8 AM) was used to monitor calcium flux following receptor stimulation with epinephrine upon pretreatment with tested compounds.

**Table 2:**
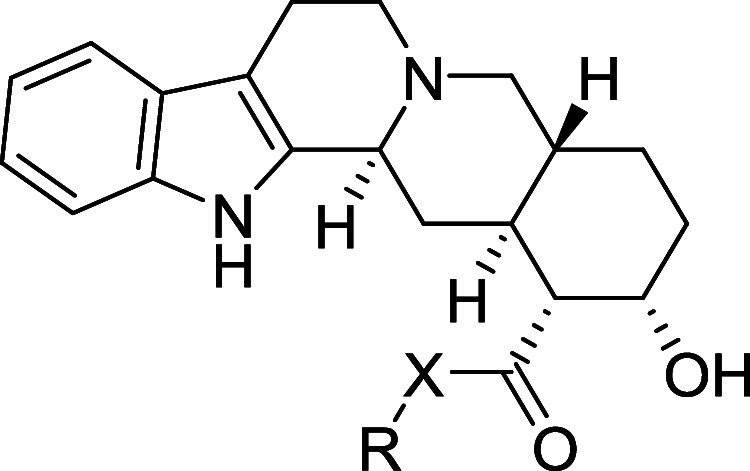

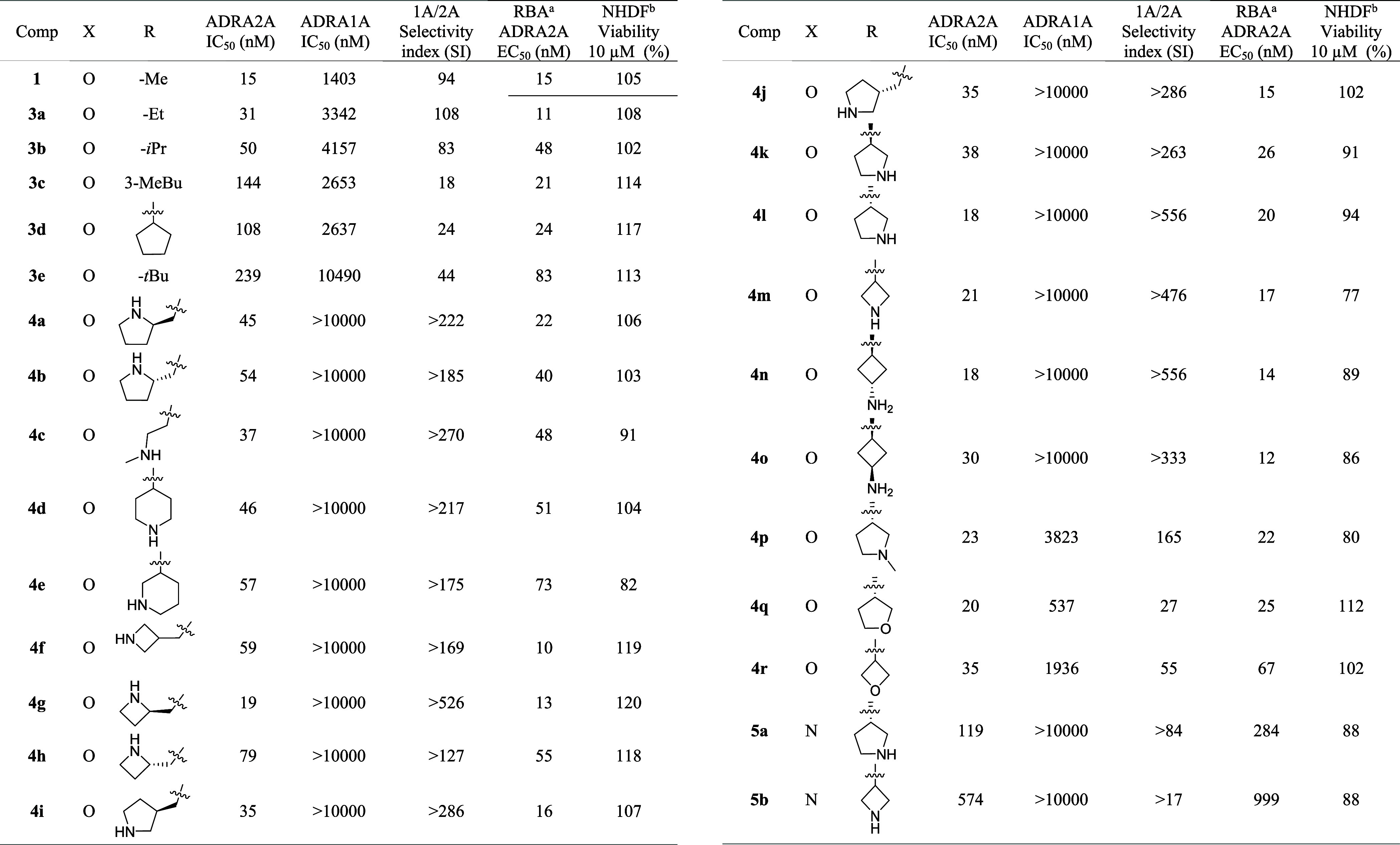
Structures and Antagonist Activity of Prepared Yohimbic Acid Derivatives **3a**–**3e**, **4a**–**4r**, **5a**, and **5b** on ADRA2A and ADRA1A Receptors Stimulated with EC_80_ of Epinephrine (Cell-Based Calcium Flux Assay), Radioligand Binding Cell-Based Assay (RBA) on ADRA2A, and Viability on Nontumor Normal Human Dermal Fibroblasts (NHDF) after 72 h at the Concentration 10 μM

a RBA, radioligand binding assay (cell-based).

b NHDF, normal human dermal fibroblasts.

All prepared alkyl esters of yohimbic acid, i.e., compounds **3a**–**3e** with no additional interaction in the binding pockets of the receptors, exhibited lower potency against both ADRA2A and ADRA1A as well as lower selectivity (except for ethyl ester **3a**) compared to parent yohimbine (Table 2). Increasing the ester group size (bulkiness) generally led to a decreasing ability to antagonize both ADRA2A and ADRA1A receptors.

On the contrary, all prepared amino esters (except for compound **4p**) displayed at least 7 times lower potency to antagonize the major off-target receptor, ADRA1A, compared to yohimbine (**1**). Given the comparable or slightly lower potency toward the desired target, ADRA2A, this structural modification resulted in a significantly improved selectivity index (SI, Table 2) of all amino esters **4a**–**4p** (SI in the range of 127–556) compared to yohimbine (SI = 94) tested in the same assay.

The antagonistic activity of amino esters on ADRA1A was found to be strongly dependent on the type of the R moiety of the ester groups and was much lower for amines than for corresponding ethers (namely, **4l** vs **4q** and **4m** vs **4r**, Table 2). This is probably because Ser188 of ADRA1A can form hydrogen bonds with the etheric oxygen atom but not with the protonated amino group.

On the other hand, ADRA2A antagonist activity of amino esters was not greater than that of parent yohimbine (**1**, Table 2). Although substantially improved affinity of amino esters to ADRA2A would be expected as a result of the additional ionic interaction between the introduced (protonated) amino group and Asp 192, the addition of the protonatable group concurrently led to increased solvation of yohimbic acid amino esters. These two effects most likely counteracted each other, thereby resulting in an unchanged overall potency in the assay. Nevertheless, a clear benefit of the addition of the secondary amino group on the selectivity index can be observed on the series of compounds bearing the five-membered ring: compounds bearing the pyrrolidine moiety (**4a**, **4b**, and **4i**–**4l**) exhibited SI values in the 185–556 range, whereas cyclopentyl derivative **3d** (SI = 24) and tetrahydrofuranyl derivative **4q** (SI = 27) exhibited substantially lower SI.

Also, the antagonist activity toward ADRA2A was more dependent on the type of the carboxylic acid derivative and was much higher for esters compared to analogous amides (**4l** vs **5a** and **4m** vs **5b**, Table 2). The carbonyl group of these derivatives participates in hydrogen bonding with NH of Ile 190, and ester is a better hydrogen bond acceptor than the corresponding amide. Thus, the highest SI was observed for the compounds that were designed to combine both secondary amine and ester group in their structure.

According to our rational drug design, the repulsion between Asp 113 of the ADRA2A receptor and the etheric group should strongly disrupt the activity of ethero esters. However, the results showed they were slightly less potent than their corresponding amino esters (**4l** vs **4q**; **4m** vs **4r**, Table 2). In these cases, the repulsion may have been weakened by the relative flexibility of both the outer part of ADRA2A receptor as well as the ligand. Moreover, unlike protonated amino esters, ethero esters do not have to overcome a strong solvation effect, which might also improve their potency.

Importantly, the structural character of the substituents also substantially affected the biological activity of the compounds. Compared to amino esters with an acyclic linker (compound **4c**) and six-membered rings (**4d** and **4e**), most of the derivatives with more rigid five- or four-membered rings, and often with defined stereochemistry, had a substantially higher ADRA1A/ADRA2A selectivity index (**4g**, **4l**, **4m**, **4n**, **4o**). Moreover, different potencies of some diastereomeric pairs [e.g., **4g** vs **4h** (∼4-fold), **4k** vs **4l** (∼2-fold), and **4n** vs **4o** (∼1.7-fold)] on ADRA2A suggested that the receptor pocket might be sensitive to steric effects and to the absolute configuration of the amino ester moiety.

Overall, our results are in good correlation with our key hypothesis that the selectivity of yohimbic acid amino esters **4a**–**4p** (SI in the range of 127–556) for ADRA2A over ADRA1A was higher than that of parent yohimbine (**1**, SI = 94) and of other simple alkyl esters **3a**–**3e** (SI in the range of 18–108). Clearly, not only attractive and repulsive interactions within the receptors’ pockets but also the solvation effect (which was supposed to be stronger for protonatable amino esters and amides) influenced the biological activity of the prepared compounds.

To exclude compounds with unwanted agonistic effects that could be eventually masked by the presence of epinephrine in a standard antagonist experimental setup of calcium flux assay, all tested compounds were concurrently screened for potential agonistic effects on ADRA2A and ADRA1A, i.e., in the absence of epinephrine. None of the tested compounds proved to have an agonistic mode of action on ADRA2A and ADRA1A in addition to their antagonist effects (data not shown).

In addition to the primary functional screen (calcium flux assay), competitive radioligand binding cell-based assay (RBA) was employed as an orthogonal assay to validate the hits and to confirm their direct interaction with the ADRA2A receptor. The calculated EC_50_ values were found to correlate well with the IC_50_ values obtained from the calcium flux assay (Table 2). Dissociation constants (*K*_D_'s) calculated for yohimbine and for lead compound **4n** were 4.8 and 8.8 nM, respectively. Very similar *K*_D_ values were also obtained from SPR microscopy on live cells (8.1 nM for yohimbine and 28.2 nM for **4n**).

Finally, the compounds showed no or very little toxicity on nontumor normal human dermal fibroblasts (NHDF) after 72 h at the concentration 10 μM (Table 2).

### 2.4 Off-Target Receptor Selectivity Screening and Molecular Docking

**Figure 4:**
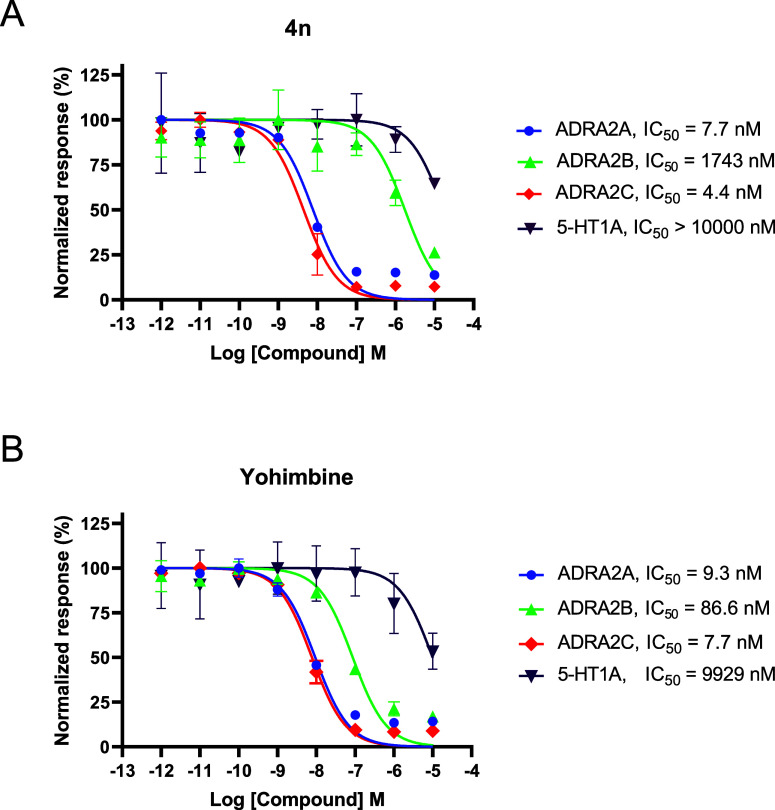
Selectivity profile of compound **4n** (A) and yohimbine (B) on closely related α2-adrenergic (ADRA2A, 2B, and 2C) and serotonin receptors (5-HT1A). Calcium flux assay was performed on cell lines stably expressing the receptors of interest (CHO Gqi5 ADRA2A, CHO Gqi5 ADRA2B, CHO Gqi5 ADRA2C, HEK Gqi5 5-HT_1A_) in the presence of the EC_80_ reference agonist, i.e., brimonidine (2A, 2B, 2C) or serotonin (5-HT_1A_). Data are expressed as % response vs the effect of an agonist (100% = EC_80_ agonist).

Although the main screening strategy was primarily based on determining the selectivity between ADRA2A and ADRA1A receptors, a representative compound with the most favorable selectivity index, i.e., compound **4n** with SI > 556, was also tested for its agonist and antagonist off-target effects toward other ADRA2 family members, namely, 2B and 2C, and a selected serotonin (5-HT1) receptor to assess the broader selectivity profile of this novel class of compounds (Figure 4). Yohimbine (**1**) was used as the reference compound.

The obtained data suggest that both yohimbine and compound **4n** have comparable antagonistic potency (and thus similar SI values) on ADRA2A, ADRA2C, and 5-HT_1A_ receptors. The major difference was observed for ADRA2B, where compound **4n** exhibited 20 times lower potency (compared to yohimbine) and thus much higher (24 times) ADRA2B/ADRA2A selectivity (SI = 226 for **4n**, SI = 9.3 for yohimbine).

**Figure 5:**
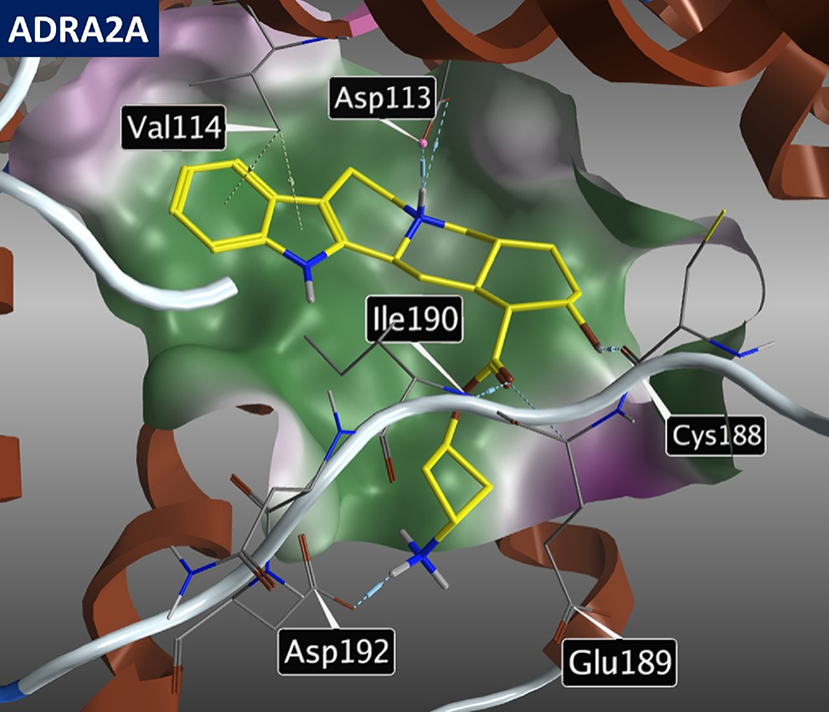
Image showing the molecular docking of compound **4n** into target receptor ADRA2A (6KUX) and depicting key amino acids in close proximity to **4n**.

**Figure 6:**
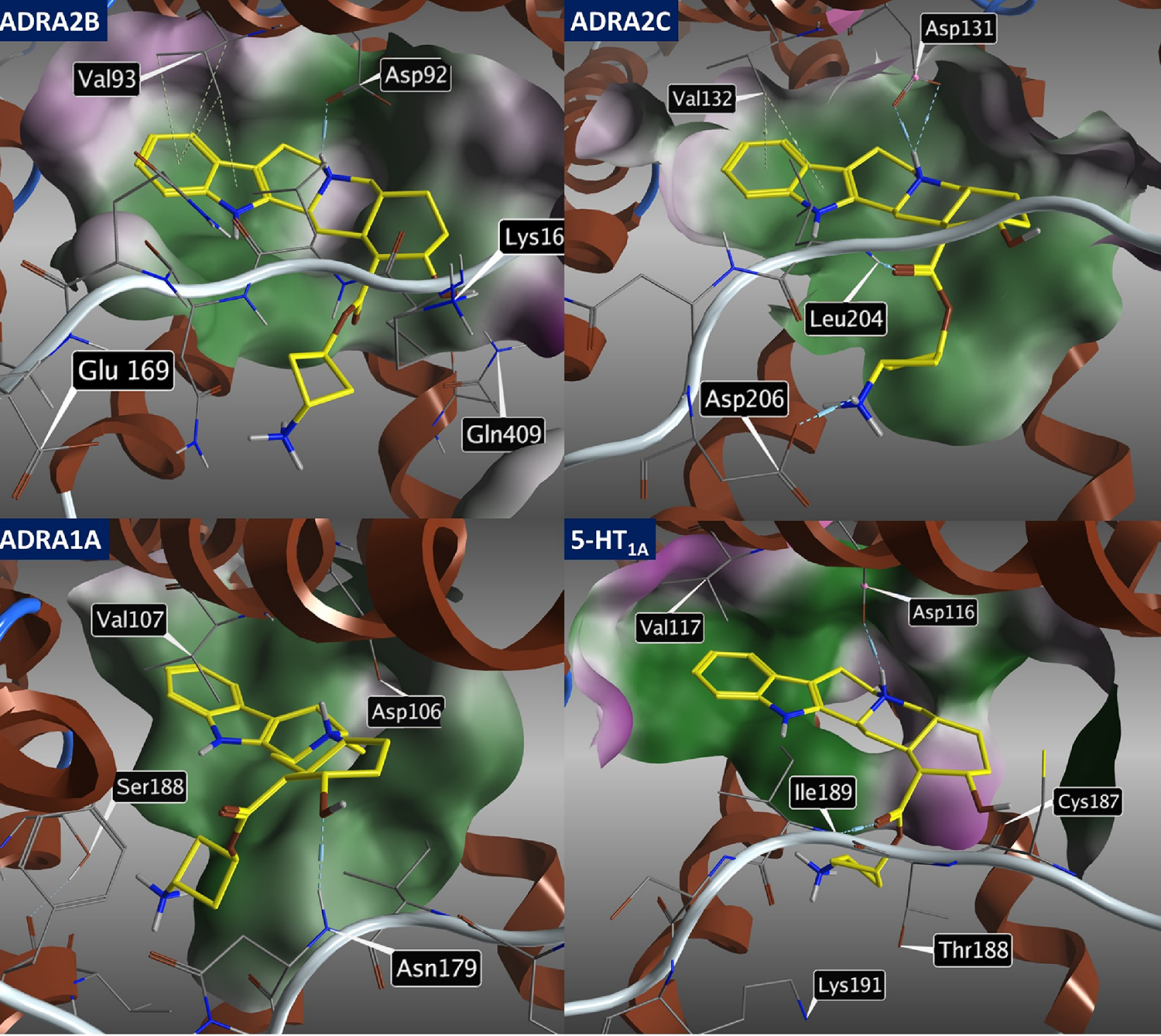
Image showing the molecular docking of compound **4n** into off-target receptors ADRA2B (6K41), ADRA2C (6KUW), ADRA1A (AlphaFold model), and 5-HT_1A_ (7E2Z) and depicting key amino acids in close proximity to **4n**.

Furthermore, to reveal the subtle differences in the binding modes, the molecular docking of compound **4n** to the active sites of selected receptors has been performed as well using the published crystal structures of ADRA2A (6KUX)^29^ (Figure 5) and of off-target receptors ADRA2B (6K41),^30^ ADRA2C (6KUW),^31^ ADRA1A (AlphaFold model),^32^ and 5-HT_1A_ (7E2Z)^33^ (Figure 6).

According to our expectations, no substantial difference in binding of compound **4n** toward ADRA2A (Figure 5) and ADRA2C (Figure 6) was observed. This could be explained by the formation of an additional salt bridge between **4n** and Asp 192 (ADRA2A) and between **4n** and Asp 206 (ADRA2C). Such an intermolecular interaction cannot be observed in the case of other receptors evaluated in this study (Figure 6). On the other hand, the increased solvation effect due to the primary amine of **4n** and no extra interaction gained resulted in a marked loss in the compound’s potency toward ADRA2B and ADRA1A receptors (Figure 4). Also, the pocket size might play a significant role, as 5-HT_1A_ did not gain any specific interaction with **4n** (Figure 6), but unlike small pockets of ADRA2B and ADRA1A, it could better accommodate compound **4n**, and the affinity was reduced only slightly (Figure 4). Overall, while keeping the same potency toward the main target (ADRA2A), novel compound **4n** clearly outperformed yohimbine in terms of higher selectivity in the case of both ADRA1A (Table 2) and ADRA2B (Figure 4) receptors.

### 2.5 *In Vitro* ADME Profiling

To evaluate the drug-likeness of novel yohimbine analogues, metabolic stability in plasma (human and rodent) and microsomes, as well as membrane permeability through the monolayer of Caco-2 cells, was determined (Table 3). All of the evaluated compounds exhibited high stability in both plasma and liver microsomes. Also, no substantial differences between the human and rat materials were observed. Interestingly, the plasma stability of derivatives with more bulky ester groups (e.g., *i*Pr ester **3b** and most of the amino esters **4**), as well as of amino amides (namely compound **5a**), was not improved compared to parent yohimbine, i.e., the methyl ester **1**.

**Table 3:** Metabolic Stability and Intestinal Permeability of Yohimbine (**1**) and Its Selected Derivatives

	Plasma stability		Microsomal stability		Caco-2 permeability			
Comp	% remaining at 6 h		CL_int_ (μL/min/mg)		*P*_app_ ×10^–6^ (cm/s)		efflux ratio	recovery (%)
	hum	rat	hum	rat	A–B	B–A		
**1**	106 ± 0	90 ± 2	7 ± 1	2 ± 0	41 ± 12	23 ± 8	1	145
**3a**	109 ± 1	52 ± 0	14 ± 0	3 ± 0	n.d.^a^	n.d.	n.d.	n.d.
**3b**	102 ± 0	89 ± 0	17 ± 1	3 ± 1	n.d.	n.d.	n.d.	n.d.
**4a**	88 ± 0	68 ± 0	0	0	n.d.	n.d.	n.d.	n.d.
**4c**	91 ± 5	80 ± 1	0	0	n.d.	n.d.	n.d.	n.d.
**4d**	100 ± 1	87 ± 0	0	0	1 ± 0	2 ± 0	4	101
**4g**	118 ± 3	134 ± 1	4 ± 1	1 ± 1	n.d.	n.d.	n.d.	n.d.
**4i**	88 ± 3	62 ± 7	4 ± 1	1 ± 1	n.d.	n.d.	n.d.	n.d.
**4j**	92 ± 0	76 ± 3	2 ± 0	1 ± 1	n.d.	n.d.	n.d.	n.d.
**4k**	95 ± 5	110 ± 3	3 ± 2	1 ± 1	1 ± 0	0	0	101
**4l**	100 ± 0	80 ± 1	0	0	1 ± 0	0	0	112
**4m**	100 ± 3	70 ± 2	3 ± 2	0	1 ± 0	0	0	101
**4n**	99 ± 0	84 ± 2	2 ± 1	0	3 ± 1	0	0	103
**4o**	97 ± 5	75 ± 0	2 ± 1	3 ± 0	1 ± 0	0	0	101
**4p**	109 ± 2	85 ± 2	10 ± 0	1 ± 1	n.d.	n.d.	n.d.	n.d.
**4q**	118 ± 0	102 ± 1	15 ± 1	5 ± 1	n.d.	n.d.	n.d.	n.d.
**4r**	94 ± 14	89 ± 1	16 ± 1	2 ± 1	n.d.	n.d.	n.d.	n.d.
**5a**	100 ± 1	114 ± 2	0	0	n.d.	n.d.	n.d.	n.d.

a n.d. = not determined.

A Transwell system consisting of two compartments separated by a monolayer of differentiated Caco-2 cells was employed for the intestinal permeability evaluation. Thanks to the ability of Caco-2 cells to become polarized, bidirectional transport (A–B and B–A) could be measured and the efflux ratio calculated. Although only a subset of novel compounds (**4d**, **4k**, and **4m**–**4o**) was assayed in the Caco-2 experiment, we observed that all analogues tested displayed about an order of magnitude worse permeability coefficient compared to yohimbine (Table 3). Except for compound **4d**, this apparent drop in permeability was not due to the active efflux from the cells but presumably corresponds to an increased polarity of the compounds containing protonatable amino group, i.e., the amino ester analogues of yohimbine (**1**). Although the low Caco-2 permeability may negatively affect the bioavailability (especially when administered orally), more polar compounds, in general, may display improved aqueous solubility. Nevertheless, it was demonstrated that all studied compounds (i.e., yohimbine and its novel analogues) were highly soluble at pharmacologically relevant concentrations (Table S1). Therefore, variations in their biological properties cannot be attributed to different aqueous solubility.

The increased polarity of prepared yohimbine analogues may also lead to a lower ability to penetrate the blood–brain barrier (BBB) and cause fewer central nervous system side effects while still possessing activity in the periphery. It should be noted that centrally acting ADRA2A antagonists also have therapeutic implications, e.g., as antidepressants.^42,43^ However, we aimed to develop drug candidates for sepsis and inflammatory liver failure, i.e., peripherally acting drugs. To investigate the ability to cross the BBB, compound **4n** and yohimbine (**1**) were evaluated in the MDCKII-MDR1 assay. Permeability assays based on canine MDCK cells with or without overexpression of human MDR1 (P-gp) protein are broadly accepted as cell-based predictors of the BBB permeation.^44,45^ The permeability of **4n** was markedly lower than that of yohimbine in MDCKII-MDR1 cells (Table 4). This is consistent with the permeability coefficients (*P*_app_) measured in the Caco-2 assay (Table 3) that have been reported to correlate well with the *P*_app_ gained from the MDCK assay.^44^ Furthermore, compound **4n** seems to be a substrate for the P-gp transporter, which is physiologically highly expressed in the BBB. Thus, the action of the novel yohimbine analogues should remain restricted to the periphery.

**Table 4:** Estimation of BBB Permeability and P-gp-Mediated Efflux for Compound **4n** and Yohimbine (**1**) in MDCKII-MDR1 Cells

Comp	*P*_app_ (cm/s) A–B × 10^–6^	recovery (%)	*P*_app_ (cm/s) B–A × 10^–6^	recovery (%)	efflux ratio
**1**	44.4 ± 8.3^a^	104	26.8 ± 0.2^a^	88	0.6
**4n**	0.9 ± 0.0	100	3.5 ± 1.4	98	3.9

a Data represent means ± SD of two independent experiments performed in triplicates.

## 3 Conclusions

In this work, we employed structure-based rational drug design and molecular docking together with a set of biological assays (functional, binding, and ADME) to identify new yohimbine derivatives exhibiting high antagonist potency against ADRA2A adrenergic receptor with improved selectivity over related off-target receptors. The long-term goal is to use such compounds to treat noradrenaline-mediated peripheral inflammatory disorders such as septic shock and organ failure.

Novel yohimbine derivatives with the desired properties have been identified. The most promising series was represented by amino esters of yohimbic acid with small rigid cycles on the ester moiety and defined stereochemistry. Compared to parent yohimbine, compounds **4g**, **4l**, **4m**, and **4n** had at least 5 times higher ADRA1A/ADRA2A selectivity index. Moreover, **4n** displayed 25 times higher ADRA2B/ADRA2A selectivity index compared to yohimbine, while 5-HT_1A_ remained largely unaffected. It was also discovered that a replacement of the amino group (compounds **4l** and **4m**) for the etheric group (compounds **4q** and **4r**, respectively) significantly increased off-target effects toward ADRA1A and that utilization of amides (compounds **5a** and **5b**) instead of esters (compounds **4q** and **4r**, respectively) led to the loss of potency toward the target ADRA2A receptor.

The most promising compound **4n** demonstrated high stability in both human and rat plasma and liver microsomes, low toxicity on normal human dermal fibroblasts, and a diminished ability to cross the blood-brain barrier. Thus, compound **4n** represents a novel pharmacological probe to investigate the involvement of various adrenoceptors in relevant indications and, at the same time, a promising lead candidate for further preclinical development to treat sepsis-induced liver inflammation and other ADRA2A-mediated pathologies. Compound **4n** is a great example of rational drug design starting from the structure of the naturally occurring alkaloid.

## 4 Experimental Section

### 4.1 Chemistry

#### 4.1.1 General Methods

Unless otherwise stated, solvents were evaporated at 40 °C/2 kPa, and prepared compounds were dried at 30 °C at 2 kPa. Starting compounds and reagents were purchased from commercial suppliers (Sigma-Aldrich, Fluorochem, Acros Organics, TCI, and AmBeed) and used without further purification or were prepared according to the published procedures. Tetrahydrofuran, dioxane, and acetonitrile were dried by activated neutral alumina (drysphere). Dimethylformamide was dried by activated molecular sieves (3 Å). Other dry solvents were purchased from commercial suppliers (Sigma-Aldrich and Acros Organics). Triethylamine was dried over potassium hydroxide under an argon atmosphere in a dark flask sealed with a septum.

Flash column chromatography was carried out by Teledyne ISCO Grace with a dual absorbance detector on Teledyne ISCO columns RediSepRf HP C18 Aq GOLD in sizes 50 and 100 g. Eluents used were methanol, acetonitrile, and water. Preparative LC purifications were performed on a Waters Delta 600 chromatography system with columns packed with C18 reversed phase resin Phenomenex Gemini 10 μm 21 × 250 mm. Mass spectra, UV absorbance, and purity of compounds were measured on a Waters UPLC–MS system consisting of a Waters UPLC H-Class Core System (column Waters Cortecs UPLC C18 1.6 μm, 2.1 × 50 mm), Waters Acquity UPLC PDA detector, and mass spectrometer Waters SQD2. The universal LC method (eluent H_2_O/CH_3_CN with 0.1% of formic acid in both mobile phases, gradient 0–100%, run length 3.5 min) and the MS method (ESI+ and/or ESI– cone voltage = 30 V, mass detector range 100–1000 Da) were used. NMR spectra were recorded on a Bruker Avance III HD 500 MHz spectrometer equipped with a cryoprobe referenced to the residual solvent signal (DMSO-*d*_6_: δ(^1^H) = 2.50 and δ(^13^C) = 39.52, CDCl_3_: δ(^1^H) = 7.26 and δ(^13^C) = 77.16) or a specified additive to water samples (*t*-butanol: δ(^1^H) = 1.24 and δ(^13^C) = 30.29, ethanol: δ(^1^H) = 1.17 and δ(^13^C) = 17.47). The numbering system for ^1^H NMR and ^13^C NMR spectra of prepared compounds is shown for compound **4a** (Figure S1). High-resolution mass spectra were measured on a LTQ Orbitrap XL spectrometer (Thermo Fisher Scientific).

Yields were determined based on the amount of isolated compound. All compounds were >95% pure by HPLC analysis. Purity was determined by UPLC–PDAMS in combination with NMR and HR-MS data.

The titration reaction was performed in the customized stainless manifold system (RC Tritec). Evaporation at 40 °C/5 Pa (0.05 mbar) was done on the Centrivap Vacuum Concentrator/Ultralow cold trap system (Labconco). The radio-HPLC separations were done on the Alliance e2695 module equipped with a 2998 PDA detector (Waters) and operated by the Empower 3 Pro software. Radiochromatograms were recorded by the β^–^ radioactivity HPLC flow detector Ramona Star equipped with the LS-pump (Elysia-Raytest). Liquid scintillation measurements were done on a Tri-Carb 2900 liquid scintillation counter (PerkinElmer). LSC and flow LSC were performed in Rotiszint eco plus (Carl Roth) and Gold Flow (Meridian Biotech.) cocktails, respectively. ESI-MS(+) spectra of the labeled compound were measured on the SQ Detector 2 system (Waters) operated by the MassLynx 4.2 software. The ^3^H NMR spectra were recorded at 25 °C with the Avance II 300 MHz spectrometer (Bruker BioSpin) at 320.1 MHz.

#### 4.1.2 General Procedure for the Synthesis of Aliphatic Esters of Yohimbic Acid **3a**–**3d** via Fischer Esterification (Method A)

Yohimbic acid (**2**, 100 mg, 0.29 mmol) was suspended in the appropriate alcohol (3.0 mL), several drops of H_2_SO_4_ (cat.) were added at RT, and the reaction mixture was heated to 78 °C. When the starting compound was consumed, the reaction mixture was evaporated, and the residue was dissolved in DCM and extracted with saturated aqueous solution of NaHCO_3_. The organic layer was separated and dried over Na_2_SO_4_, and volatiles were removed *in vacuo*. The residue was adsorbed on C18, applied on flash chromatography column, and separated (RP-C18aq, eluent water/CH3CN, gradient 0–100%).

#### 4.1.3 General Procedure for the Synthesis of Amino Esters of Yohimbic Acid **4a**–**4l** via Mitsunobu Reaction (Method B)

Yohimbic acid (200 mg, 0.59 mmol) was suspended in toluene (10 mL), and the solvent was distilled off on RVO to dry the starting material. The material was dissolved in dry THF (4.0 mL), and DBU (98 mg, 0.65 mmol) and BSA (132 mg, 0.65 mmol) were added. The reaction was stirred for 1.5 h at 60 °C under an argon atmosphere. Concurrently, Ph_3_P (339 mg, 1.29 mmol) and *N*-Boc protected amino alcohol (0.88 mmol) were mixed and dried via codistillation with toluene (10 mL). This mixture was dissolved in THF (3.0 mL) and added to the first reaction mixture. The resulting mixture was heated to 60 °C, and DIAD (0.25 mL, 1.29 mmol) was added dropwise. After 30 min, THF was evaporated to constant volume, TFA (2.0 mL) was added, and the mixture was stirred for 60 min at RT until full removal of the Boc protecting group. TFA was partially distilled off, the residue was dissolved in DMF, applied on flash chromatography column, and separated (RP-C18aq, eluent water/methanol, gradient 0–100%) to obtain the product.

#### 4.1.4 General Procedure for the Synthesis of Yohimbic Acid Derivatives **4m**–**4q** via Alkylation Reaction (Method C)

*N*-Boc-protected amino alcohol (0.29 mmol) was dissolved in DMF (1.0 mL), TEA (0.05 mL, 0.35 mmol) was added, and MsCl (36 mg, 0.32 mmol) was then added dropwise at RT. The reaction mixture turned into a suspension and was stirred for another 50 min. Concurrently, yohimbic acid (**2**, 98 mg, 0.29 mmol) was dissolved in DMF (2.0 mL), and Cs_2_CO_3_ (127 mg, 0.39 mmol) was added. The mixture was stirred at RT for 40 min, and then the first reaction mixture was added to the second one. The temperature was increased to 110 °C, and the reaction mixture was stirred until full conversion. Afterward, the reaction mixture was evaporated using a cold finger until dryness. TFA (2.0 mL) was added to the residue, and the mixture was stirred at RT for 60 min until full removal of the Boc protecting group. TFA was partially distilled off, and the residue was dissolved in DMF, applied on flash chromatography column, and separated (RP-C18aq, eluent water/methanol, gradient 0–100%) to obtain the product.

#### 4.1.5 General Procedure for the Synthesis of Amino Amides of Yohimbic Acid **5a** and **5b** via Coupling Reaction (Method D)

Yohimbic acid (**2**, 120 mg, 0.35 mmol) was suspended in DCM (8.0 mL). Dry DBU (161 mg, 1.05 mmol) and mono *N-*Boc protected diamine (0.53 mmol) were added to the mixture at RT. Afterward, HATU (201 mg, 0.53 mmol) was added in portions, and the mixture was stirred at RT for another 2 h. The reaction mixture was evaporated to dryness. TFA (2.0 mL) was added to the residue, and the mixture was stirred for 60 min at RT until full removal of the Boc protecting group. TFA was partially distilled off, and the residue was dissolved in DMF, applied on flash chromatography column, and separated (RP-C18aq, eluent water/methanol, gradient 0–100%) to obtain the product.

#### 4.1.6 Yohimbic Acid (**2**)

Yohimbine hydrochloride (10 g, 28.21 mmol) was partially dissolved in a mixture of water/dioxane (70 mL/70 mL) at RT, and then LiOH.H_2_O (3.55g, 84.64 mmol, 3 equiv) was added. The mixture was stirred at RT for 2 h. Afterward 35% HCl_aq_ was added dropwise until neutral pH. Dioxane was evaporated from the mixture, and the rest of the mixture was applied on a flash chromatography column and separated (RP-C18aq, eluent water/methanol, gradient 0–100%) to obtain **2** (7.02 g, 73%) as a white solid. Spectral data correspond with the literature.^46^ MS (ESI+): *m*/*z* = 355.35 [M + H]^+^.

#### 4.1.7 17-α-Hydroxyyohimban-16-α-carboxylic Acid Ethyl Ester (**3a**)

Following method A, the treatment of **2** with ethanol (3.0 mL) overnight afforded **3a** (48 mg, 45%) as a white solid. ^1^H NMR (500 MHz, DMSO-*d*_6_): δ 7.94 (bs, 1H, H1), 7.45 (d, ^3^*J* = 7.7 Hz, 1H, H9), 7.30 (d, ^3^*J* = 8.0 Hz, 1H, H12), 7.12 (m, 1H, H11), 7.07 (m, 1H, H10), 4.32–4.19 (m, 3H, H17 and C**H**_2_–CH_3_), 3.37 (m, 1H, H3), 3.11 (m, 1H, H5a), 3.04–2.95 (m, 2H, H6a and H21a), 2.72 (m, 1H, H6b), 2.64 (m, 1H, H5b), 2.32 (m, 1H, H16), 2.27 (m, 1H, H21b), 2.05–1.96 (m, 3H, H14a, H15 and H18a), 1.60–1.51 (m, 3H, H18b, H19a and H20), 1.43–1.36 (m, 2H, H14b and H19b), 1.33 (t, ^3^*J* = 7.1 Hz, 3H, CH_3_) ppm. ^13^C{^1^H} NMR (126 MHz, DMSO-*d*_6_): δ 175.0 (COO), 135.7 (C13), 134.1 (C2), 127.1 (C8), 121.2 (C11), 119.2 (C10), 117.9 (C9), 110.6 (C12), 107.9 (C7), 66.7 (C17), 61.0 (C21), 60.7 (**C**H_2_–CH_3_), 59.7 (C3), 52.6 (C5), 52.1 (C16), 40.3 (C20), 36.4 (C15), 33.9 (C14), 31.2 (C18), 23.1 (C19), 21.4 (C6), 14.1 (CH_3_) ppm. HRMS (ESI+): *m*/*z* [M + H]^+^ calculated for C_22_H_29_O_3_N_2_ = 369.2173, found: 369.2172.

#### 4.1.8 17-α-Hydroxyyohimban-16-α-carboxylic Acid Isopropyl Ester (**3b**)

Following method A, treatment of **2** with isopropanol (3.0 mL) overnight afforded **3b** (57 mg, 51%) as a white solid. ^1^H NMR (500 MHz, DMSO-*d*_6_): δ 10.92 (bs, 1H, H1), 7.39 (bd, ^3^*J* = 7.8 Hz, 1H, H9), 7.31 (bd, ^3^*J* = 8.1 Hz, 1H, H12), 7.05 (m, 1H, H11), 6.97 (m, 1H, H10), 5.00 (sep, ^3^*J* = 6.2 Hz, 1H, C**H**–CH_3_), 4.54 (bs, 1H, OH), 4.14 (bs, 1H, H17), 3.39–2.91 (m, 6H, H3, H5, H6a and H21), 2.77 (m, 1H, H6b), 2.58 (m, 1H, H14a), 2.23 (dd, *J*_16,15_ = 11.5 Hz, *J*_16,17_ = 2.7 Hz, 1H, H16), 2.02 (m, 1H, H15), 1.78 (m, 1H, H18a), 1.61–1.53 (m, 2H, H18b and H20), 1.45 (m, 1H, H19a), 1.31–1.16 (m, 8H, H14b, H19b and CH_3_) ppm. ^13^C{^1^H} NMR (126 MHz, DMSO-*d*_6_): δ 171.2 (COO), 136.2 (C13), 126.1 (C8), 120.8 (C11), 118.5 (C10), 117.5 (C9), 111.1 (C12), 105.6 (C7), 66.5 (**C**H–CH_3_), 66.3 (C17), 51.5 (C16), 34.6 (C15), 32.1 (C18), 22.3 (C19), 21.7 and 21.4 (CH_3_) ppm. HRMS (ESI+): *m*/*z* [M + H]^+^ calculated for C_23_H_31_O_3_N_2_ = 383.2329, found: 383.2329.

#### 4.1.9. 17-α-Hydroxyyohimban-16-α-carboxylic Acid (3-Methylbutyl-1-yl) Ester (**3c**)

4.1.9

Following method A, treatment of **2** with isoamyl alcohol (4.0 mL) for 5 h afforded **3c** (51 mg, 42%) as a white solid. ^1^H NMR (500 MHz, DMSO-*d*_6_): δ 10.77 (bs, 1H, H1), 7.32 (m, 1H, H9), 7.25 (m, 1H, H12), 6.98 (m, 1H, H11), 6.91 (m, 1H, H10), 4.59 (d, ^3^*J* = 4.6 Hz, 1H, OH), 4.17–4.04 (m, 3H, H17 and O–CH_2_), 3.22 (m, 1H, H3), 2.98 (m, 1H, H5a), 2.83 (dd, ^2^*J* = 11.0 Hz, ^3^*J* = 3.1 Hz, 1H, H21), 2.75 (m, 1H, H6a), 2.58 (m, 1H, H6b), 2.47 (m, 1H, H5b), 2.42 (m, 1H, H14a), 2.23 (dd, *J*_16,15_ = 11.5 Hz, *J*_16,17_ = 2.8 Hz, 1H, H16), 2.11 (t, ^2^*J* = ^3^*J* = 10.5 Hz, 1H), 1.85 (m, 1H, H15), 1.75 (m, 1H, H18a), 1.70 (m, 1H, C**H**–CH_3_), 1.58–1.49 (m, 3H, H18b and O–CH_2_–C**H**_2_), 1.45–1.33 (m, 2H, H19a and H20), 1.25 (m, 1H, H19b), 0.96 (m, 1H, H14b), 0.91 and 0.90 (2*d*, ^3^*J* = 6.6 Hz, 3H, CH_3_) ppm. ^13^C{^1^H} NMR (126 MHz, DMSO-*d*_6_): δ 172.2 (COO), 136.1 (C13), 135.8 (C2), 126.7 (C8), 120.2 (C11), 118.2 (C10), 117.4 (C9), 111.1 (C12), 106.2 (C7), 66.7 (C17), 62.0 (O–CH_2_), 61.2 (C21), 60.1 (C3), 52.5 (C5), 52.1 (C16), 40.0 (C20), 36.9 (O–CH_2_–**C**H_2_), 35.8 (C15), 34.0 (C14), 32.5 (C18), 24.7 (**C**H–CH_3_), 23.0 (C19), 22.5 and 22.4 (CH_3_), 21.7 (C6) ppm. HRMS (ESI+): *m*/*z* [M + H]^+^ calculated for C_25_H_35_O_3_N_2_ = 411.2642, found: 411.2641.

#### 4.1.10 17-α-Hydroxyyohimban-16-α-carboxylic Acid Cyclopentyl Ester (**3d**)

Following method A, treatment of **2** with cyclopentanol (3.0 mL) for 48h afforded **3d** (66 mg, 55%) as a white solid. ^1^H NMR (500 MHz, DMSO-*d*_6_): δ 10.78 (bs, 1H, H1), 7.32 (m, 1H, H9), 7.25 (m, 1H, H12), 6.98 (m, 1H, H11), 6.91 (m, 1H, H10), 5.15 (m, 1H, H1′), 4.57 (d, ^3^*J* = 4.7 Hz, 1H, OH), 4.08 (m, 1H, H17), 3.22 (d, ^3^*J* = 11.3 Hz, 1H, H3), 2.98 (m, 1H, H5a), 2.83 (dd, ^2^*J* = 11.0 Hz, ^3^*J* = 3.1 Hz, 1H, H21), 2.75 (m, 1H, H6a), 2.58 (m, 1H, H6b), 2.50–2.41 (m, 2H, H5b and H14a), 2.18 (dd, *J*_16,15_ = 11.5 Hz, *J*_16,17_ = 2.9 Hz, 1H, H16), 2.11 (t, ^2^*J* = ^3^*J* = 10.5 Hz, 1H, H21b), 1.87–1.50 (m, 11H, H15, H18 and H2′–5′), 1.45–1.31 (m, 2H, H19a and H20), 1.24 (m, 1H, H19b), 0.95 (q, ^2^*J* = ^3^*J* = 11.6 Hz, 1H, H14b) ppm. ^13^C{^1^H} NMR (126 MHz, DMSO-*d*_6_): δ 171.8 (COO), 136.1 (C13), 135.8 (C2), 126.7 (C8), 120.2 (C11), 118.2 (C10), 117.4 (C9), 111.1 (C12), 106.2 (C7), 75.8 (C1′), 66.8 (C17), 61.2 (C21), 60.1 (C3), 52.6 (C5), 51.9 (C16), 40.0 (C20), 35.8 (C15), 33.9 (C14), 32.6 (C18), 32.4 and 32.3 (C2′ and C5′), 23.5 and 23.4 (C3′ and C4′), 22.9 (C19), 21.7 (C6) ppm. HRMS (ESI+): *m*/*z* [M + H]^+^ calculated for C_25_H_33_O_3_N_2_ = 409.2486, found: 409.2485.

#### 4.1.11 17-α-Hydroxyyohimban-16-α-carboxylic Acid *tert*-Butyl Ester (**3e**)

*N*,*N*-Dimethylformamide di-*tert*-butyl acetal (0.5 mL, 2.08 mmol) was added at RT to the suspension of **2** (100 mg, 0.29 mmol) in toluene (3.0 mL). The reaction mixture was heated to 90 °C overnight. The reaction mixture was evaporated, and the residue was codistilled with toluene (5.0 mL). The residue was adsorbed on C18 and applied on a flash chromatography column (RP-C18aq, eluent water/CH_3_CN, gradient 0–100%) to give **3e** (41 mg, 35%) as a yellowish solid. ^1^H NMR (500 MHz, CDCl_3_): δ 7.89 (bs, 1H, H1), 7.45 (d, ^3^*J* = 7.8 Hz, 1H, H9), 7.33 (d, ^3^*J* = 8.0 Hz, 1H, H12), 7.14 (m, 1H, H11), 7.08 (m, 1H, H10), 4.19 (bs, 1H, H17), 3.42 (m, 1H, H3), 3.14 (m, 1H, H5a), 3.09–2.95 (m, 2H, H6a and H21a), 2.76–2.64 (m, 2H, H5b and H6b), 2.29 (m, 1H, H21b), 2.18 (d, *J*_16,15_ = 11.6 Hz, 1H, H16), 2.06 (m, 1H, H14a), 1.99–1.92 (m, 2H, H18a and H15), 1.53 (s, 9H, CH_3_), 1.63–1.35 (m, 4H, H14b, H18b and H19) ppm. ^13^C{^1^H} NMR (126 MHz, CDCl_3_): δ 174.6 (COO), 136.0 (C13), 127.2 (C8), 121.6 (C11), 119.5 (C10), 118.2 (C9), 110.9 (C12), 108.1 (C7), 81.8 (**C**–CH_3_), 66.9 (C17), 61.0 (C21), 59.9 (C3), 52.8 (C16), 36.4 (C15), 33.7 (C14), 31.3 (C18), 28.3 (CH_3_), 23.2 (C19), 21.3 (C6) ppm. HRMS (ESI+): *m*/*z* [M + H]^+^ calculated for C_24_H_33_O_3_N_2_ = 397.2486, found: 397.2485.

#### 4.1.12 17-α-Hydroxyyohimban-16-α-carboxylic acid (*R*)-Pyrrolidin-2-ylmethyl Ester (**4a**)

Following method B, treatment of **2** with *N*-Boc-d-prolinol (154 mg, 0.76 mmol) afforded **4a** (81 mg, 32%) as a white solid. ^1^H NMR (600 MHz, D_2_O): δ 7.49 (m, 1H, H9), 7.40 (m, 1H, H12), 7.20 (m, 1H, H11), 7.10 (m, 1H, H10), 4.44 (dd, 1H, ^2^*J* = 12.4, ^3^*J* = 3.5 Hz, H3′a), 4.30 (m, 1H, H17), 4.26 (dd, 1H, ^2^*J* = 12.4, ^2^*J* = 3.5 Hz, H3′b), 3.90 (qd, 1H, ^3^*J* = 7.9 Hz, ^3^*J* = 3.5 Hz, H4′), 3.49–3.37 (m, 1H, H3), 3.24–3.17 (m, 1H, H21a), 3.04–2.76 (m, 3H, H21b, H6), 2.53–2.40 (m, 2H, H14a, H16), 2.21–2.13 (m, 1H, H8′a), 2.07–1.82 (m, 4H, H18a, H15, H7′), 1.75 (m, 1H, H8′b), 1.67–1.59 (m, 1H, H18b), 1.59–1.47 (m, 1H), 1.39 (m, 1H, H19a), 1.36–1.19 (m, 2H, H19b, H14b) ppm. ^13^C{^1^H} NMR (151 MHz, D_2_O): δ 182.7 (C1′), 145.7 (C13), 139.4 (C2), 135.0 (C8), 131.9 (C11), 129.2 (C10), 127.7 (C9), 121.1 (C12), 115.1 (C7), 76.7 (C17), 72.6 (C3′), 67.6 (C4′), 67.5 (C21), 60.1 (C16), 55.3 (C6′), 43.4 (C15), 40.8 (C14), 40.4 (C18), 35.4 (C8′), 32.7 (C7′), 32.5 (C20), 31.1 (C19), 28.2 (C6) ppm. HRMS (ESI+): *m*/*z* [M + H]^+^ calculated for C_25_H_34_O_3_N_3_ = 424.25947, found: 424.25953.

#### 4.1.13 17-α-Hydroxyyohimban-16-α-carboxylic acid (*S*)-Pyrrolidin-2-ylmethyl Ester (**4b**)

Following method B, the treatment of **2** with *N*-Boc-l-prolinol (114 mg, 0.57 mmol) afforded **4b** (63 mg, 54%) as a white solid. ^1^H NMR (600 MHz, D_2_O): δ 7.60 (m, 1H, H9), 7.51 (m, 1H, H12), 7.30 (m, 1H, H11), 7.21 (m, 1H, H10), 4.55 (dd, 1H, ^2^*J* = 12.4, ^3^*J* = 3.6 Hz, H3′a), 4.42 (m, 1H, H17), 4.36 (dd, 1H, ^2^*J* = 12.4, ^2^*J* = 8.0 Hz, H3′b), 4.02 (qd, 1H, ^3^*J* = 8.1 Hz, ^3^*J* = 3.6 Hz, H4′), 3.57 (m, 1H, H3), 3.41–3.29 (m, 4H, H21, H6′), 3.16–3.05 (m, 2H, H6a), 2.96 (m, 1H, H6b), 2.72 (bs, 1H, H5′), 2.69–2.54 (m, 2H, H14a, H16), 2.27 (m, 1H, H8′a), 2.17–2.00 (m, 3H, H15, H20), 1.97 (m, 1H, 18a), 1.92–1.60 (m, 3H, H18b, H8′b), 1.55–1.30 (m, 2H, H19, H14b) ppm. ^13^C{^1^H} NMR (151 MHz, D_2_O): δ 174.2 (C1′), 137.1 (C13), 130.6 (C2), 126.4 (C8), 123.4 (C11), 120.7 (C10), 119.1 (C9), 112.5 (C12), 106.5 (C7), 68.0 (C17), 64.1 (C3′), 59.0 (C4′), 58.8 (C21), 54.4 (C3), 51.5 (C16), 46.6 (C6′), 34.8 (C15), 32.2, 31.8 (C18), 26.8 (C8′), 24.0, 23.9 (C7′), 22.5 (C19), 19.6 (C6) ppm. HRMS (ESI+): *m*/*z* [M + H]^+^ calculated for C_25_H_34_O_3_N_3_ = 424.25947, found: 424.25952.

#### 4.1.14 17-α-Hydroxyyohimban-16-α-carboxylic Acid 2-(Methylamino)ethyl Ester (**4c**)

Following method B, treatment of **2** with *N*-Boc-2-(methylamino)ethanol (84 mg, 0.47 mmol) afforded **4c** (20 mg, 17%) as a yellowish solid. ^1^H NMR (500 MHz, D_2_O): δ 7.62 (m, 1H, H9), 7.53 (m, 1H, H12), 7.32 (m, 1H, H11), 7.22 (m, 1H, H10), 4.56 (dt, 1H, ^2^*J* = 12.9, ^3^*J* = 5.0 Hz, H3′a), 4.49 (m, 1H, H3′b), 4.45 (m, 1H, H17), 4.24 (m, 1H, H3), 3.76 (H5a overlap with intense water signal), 3.54 (m, 1H, H21a), 3.46 (m, 2H, H4′), 3.35 (m, 1H, H5b), 3.19 (m, 1H, H6a), 3.05 (dd, 1H, ^2^*J* = 16.6, ^3^*J* = 5.2 Hz, H6b), 2.98 (m, 1H, H21b), 2.80 (s, 3H, H6′), 2.72 (m, 1H, H14a), 2.66 (dd, 1H, ^3^*J* = 11.7 Hz, ^3^*J* = 2.8 Hz, H16), 2.15 (qd, 1H, ^3^*J* = 11.4 Hz, ^3^*J* = 3.2 Hz, H15), 1.99 (m, 1H, H18a), 1.83–1.79 (m, 2H, H18b, H20), 1.59–1.40 (m, 3H, H14b, H19), 1.37 (m, 1H) ppm. ^13^C{^1^H} NMR (126 MHz, D_2_O): δ 174.1 (C1′), 137.2 (C13), 129.2 (C2), 128.2 (C8), 123.7 (C11), 120.8 (C10), 119.2 (C9), 112.6 (C12), 106.4 (C7), 67.9 (C17), 61.8 (C3), 61.0 (C3′), 58.5 (C21), 53.5 (C5), 51.4 (C16), 48.2 (C4′), 38.3 (C20), 34.5 (C15), 33.7 (C6′), 31.9 (C14), 31.7 (C18), 22.4 (C19), 19.2 (C6) ppm. HRMS (ESI+): *m*/*z* [M + H]^+^ calculated for C_23_H_32_O_3_N_3_ = 398.24382, found: 398.24405.

#### 4.1.15 17-α-Hydroxyyohimban-16-α-carboxylic Acid Piperidine-4-yl Ester (**4d**)

Following method B, treatment of **2** with *N*-Boc-4-hydroxypiperidine (100 mg, 0.5 mmol) afforded **4d** (74 mg, 35%) as a white solid. ^1^H NMR (500 MHz, D_2_O): δ 7.59 (m, 1H, H9), 7.49 (m, 1H, H12), 7.28 (m, 1H, H11), 7.20 (m, 1H, H10), 5.19 (m, 1H, H3′), 4.41 (m, 1H, H17), 3.84 (bm, 1H), 3.47 (m, 1H, H3), 3.42–3.22 (m, 5H, H21a, H5′), 3.12–2.98 (m, 2H, H6a), 2.92 (m, 1H, H6b), 2.61 (m, 1H, H21b), 2.56–2.44 (m, 2H, H14a, H16), 2.23–2.11 (m, 2H, H4′), 3.46 (m, 2H, H4′a), 2.09–2.02 (m, 2H, H4′b), 2.02–1.92 (m, 2H, H15, H18a), 1.72 (m, 1H, H18b), 1.61 (m, 1H, H20), 1.53–1.35 (m, 2H, H19), 1.33–1.22 (m, 1H, H14b) ppm. ^13^C{^1^H} NMR (126 MHz, D_2_O): δ 174.0 (C1′), 137.1 (C13), 131.6 (C2), 126.6 (C8), 123.2 (C11), 120.6 (C10), 119.1 (C9), 112.5 (C12), 106.7 (C7), 68.3 (C17), 67.2 (C3′), 59.2 (C21), 51.8 (C16), 41.2 (C5′), 35.0 (C15), 32.5 (C14), 32.1 (C18), 27.4, 27.2 (C4′), 22.7 (C19), 19.9 (C6) ppm. HRMS (ESI+): *m*/*z* [M + H]^+^ calculated for C_25_H_34_O_3_N_3_ = 424.25947, found: 424.25961.

#### 4.1.16 17-α-Hydroxyyohimban-16-α-carboxylic Acid Piperidine-3-yl Ester (**4e**)

Following method B, the treatment of **2** with *N*-Boc-3-hydroxypiperidine (100 mg, 0.5 mmol) afforded **4e** (74 mg, 35%) as a white solid. ^1^H NMR (500 MHz, D_2_O): δ 7.60 (m, 1H, H9), 7.50 (m, 1H, H12), 7.30 (m, 1H, H11), 7.21 (m, 1H, H10), 5.21 (m, 1H, H3′), 4.41 (m, 1H, H17), 3.85 (m, 1H, H6′a), 3.52 (m, 1H, H5a), 3.43–3.25 (m, 5H, H21a, H4′, H6′b), 3.15–3.02 (m, 2H, H6a), 2.94 (m, 1H, H6b), 2.66 (m, 1H, H21b), 2.60–2.44 (m, 2H, H16, H14a), 2.24–2.11 (m, 2H, H8′a), 2.11–1.92 (m, 4H, H,15, H18a, H8′b), 1.72 (m, 1H, H18b), 1.62 (m, 1H, H20), 1.54–1.27 (m, 2H, H19, H14b) ppm. ^13^C{^1^H} NMR (126 MHz, D_2_O): δ 173.9 (C1′), 137.1 (C13), 131.1 (C2), 126.5 (C8), 123.3 (C11), 120.6 (C10), 119.1 (C9), 112.5 (C12), 106.6 (C7), 68.3 (C17), 67.3 (C3′), 59.0 (C21), 51.7 (C16), 41.2 (C5′), 34.9 (C15), 32.3 (C14), 32.0 (C18), 27.4, (C7′), 27.1 (C8′), 22.6 (C19), 19.7 (C6) ppm. HRMS (ESI+): *m*/*z* [M + H]^+^ calculated for C_25_H_34_O_3_N_3_ = 424.25947, found: 424.25964.

#### 4.1.17 17-α-Hydroxyyohimban-16-α-carboxylic Acid Azetidin-3-ylmethyl Ester (**4f**)

Following method B, treatment of **2** with *N*-Boc-azetidin-3-ylmethanol (165 mg, 0.88 mmol) afforded **4f** (134 mg, 56%) as a yellowish solid. ^1^H NMR (500 MHz, DMSO-*d*_6_): δ 11.30 (bs, 1H, ^1^N–H), 10.12 (bs, 1H), 8.82 (bs, 2H), 7.47 (d, 1H, ^3^*J* = 7.8 Hz, H9), 7.35 (d, 1H, ^3^*J* = 8.1 Hz, H12), 7.16–7.09 (m, 1H, H11), 7.07–7.00 (m, 1H, H10), 4.97 (bs, 1H, OH), 4.80 (d, 1H, ^3^*J* = 12.2 Hz, H3), 4.34–4.27 (m, 1H, H3′a), 4.26–4.20 (m, 1H, H17), 4.16 (dd, 1H, ^2^*J* = 11.5 Hz, ^3^*J* = 5.3 Hz, H3′b), 4.07–3.97 (m, 2H, H5′/H7′), 3.91–3.80 (m, 2H, H5′/H7′), 3.78–3.71 (m, 1H, H5a), 3.57–3.38 (m, 2H, H5b, H21a,), 3.22–3.04 (m, 3H, H6a, H21b, H4′), 3.02–2.94 (m, 1H, H6b), 2.84–2.74 (m, 1H, H14a), 2.48–2.42 (1H, H16), 2.19 (qd, 1H, ^3^*J* = 11.3 Hz, ^3^*J* = 3.1 Hz, H15), 1.85–1.78 (m, 1H, H18a), 1.70–1.53 (m, 2H, H18b, H20), 1.51–1.40 (1H, H14a), 1.40–1.30 (m, 2H, H14b, H19) ppm. ^13^C{^1^H} NMR (126 MHz, DMSO-*d*_6_): δ 171.9 (C1′), 136.6 (C13), 129.3 (C2), 125.8 (C8), 122.1 (C11), 119.4 (C10), 118.3 (C9), 111.6 (C12), 105.5 (C7), 66.5 (C17), 63.4 (C3′), 60.6 (C3), 57.3 (C21), 52.1 (C5), 51.0 (C16), 47.9, 47.8 (C5′, C7′), 37.6 (C20), 33.8 (C15), 32.0 (C18), 31.3 (C14), 30.8 (C4′), 22.0 (C19), 18.9 (C6) ppm. HRMS (ESI+): *m*/*z* [M + H]^+^ calculated for C_24_H_32_O_3_N_3_ = 410.24382, found: 410.24372.

#### 4.1.18. 17-α-Hydroxyyohimban-16-α-carboxylic Acid (*R*)-Azetidin-2-ylmethyl Ester (**4g**)

4.1.18

Following method B, treatment of **2** with *N*-Boc-(*R*)-azetidin-3-ylmethanol (107 mg, 0.57 mmol) afforded **4g** (37 mg, 21%) as a yellowish solid. ^1^H NMR (500 MHz, DMSO-*d*_6_): δ 11.32 (bs, 1H, ^1^N–H), 10.29 (bs, 1H), 9.24 (bs, 1H), 9.10 (bs, 1H), 7.47 (d, 1H, ^3^*J* = 7.9 Hz, H9), 7.35 (d, 1H, ^3^*J* = 8.1 Hz, H12), 7.16–7.09 (m, 1H, H11), 7.06–7.00 (m, 1H, H10), 4.98 (bs, 1H, OH), 4.89–4.75 (m, 1H, H3), 4.74–4.63 (m, 1H, H4′), 4.47 (dd, 1H, ^2^*J* = 12.5 Hz, ^3^*J* = 5.9 Hz, H3′a), 4.32–4.24 (m, 2H, H17, H3′b), 4.00–3.81 (m, 2H, H6′), 3.80–3.71 (m, 1H, H5a), 3.53–3.44 (m, 2H, H5b, H21a,), 3.22–3.03 (m, 2H, H6a, H21b), 2.98 (dd, 1H, ^2^*J* = 16.2 Hz, ^3^*J* = 5.1 Hz, H6b), 2.82 (dm, 1H, H14a), 2.49–2.37 (3H, H16, H7′), 2.21 (qd, 1H, ^3^*J* = 11.3 Hz, ^3^*J* = 3.0 Hz, H15), 1.87–1.80 (m, 1H, H18a), 1.74–1.55 (m, 2H, H18b, H20), 1.51–1.32 (3H, H14b, H19) ppm. ^13^C{^1^H} NMR (126 MHz, DMSO-*d*_6_): δ 171.3 (C1′), 136.5 (C13), 129.3 (C2), 125.7 (C8), 122.0 (C11), 119.3 (C10), 118.1 (C9), 111.6 (C12), 105.4 (C7), 66.2 (C17), 62.8 (C3′), 60.4 (C3), 57.8 (C4′), 57.2 (C21), 51.9 (C5), 51.1 (C16), 42.7 (C6′), 37.5 (C20), 33.8 (C15), 31.9 (C18), 31.2 (C14), 22.0 (C19), 20.8 (C7′), 18.7 (C6) ppm. HRMS (ESI+): *m*/*z* [M + H]^+^ calculated for C_24_H_32_O_3_N_3_ = 410.24382, found: 410.24348.

#### 4.1.19 17-α-Hydroxyyohimban-16-α-carboxylic acid (*S*)-Azetidin-2-ylmethyl Ester (**4h**)

Following method B, treatment of **2** with *N*-Boc-(*S*)-azetidin-3-ylmethanol (107 mg, 0.57 mmol) afforded **4h** (30 mg, 17%) as a yellowish solid. ^1^H NMR (500 MHz, DMSO-*d*_6_): δ 11.31 (bs, 1H, ^1^N–H), 10.24 (bs, 1H), 9.26 (bs, 1H), 9.06 (bs, 1H), 7.47 (d, 1H, ^3^*J* = 7.8 Hz, H9), 7.36 (d, 1H, ^3^*J* = 8.1 Hz, H12), 7.16–7.09 (m, 1H, H11), 7.07–7.00 (m, 1H, H10), 4.96 (bs, 1H, OH), 4.88–4.74 (m, 1H, H3), 4.70–4.59 (m, 1H, H4′), 4.46–4.36 (m, 2H, H3′), 4.29–4.21 (m, 1H, H17), 4.00–3.81 (m, 2H, H6′), 3.75 (dd, 1H, ^2^*J* = 12.1 Hz, ^3^*J* = 6.0 Hz, H5a), 3.53–3.41 (m, 2H, H5b, H21a,), 3.20–3.06 (m, 2H, H6a, H21b), 2.98 (dd, 1H, ^2^*J* = 16.2 Hz, ^3^*J* = 5.2 Hz, H6b), 2.82 (dm, 1H, H14a), 2.49 (H7′a), 2.48 (dd, 1H, ^3^*J* = 11.4 Hz, ^3^*J* = 3.1 Hz, H16), 2.43–2.31 (m, 1H, H7′b), 2.20 (qd, 1H, ^3^*J* = 11.4 Hz, ^3^*J* = 3.1 Hz, H15), 1.86–1.77 (m, 1H, H18a), 1.74–1.55 (m, 2H, H18b, H20), 1.54–1.30 (3H, H14b, H19) ppm. ^13^C{^1^H} NMR (126 MHz, DMSO-*d*_6_): δ 171.5 (C1′), 136.5 (C13), 129.3 (C2), 125.7 (C8), 122.0 (C11), 119.3 (C10), 118.2 (C9), 111.6 (C12), 105.4 (C7), 66.1 (C17), 63.0 (C3′), 60.4 (C3), 57.8 (C4′), 57.1 (C21), 51.9 (C5), 51.1 (C16), 42.9 (C6′), 37.5 (C20), 33.7 (C15), 31.8 (C18), 31.2 (C14), 22.0 (C19), 20.9 (C7′), 18.8 (C6) ppm. HRMS (ESI+): *m*/*z* [M + H]^+^ calculated for C_24_H_32_O_3_N_3_ = 410.24382, found: 410.24375.

#### 4.1.20. 17-α-Hydroxyyohimban-16-α-carboxylic Acid (*R*)-Pyrrolidin-3-ylmethyl Ester (**4i**)

4.1.20

Following method B, treatment of **2** with *N*-Boc-(*R*)-pyrrolidin-3-ylmethanol (115 mg, 0.57 mmol) afforded **4i** (53 mg, 28%) as a yellowish solid. ^1^H NMR (500 MHz, DMSO-*d*_6_): δ 11.32 (bs, 1H, ^1^N–H), 10.18 (bs, 1H, ^4^N–H^+^), 9.06 (bs, 1H), 9.02 (bs, 1H), 7.47 (d, 1H, ^3^*J* = 7.9 Hz, H9), 7.35 (d, 1H, ^3^*J* = 8.2 Hz, H12), 7.16–7.09 (m, 1H, H11), 7.06–7.00 (m, 1H, H10), 4.91 (bs, 1H, OH), 4.86–4.76 (m, 1H, H3), 4.24–4.16 (m, 2H, H17, H3′a), 4.05 (dd, 1H, ^2^*J* = 11.0 Hz, ^3^*J* = 6.7 Hz, H3′b), 3.79–3.71 (m, 1H, H5a), 3.53–3.31 (m, 3H, H5b, H21a, H5′a), 3.30–3.22 (m, 1H, H7′a), 3.22–3.04 (m, 3H, H6a, H21b, H7′b), 2.79 (dm, 1H, H14a), 2.70–2.59 (m, 1H, H4′), 2.42 (dd, 1H, ^3^*J* = 11.5 Hz, ^3^*J* = 2.8 Hz), 2.15 (qm, 1H, H15), 2.11–2.00 (m, 1H, 8′a), 1.85–1.78 (m, 1H, H18a), 1.77–1.69 (m, 1H, H8′b), 1.70–1.54 (m, 2H, H18b, H20), 1.52–1.29 (3H, H14b, H19) ppm. ^13^C{^1^H} NMR (126 MHz, DMSO-*d*_6_): δ 171.6 (C1′), 136.5 (C13), 129.2 (C2), 125.7 (C8), 122.0 (C11), 119.3 (C10), 118.1 (C9), 111.6 (C12), 106.4 (C7), 66.3 (C17), 64.1 (C3′), 60.4 (C3), 57.1 (C21), 51.9 (C5), 50.9 (C16), 46.7 (C5′), 44.6 (C7′), 37.5 (C20), 36.5 (C4′), 33.7 (C15), 31.9 (C18), 31.2 (C14), 26.7 (C8′), 21.9 (C19), 18.7 (C6) ppm. HRMS (ESI+): *m*/*z* [M + H]^+^ calculated for C_25_H_34_O_3_N_3_ = 424.25947, found: 424.25932.

#### 4.1.21 17-α-Hydroxyyohimban-16-α-carboxylic Acid (*S*)-Pyrrolidin-3-ylmethyl Ester (**4j**)

Following method B, treatment of **2** with *N*-Boc-(*S*)-pyrrolidin-3-ylmethanol (115 mg, 0.57 mmol) afforded **4j** (35 mg, 19%) as a yellowish solid. ^1^H NMR (500 MHz, DMSO-*d*_6_): δ 11.32 (bs, 1H, ^1^N–H), 10.20 (bs, 1H), 9.06 (bs, 2H), 7.47 (d, 1H, ^3^*J* = 7.9 Hz, H9), 7.35 (d, 1H, ^3^*J* = 8.2 Hz, H12), 7.16–7.09 (m, 1H, H11), 7.07–7.00 (m, 1H, H10), 4.92 (bs, 1H, OH), 4.86–4.72 (m, 1H, H3), 4.22–4.13 (m, 2H, H17, H3′a), 4.09 (dd, 1H, ^2^*J* = 11.0 Hz, ^3^*J* = 6.0 Hz, H3′b), 3.80–3.69 (m, 1H, H5a), 3.52–3.31 (m, 3H, H5b, H21a, H5′a), 3.31–3.23 (m, 1H, H7′a), 3.22–3.04 (m, 3H, H6a, H21b, H7′b), 3.02–2.93 (m, 2H (6b, 5′b), 2.79 (dm, 1H, H14a), 2.70–2.59 (m, 1H, H4′), 2.42 (dd, 1H, ^3^*J* = 11.5 Hz, ^3^*J* = 2.8 Hz), 2.17 (qm, 1H, H15), 2.12–2.01 (m, 1H, 8′a), 1.85–1.78 (m, 1H, H18a), 1.78–1.68 (m, 1H, H8′b), 1.68–1.55 (m, 2H, H18b, H20), 1.53–1.29 (3H, H14b, H19) ppm. ^13^C{^1^H} NMR (126 MHz, DMSO-*d*_6_): δ 171.6 (C1′), 136.5 (C13), 129.2 (C2), 125.7 (C8), 122.0 (C11), 119.3 (C10), 118.1 (C9), 111.6 (C12), 105.4 (C7), 66.4 (C17), 64.2 (C3′), 60.4 (C3), 57.1 (C21), 51.9 (C5), 50.9 (C16), 46.9 (C5′), 44.6 (C7′), 37.5 (C20), 36.5 (C4′), 33.7 (C15), 31.9 (C18), 31.2 (C14), 26.7 (C8′), 21.9 (C19), 18.8 (C6) ppm. HRMS (ESI+): *m*/*z* [M + H]^+^ calculated for C_25_H_34_O_3_N_3_ = 424.25947, found: 424.25931.

#### 4.1.22 17-α-Hydroxyyohimban-16-α-carboxylic Acid (*R*)-Pyrrolidin-3-yl Ester (**4k**)

Following method B, treatment of **2** with *N*-Boc-(*R*)-pyrrolidin-3-ol (107 mg, 0.57 mmol) afforded **4k** (35 mg, 19%) as a yellowish solid. ^1^H NMR (500 MHz, DMSO-*d*_6_): δ 11.31 (bs, 1H, ^1^N–H), 10.28 (bs, 1H, ^4^N–H^+^), 9.43 (bs, 1H), 9.26 (bs, 1H), 7.47 (d, 1H, ^3^*J* = 7.8 Hz, H9), 7.36 (d, 1H, ^3^*J* = 8.1 Hz, H12), 7.15–7.10 (m, 1H, H11), 7.06–7.01 (m, 1H, H10), 5.44–5.40 (m, 1H, H3′), 4.95 (bs, 1H, OH), 4.85–4.75 (m, 1H, H3), 4.29–4.21 (m, 1H, H17), 3.79–3.70 (m, 1H, H5a), 3.54–3.32 (m, 5H, H5b, H21a, H4′a, H6′), 3.32–3.21 (m, 1H, H4′b), 3.15–3.05 (m, 2H, H6a, H21b), 3.01–2.94 (m, 1H, H6b), 2.82–2.75 (m, 1H, H14a), 2.55 (bs, 1H, NH), 2.40–2.35 (m, 1H, H16), 2.27–2.06 (m, 3H, H7′, H15), 1.81–1.75 (m, 1H, H18a), 1.72–1.52 (m, 1H, H20), 1.62–1.51 (m, 1H, 18b), 1.50–1.29 (m, 3H, H19, H14b) ppm. ^13^C{^1^H} NMR (126 MHz, DMSO-*d*_6_): 171.2 (C1′), 136.5(C13), 129.3 (C2), 125.7 (C8), 122.0 (C11), 119.3 (C10), 118.1 (C9), 111.6 (C12), 105.4 (C7), 72.5 (C3′), 66.0 (C17), 60.4 (C3), 57.2 (C21), 52.0 (C5), 51.0 (C16), 50.1 (C6′), 43.6 (C4′), 37.4 (C20), 33.7 (C15), 31.9 (C18), 31.1 (C14), 30.6 (C7′), 22.0 (C19), 18.8 (C6) ppm.HRMS (ESI+): *m*/*z* [M + H]^+^ calculated for C_24_H_32_O_3_N_3_ = 410.24382, found: 410.24345.

#### 4.1.23 17-α-Hydroxyyohimban-16-α-carboxylic Acid (*S*)-Pyrrolidin-3-yl Ester (**4l**)

Following method B, treatment of **2** with *N*-Boc-(*S*)-pyrrolidin-3-ol (107 mg, 0.57 mmol) afforded **4l** (20 mg, 11%) as a yellowish solid. ^1^H NMR (500 MHz, DMSO-*d*_6_): δ 11.33 (bs, 1H, ^1^N–H), 10.17 (bq, 1H, ^4^N–H^+^), 9.85 (bs, 1H), 9.31 (bs, 1H), 8.89 (s, 1H), 7.47 (d, 1H, ^3^*J* = 7.9 Hz, H9), 7.35 (dm, 1H, H12), 7.15–7.10 (m, 1H, H11), 7.06–7.01 (m, 1H, H10), 5.39–5.34 (m, 1H, H3′), 4.94 (bs, 1H, OH), 4.86–4.72 (m, 1H, H3), 4.24–4.18 (m, 1H, H17), 3.75 (dd, 1H, ^2^*J* = 12.1 Hz, ^3^*J* = 6.0 Hz, H5a), 3.53–3.31 (m, 5H, H5b, H21a, H4′a, H6′), 3.29–3.20 (m, 1H, H4′b), 3.18–3.06 (m, 2H, H6a, H21b), 2.99 (dd, 1H, ^2^*J* = 16.2 Hz, ^3^*J* = 5.4 Hz, H6b), 2.50 (dm, 1H, H14a), 2.36 (dd, 1H, ^3^*J* = 11.6 Hz, ^3^*J* = 2.9 Hz), 2.22–2.07 (m, 3H, H15, H7′), 1.85–1.77 (m, 1H, H18a), 1.71–1.52 (m, 2H, H18b, H20), 1.50–1.29 (3H, H14b, H19) ppm. ^13^C{^1^H} NMR (126 MHz, DMSO-*d*_6_): δ 171.1 (C1′), 136.5 (C13), 129.2 (C2), 125.7 (C8), 122.0 (C11), 119.3 (C10), 118.2 (C9), 111.6 (C12), 105.4 (C7), 72.7 (C3′), 66.1 (C17), 60.4 (C3), 57.1 (C21), 51.9 (C5), 50.7 (C16), 50.1 (C6′), 43.5 (C4′), 37.4 (C20), 33.6 (C15), 31.9 (C18), 31.1 (C14), 30.0 (C7′), 21.9 (C19), 18.7 (C6) ppm. HRMS (ESI+): *m*/*z* [M + H]^+^ calculated for C_24_H_32_O_3_N_3_ = 410.24382, found: 410.24376.

#### 4.1.24 17-α-Hydroxyyohimban-16-α-carboxylic Acid Azetidin-3-yl Ester (**4m**)

Following method C, treatment of **2** with *N*-Boc-azetidin-3-ol (50 mg, 0.29 mmol) afforded **4m** (62 mg, 54%) as a yellowish solid. ^1^H NMR (500 MHz, DMSO-*d*_6_): δ 11.31 (bs, 1H, ^1^N–H), 10.25 (bs, 1H, ^4^N–H^+^), 9.32 (bs, 1H, ^5^′NH–H^+^), 9.14 (bs, 1H, ^5^′NH–H^+^), 7.47 (d, 1H, ^3^*J* = 7.9 Hz, H9), 7.36 (d, 1H, ^3^*J* = 8.1 Hz, H12), 7.15–7.10 (m, 1H, H11), 7.06–7.01 (m, 1H, H10), 5.30 (pentet, 1H, ^3^*J* = 6.2 Hz, H3′), 5.03 (bs, 1H, OH), 4.85–4.76 (m, 1H, H3), 4.41–4.29 (m, 2H, H4′/H6′), 4.27–4.22 (m, 1H, H17), 4.10–4.01 (m, 2H, H4′/H6′), 3.75 (dd, 1H, ^2^*J* = 12.1 Hz, ^3^*J* = 6.0 Hz, H5a), 3.52–3.38 (m, 2H, H5b, H21a), 3.19–3.03 (m, 2H, H6a, H21b), 2.98 (dd, 1H, ^2^*J* = 16.2 Hz, ^3^*J* = 5.1 Hz, H6b), 2.79–2.73 (dm, 1H, H14a), 2.45 (d, 1H, ^3^*J* = 2.7 Hz, H16), 2.17 (qd, 1H, ^3^*J* = 11.4 Hz, ^3^*J* = 3.0 Hz, H15), 1.87–1.79 (m, 1H, H18a), 1.73–1.55 (m, 2H, H18b, H20), 1.51–1.30 (m, 3H, H14b, H19) ppm. ^13^C{^1^H} NMR (126 MHz, DMSO-*d*_6_): δ 171.1 (C1′), 136.5 (C13), 129.2 (C2), 125.7 (C8), 122.0 (C11), 119.3 (C10), 118.1 (C9), 111.6 (C12), 105.4 (C7), 66.1 (C17), 64.5 (C3′), 60.3 (C3), 57.1 (C21), 52.2, 52.1 (C4′, C6′), 51.9 (C5), 50.7 (C16), 37.3 (C20), 33.6 (C15), 31.2 (C18), 31.0 (C14), 21.9 (C19), 18.7 (C6) ppm. HRMS (ESI+): *m*/*z* [M + H]^+^ calculated for C_24_H_32_O_3_N_3_ = 396.22817, found: 396.22795.

#### 4.1.25 17-α-Hydroxyyohimban-16-α-carboxylic Acid *trans*-3-Aminocyclobutyl Ester (**4n**)

Following method C, treatment of **2** with *cis*-*N*-Boc-3-aminocyclobutanol (75 mg, 0.40 mmol) afforded **4n** (115 mg, 70%) as a yellowish solid. ^1^H NMR (500 MHz, DMSO-*d*_6_): δ 11.33 (bs, 1H, ^1^N–H), 10.24 (bq, 1H, ^4^N–H^+^), 8.29 (bs, 2H, NH_2_), 7.47 (d, 1H, ^3^*J* = 7.8 Hz, H9), 7.35 (d, 1H, ^3^*J* = 8.1 Hz, H12), 7.16–7.09 (m, 1H, H11), 7.07–6.99 (m, 1H, H10), 5.26–5.18 (m, 1H, H3′), 4.92 (bs, 1H, OH), 4.85–4.74 (m, 1H, H3), 4.25–4.16 (m, 1H, H17), 3.91–3.79 (m, 1H, H5′), 3.78–3.69 (m, 1H, H5a), 3.52–3.41 (m, 2H, H5b, H21a), 3.19–3.04 (m, 2H, H6a, H21b), 2.98 (dd, 1H, ^2^*J* = 16.2 Hz, ^3^*J* = 5.3 Hz, H6b), 2.82–2.74 (m, 1H, H14a), 2.59–2.42 (m, 4H, H4′, H6′), 2.39 (dd, 1H, ^3^*J* = 11.5 Hz, ^3^*J* = 2.8 Hz, H16), 2.16 (qd, 1H, ^3^*J* = 11.3 Hz, ^3^*J* = 3.2 Hz, H15), 1.85–1.76 (m, 1H, H18a), 1.71–1.54 (m, 2H, H18b, H20), 1.50–1.30 (m, 3H, H14b, H19) ppm. ^13^C{^1^H} NMR (126 MHz, DMSO-*d*_6_): δ 171.2 (C1′), 136.5 (C13), 129.3 (C2), 125.7 (C8), 122.0 (C11), 119.3 (C10), 118.1 (C9), 111.6 (C12), 105.4 (C7), 66.3 (C17), 66.1 (C3′), 60.4 (C3), 57.1 (C21), 51.9 (C5), 50.7 (C16), 41.7 (C5′), 37.4 (C20), 34.0 (C4′, C6′), 33.7 (C15), 32.0 (C18), 31.1 (C14), 21.9 (C19), 18.7 (C6) ppm. HRMS (ESI+): *m*/*z* [M + H]^+^ calculated for C_24_H_32_O_3_N_3_ = 410.24382, found: 410.24378.

#### 4.1.26 17-α-Hydroxyyohimban-16-α-carboxylic Acid *cis*-3-Aminocyclobutyl Ester (**4o**)

Following method C, treatment of **2** with *trans*-*N*-Boc-3-aminocyclobutanol (75 mg, 0.4 mmol) afforded **4o** (87 mg, 53%) as a white solid. ^1^H NMR (500 MHz, DMSO-*d*_6_): δ 11.35 (bs, 1H, ^1^N–H), 10.23 (bq, 1H, ^4^N–H^+^), 8.24 (bs, 2H, NH_2_), 7.47 (d, 1H, ^3^*J* = 7.8 Hz, H9), 7.35 (d, 1H, ^3^*J* = 8.1 Hz, H12), 7.15–7.10 (m, 1H, H11), 7.06–7.01 (m, 1H, H10), 4.85–4.76 (m, 2H, H3, H3′), 4.22–4.17 (m, 1H, H17), 3.74 (dd, 1H, ^2^*J* = 12.2 Hz, ^3^*J* = 6.1 Hz, H5a), 3.51–3.40 (m, 3H, H5b, H21a, H5′), 3.18–3.05 (m, 2H, H6a, H21b), 2.98 (dd, 1H, ^2^*J* = 16.2 Hz, ^3^*J* = 5.2 Hz, H6b), 2.82–2.74 (m, 1H, H14a), 2.74–2.62 (m, 2H, H4′/H6′), 2.37 (dd, 1H, ^3^*J* = 11.5 Hz, ^3^*J* = 2.8 Hz, H16), 2.30–2.20 (m, 2H, H4′/H6′), 2.15 (qd, 1H, ^3^*J* = 11.3 Hz, ^3^*J* = 3.1 Hz, H15), 1.85–1.76 (m, 1H, H18a), 1.72–1.55 (m, 2H, H18b, H20), 1.51–1.29 (m, 3H, H14b, H19) ppm. ^13^C{^1^H} NMR (126 MHz, DMSO-*d*_6_): δ 170.5 (C1′), 136.5 (C13), 129.2 (C2), 125.7 (C8), 121.5 (C11), 119.3 (C10), 118.1 (C9), 111.6 (C12), 105.4 (C7), 66.2 (C17), 61.6 (C3′), 60.4 (C3), 57.1 (C21), 51.5 (C5), 50.6 (C16), 37.7 (C5′), 37.4 (C20), 35.4, 35.3 (C4′, C6′), 33.6 (C15), 31.9 (C18), 31.1 (C14), 21.9 (C19), 18.7 (C6) ppm. HRMS (ESI+): *m*/*z* [M + H]^+^ calculated for C_24_H_32_O_3_N_3_ = 410.24382, found: 410.24371.

#### 4.1.27 17-α-Hydroxyyohimban-16-α-carboxylic Acid (*S*)-*N*-Methylpyrrolidin-3-yl Ester (**4p**)

Following method C, treatment of **2** with (*R*)-3-hydroxy-1-methyl-pyrrolidine (36 mg, 0.35 mmol) afforded **4p** (76 mg, 51%) as a white solid. ^1^H NMR (500 MHz, DMSO-*d*_6_): δ 11.32 (bs, 1H, ^1^N–H), 10.76 (bs, 1H), 10.20 (bs, 1H), 7.47 (d, ^3^*J* = 7.9 Hz, 1H, H9), 7.36 (d, ^3^*J* = 8.1 Hz, 1H, H12), 7.17–7.09 (m, 1H, H11), 7.07–6.99 (m, 1H, H10), 5.43–5.29 (m, 1H), 5.09–4.88 (m, 1H), 4.86–4.73 (m, 1H), 4.27–4.19 (m, 1H), 3.82–3.59 (m, 3H), 3.54–3.23 (m, 8H), 3.21–3.05 (m, 5H, H6a), 3.02–2.94 (m, 2H, H6b), 2.89 (s, 3H, NCH_3_), 2.83–2.75 (m, 1H, H14a), 2.44–2.29 (m, 3H), 2.22–2.11 (m, 1H, H15), 1.84–1.85 (m, 1H), 1.73–1.29 (m, 5H, H14b, H18b, H19, H20) ppm. ^13^C{^1^H} NMR (126 MHz, DMSO-*d*_6_): δ 171.1 (C1′), 136.5 (C13), 129.2 (C2), 125.7 (C8), 122.0 (C11), 119.3 (C10), 118.3 (C9), 111.6 (C12), 105.4 (C7), 72.3, 66.1 (C17), 63.1, 60.4 (C3), 59.6, 57.1, 53.3, 51.7, 50.7 (C16), 48.6, 48.4, 44.3, 43.9, 42.6, 37.4 (C20), 36.8, 35.5, 33.6 (C15), 31.9 (C18), 31.1 (C14), 21.9 (C19), 18.7 (C6) ppm. HRMS (ESI+): *m*/*z* [M + H]^+^ calculated for C_25_H_34_O_3_N_3_ = 424.25947, found: 424.25940.

#### 4.1.28 17-α-Hydroxyyohimban-16-α-carboxylic Acid (*S*)-Tetrahydrofuran-3-yl Ester (**4q**)

Following method C, treatment of **2** with (*R*)-tetrahydrofuran-3-ol (31 mg, 0.35 mmol) afforded **4q** (59 mg, 41%) as a white solid. ^1^H NMR (500 MHz, DMSO-*d*_6_): δ 10.26 (bs, 1H, ^1^N–H), 7.33 (d, ^3^*J* = 7.7 Hz, 1H, H9), 7.26 (d, ^3^*J* = 8.0 Hz, 1H, H12), 7.01–6.96 (m, 1H, H11), 6.94–6.89 (m, 1H, H10), 5.31–5.26 (m, 1H, H3′), 4.64 (d, ^3^*J* = 4.8 Hz 1H, OH), 4.13–4.07 (m, 1H, H17), 3.84 (dd, 1H, ^2^*J* = 10.4 Hz, ^3^*J* = 4.6 Hz, H4′a), 3.81–3.71 (m, 3H, H5′, H4′b), 3.27–3.20 (m, 1H, H3), 2.99 (dd, 1H, ^2^*J* = 11.2 Hz, ^3^*J* = 5.6 Hz, H5a), 2.87–2.81 (m, 1H, H21a), 2.80–2.71 (m, 1H, H6a), 2.62–2.55 (m, 1H, H6b), 2.42 (dt, ^3^*J* = 12.2 Hz, ^3^*J* = 3.0 Hz, 1H, H14a), 2.25 (dd, ^3^*J* = 11.5 Hz, ^3^*J* = 2.9 Hz, 1H, H16), 2.18–2.08 (m, 2H, H6′a, H21b), 2.01–1.93 (m, 1H, H6′b), 1.84 (qd, ^3^*J* = 11.2 Hz, ^3^*J* = 3.2 Hz, 1H, H15), 1.84 (dq, *J* = 13.3 Hz, ^3^*J* = 2.9 Hz, 1H, H18a), 1.60–1.50 (m, 1H, H18b), 1.47–1.32 (m, 2H, H19a, H20), 1.29–1.20 (m, 1H, H19b), 0.97 (q, ^3^*J* = 11.7 Hz, 1H, H14b) ppm. ^13^C{^1^H} NMR (126 MHz, DMSO-*d*_6_): δ 171.9 (C1′), 136.0 (C13), 126.7 (C2), 120.2 (C11), 118.9 (C10), 117.4 (C9), 111.1 (C12), 106.2 (C7), 74.2 (C3′), 72.4 (C4′), 66.7 (C17), 66.3 (C5′), 60.5 (C3), 61.2 (C21), 60.1 (C3), 52.6 (C5), 51.8 (C16), 48.6 (C20), 35.7 (C15), 33.9 (C14), 32.5 (C18), 32.2 (C6′), 22.9 (C19), 21.6 (C6) ppm. HRMS (ESI+): *m*/*z* [M + H]^+^ calculated for C_24_H_31_O_4_N_2_ = 411.22783, found: 411.22764.

#### 4.1.29 17-α-Hydroxyyohimban-16-α-carboxylic Acid Oxetan-3-yl Ester (**4r**)

Oxetan-3-ol (53 μL, 0.81 mmol) was added to a solution of yohimbic acid (117 mg, 0.34 mmol), EDC·HCl (113 mg, 0.59 mmol), and DMAP (118 mg, 0.97 mmol) in CH_3_CN (3.0 mL) under argon. The reaction mixture was stirred at RT for 2.5 h, diluted with CHCl_3_ (10 mL), and extracted by aqueous sat. NaHCO_3_ solution. The organic layer was separated and dried over Na_2_SO_4_. Volatiles were evaporated, and the residue was purified on basic alumina (CHCl_3_/MeOH, 0–100%) and subsequently by C18 column RP (H_2_O/CH_3_CN, 0–100%) to obtain **4r** (35 mg, 26%) as a white solid. ^1^H NMR (401 MHz, DMSO-*d*_6_) δ 10.75 (bs, 1H, H1), 7.35–7.30 (m, 1H, H9), 7.30–7.22 (m, 1H, H12), 6.99 (ddd, *J* = 8.1, 7.0, 1.3 Hz, 1H, H11), 6.92 (ddd, *J* = 8.0, 7.1, 1.1 Hz, 1H, H10), 5.43 (tt, *J* = 6.3, 5.1 Hz, 1H, COO–CH), 4.93–4.78 (m, 2H. OCH_2_), 4.73 (d, *J* = 4.7 Hz, 1H, OH), 4.61–4.50 (m, 2H, OCH_2_), 4.26–4.12 (m, 1H, H17), 3.23 (dd, *J* = 11.3, 2.4 Hz, 1H, H3), 3.08–2.93 (m, 1H, H5a), 2.84 (dd, *J* = 11.0, 2.7 Hz, 1H, H21a), 2.79–2.70 (m, 1H, H6a), 2.63–2.54 (m, 1H, H6b), 2.48 (m, 1H, H5b), 2.44–2.37 (m, 1H, H14a), 2.34 (dd, *J* = 11.5, 2.8 Hz, 1H, H16), 2.12 (t, *J* = 10.3 Hz, 1H, H21b), 1.96–1.73 (m, 2H, H15 and H18a), 1.70–1.51 (m, 1H, H18b), 1.48–1.36 (m, 2H, H20 and H19a), 1.33–1.22 (m, 1H, H19b), 0.99 (q, *J* = 11.7 Hz, 1H, H14b) ppm. ^13^C{^1^H} NMR (101 MHz, DMSO-*d*_6_) δ 171.7 (COO), 136.0 (C13), 135.8 (C2), 126.7 (C8), 120.2 (C11), 118.2 (C10), 117.4 (C9), 111.0 (C12), 106.2 (C7), 76.63 and 76.55 (OCH_2_), 67.4 (COO–**C**H), 66.6 (C17), 61.2 (C21), 60.0 (C3), 52.5 (C5), 51.8 (C16), 35.8 (C15), 33.9 (C14), 32.5 (C18), 22.9 (C19), 21.6 (C6). HRMS (ESI+): *m*/*z* [M + H]^+^ calculated for C_23_H_29_O_4_N_2_ = 397.2122, found: 397.2121.

#### 4.1.30 17-α-Hydroxyyohimban-16-α-carboxylic Acid (*S*)-*N*-(Pyrrolidin-3-yl)amide (**5a**)

Following method D, treatment of **2** with 1-(*S*)-*N*-(pyrrolidin-3-yl)amine (118 mg, 0.53 mmol) afforded **5a** (29 mg, 20%) as a white solid. ^1^H NMR (500 MHz, DMSO-*d*_6_): δ 11.33 (bs, 1H, ^1^N–H), 10.37 (bq, 1H, _4_N^–^H^+^), 9.16 (bs, 2H, ^*5′*^N–H, ^*5′*^NH–H^+^), 8.44 (bs, 1H, ^*2′*^N–H), 7.47 (d, ^3^*J* = 7.8 Hz, 1H, H9), 7.34 (d, ^3^*J* = 8.1 Hz, 1H, H12), 7.15–7.09 (m, 1H, H11), 7.06–7.00 (m, 1H, H10), 4.82–4.72 (m, 1H, H3), 4.65 (bs, 1H, OH), 4.46–4.37 (m, 1H, H3′), 4.08–4.03 (m, 1H, H17), 3.34 (dd, 1H, ^2^*J* = 9.5 Hz, ^3^*J* = 4.7 Hz, H5a), 3.53–3.38 (m, 4H, H21a, H5b, H4′a), 3.35–3.21 (m, 2H, H6′), 3.16–3.06 (m, 3H, H6a, H21b, H4′b), 2.97 (dd, 1H, ^2^*J* = 16.2 Hz, ^3^*J* = 5.0 Hz, H6b), 2.63–2.56 (m, 1H, H14a), 2.52–2.12 (m, 2H, H15, H7′a), 2.09 (dd, ^3^*J* = 11.5 Hz, ^3^*J* = 2.2 Hz, 1H, H16), 1.83–1.76 (m, 1H, H18a), 1.72–1.60 (m, 1H, H20), 1.53–1.42 (m, 2H, H19a, H18b), 1.42–1.32 (m, 2H, H14b, H19b) ppm. ^13^C{^1^H} NMR (126 MHz, DMSO-*d*_6_): δ 172.6 (C1′), 136.4 (C13), 129.3 (C2), 125.7 (C8), 121.9 (C11), 119.2 (C10), 118.2 (C9), 111.5 (C12), 105.4 (C7), 66.4 (C17), 60.5 (C3), 57.3 (C21), 51.9 (C5), 51.4 (C16), 49.6 (C4′), 48.2 (C3′), 43.8 (C6′), 37.9 (C20), 33.9 (C15), 31.7 (C18), 31.0 (C14), 30.0 (C7′), 22.2 (C19), 18.7 (C6) ppm. HRMS (ESI+): *m*/*z* [M + H]^+^ calculated for C_24_H_33_O_2_N_4_ = 409.25980, found: 409.25966.

#### 4.1.31 17-α-Hydroxyyohimban-16-α-carboxylic Acid *N*-(Azetidin-3-yl)amide (**5b**)

Following method D, treatment of **2** with 1-*N*-Boc-azetidin-3-yl-amine (110 mg, 0.53 mmol) afforded **5b** (47 mg, 34%) as a white solid. ^1^H NMR (500 MHz, DMSO-*d*_6_): δ 11.30 (bs, 1H, ^1^N–H), 10.35 (bd, 1H, ^21^N–H^+^), 9.11, 8.95 (bs, 2H, ^*2′*^N–H^+^, ^*5′*^N–H^+^), 8.84 (d, ^3^*J* = 7.0 Hz, 1H, H2′), 7.47 (d, ^3^*J* = 7.9 Hz, 1H, H9), 7.34 (d, ^3^*J* = 8.1 Hz, 1H, H12), 7.15–7.10 (m, 1H, H11), 7.06–7.00 (m, 1H, H10), 4.80–4.66 (m, 2H, H3, H3′), 4.22–4.11 (m, 2H, H4′), 4.09–4.05 (m, 1H, H17), 4.05–3.97 (m, 2H, H4′), 3.75 (dd, 1H, ^2^*J* = 12.2 Hz, ^3^*J* = 5.9 Hz, H5a), 3.57–3.40 (m, 3H, H21a, H5b), 3.16–3.05 (m, 2H, H6a, H21b), 2.97 (dd, 1H, ^2^*J* = 16.1 Hz, ^3^*J* = 5.1 Hz, H6b), 2.64–2.56 (m, 1H, H14a), 2.20 (qd, ^3^*J* = 11.1 Hz, ^3^*J* = 3.0 Hz, 1H, H15), 2.12 (dd, ^3^*J* = 11.5 Hz, ^3^*J* = 2.2 Hz, 1H, H16), 1.83–1.77 (m, 1H, H18a), 1.72–1.61 (m, 1H, H20), 1.52–1.44 (m, 2H, H19a, H18b), 1.41–1.32 (m, 2H, H14b, H19b) ppm. ^13^C{^1^H} NMR (126 MHz, DMSO-*d*_6_): δ 172.4 (C1′), 136.4 (C13), 129.3 (C2), 125.8 (C8), 122.0 (C11), 119.3 (C10), 118.2 (C9), 111.5 (C12), 105.4 (C7), 66.2 (C17), 60.5 (C3), 57.3 (C21), 52.4, 52.3 (C4′), 51.9 (C5), 51.6 (C16), 40.3 (C3′), 37.9 (C20), 33.8 (C15), 31.7 (C18), 31.0 (C14), 22.2 (C19), 18.7 (C6) ppm. HRMS (ESI+): *m*/*z* [M + H]^+^ calculated for C_23_H_31_O_2_N_4_ = 395.24415, found: 395.24403.

#### 4.1.32 10,11-Dibromo-17-α-hydroxyyohimban-16-α-carboxylic Acid Methyl Ester (**6**)

FeCl_3_·6H_2_O (0.34 g, 1.27 mmol) and Br_2_ (58 μL, 1.13 mmol) were suspended in CHCl_3_ (10 mL) at 0 °C, and the mixture was stirred at 0 °C for 1 h. A solution of yohimbine (**1**) (free base) (0.10 g, 0.28 mmol) in CHCl_3_ (5 mL) was added at 0 °C, and the reaction mixture was stirred for 10 min at 0 °C. Sat. NaHCO_3_ (5 mL) was added, and the mixture was extracted with CHCl_3_ (3 × 10 mL). The organic phase was dried over Na_2_SO_4_ and filtered, HCl (1.0 mL) was added, and volatiles were evaporated. The crude residue was adsorbed on alumina (neutral) and eluted with CH_2_Cl_2_-MeOH mixture (0–2%). Purification on an HPLC column (Amylose SA) in hexane/*i*PrOH/diethylamine (80:20:0.4) afforded **6** (28 mg, 19%) as a white solid. ^1^H NMR (500 MHz, DMSO-*d*_6_): δ 11.11 (bs, 1H, ^1^N–H), 7.73 (s, 1H, H9), 7.59 (s, 1H, H12), 4.63 (d, 1H, ^3^*J* = 4.7 Hz, OH), 4.10 (m, 1H, H17), 3.65 (s, 3H, OCH_3_), 3.23 (m, 1H, H3), 2.97 (ddd, 1H, ^2^*J* = 11.6 Hz, ^3^*J* = 5.9 Hz, ^3^*J* = 1.6 Hz, H5a), 2.83 (dd, 1H, ^2^*J* = 11.0 Hz, ^3^*J* = 3.3 Hz, H21a), 2.71 (m, 1H, H6a), 2.58 (m, 1H, H6b), 2.46 (td, 1H, ^3^*J* = 12.2 Hz, ^3^*J* = 3.0 Hz, ^3^*J* = 3.0 Hz, H5b), 2.36 (dt,1H, ^3^*J* = 12.2 Hz, ^3^*J* = 3.0 Hz, ^3^*J* = 3.0 Hz, H14a), 2.26 (dd, 1H, ^3^*J* = 11.5 Hz, ^3^*J* = 2.8 Hz, ^3^*J* = 2.8 Hz, H16), 2.11 (t, 1H, ^3^*J* = 10.5 Hz, H21b), 1.85 (qd, 1H, ^3^*J* = 11.5 Hz, H15), 1.74**-**1.54 (m, 2H, H18), 1.44–1.32 (m, 2H, H20, 19a), 1.25 (m, 1H, H19b), 0.98 (m, 1H, H14b) ppm. ^13^C{^1^H} NMR (126 MHz, DMSO-*d*_6_): δ 172.9 (C1′), 138.8 (C2), 136.1 (C13), 128.0 (C8), 122.0 (C9), 115.7 (C12), 114.0 (C11), 112.6 (C10), 106.6 (C7), 66.8 (C17), 61.2 (C21), 59.9 (C3), 52.3 (C5), 52.2 (C16), 51.3 (OCH_3_), 39.7 (C20), 36.0 (C15), 33.9 (C14), 32.7 (C18), 23.1 (C19), 21.5 (C6) ppm. HRMS (ESI+): *m*/*z* [M + H]^+^ calculated for C_21_H_25_O_3_N_2_Br_2_ = 511.0226, found: 511.0217.

#### 4.1.33 [^3^H]-Yohimbine ([^3^H]-**1**)

The one-neck 1.5 mL tritiation flask equipped with a magnetic stir bar was charged with compound **6** (4.0 mg, 7.8 μmol), 10% Pd/C (1.6 mg), TEA (22 μL = 16 mg, 158 μmol), and MeOH (1.0 mL). The flask was connected to the tritiation manifold, and the reaction mixture was degassed three times in a repeated freeze–thaw cycle using a liquid nitrogen bath. The carrier-free tritium gas was released over the frozen reaction mixture, and the pressure was adjusted to 340 mbar at −196 °C (approximately 9 Ci/333 GBq). The mixture was brought to RT and vigorously stirred for 150 min. Subsequently, the reaction mixture was frozen again with liquid nitrogen, and excessive tritium gas was reabsorbed in the uranium bed. After thawing, the catalyst was filtered off using a PTFE syringe filter (0.22 μm). Both the flask and the filter were washed with MeOH (3 × 1 mL) and with MeOH/H_2_O (1:1) mixture (1 mL). To remove labile activity, the combined filtrates were evaporated; two other evaporations from methanol (5.0 mL) were performed. The activity of the crude product was determined as 465 mCi (17.2 GBq). A portion (13%) of this product was subjected to a semipreparative Phenyl-Hexyl 250 × 10 mm column (Phenomenex, eluent H_2_O/MeCN with 0.1% of TFA in both mobile phases, gradient 90% to 57% in 40 min); the activity of isolated [^3^H]-**1** was determined as 39.7 mCi (1.47 GBq) with radiochemical purity (R.C.P.) > 99%. The specific activity (S.A.) was calculated from MS as 45.7 Ci/mmol (1.69 TBq/mmol) or 1.57 ^3^H atoms per molecule. The product contains 9.3% of the unlabeled material. ^3^H NMR (320 MHz, MeOH-*d*_4_): δ 7.21 (td, *J* = 8.4, 1.2 Hz, ^3^H), 7.12 (td, *J* = 8.2, 1.1 Hz, ^3^H). MS (ESI+): *m*/*z* [M + H]^+^ calculated for C_21_[^1^H]_24_[^3^H]_2_O_3_N_2_ = 359.2, found: 359.4. Both UV spectrum and retention time are in accordance with unlabeled standard **1**.

### 4.2 Biological Evaluation

#### 4.2.1 Calcium Flux Assays on Cells Overexpressing ADRA2A or ADRA1A Adrenergic Receptors

**Figure 7:**
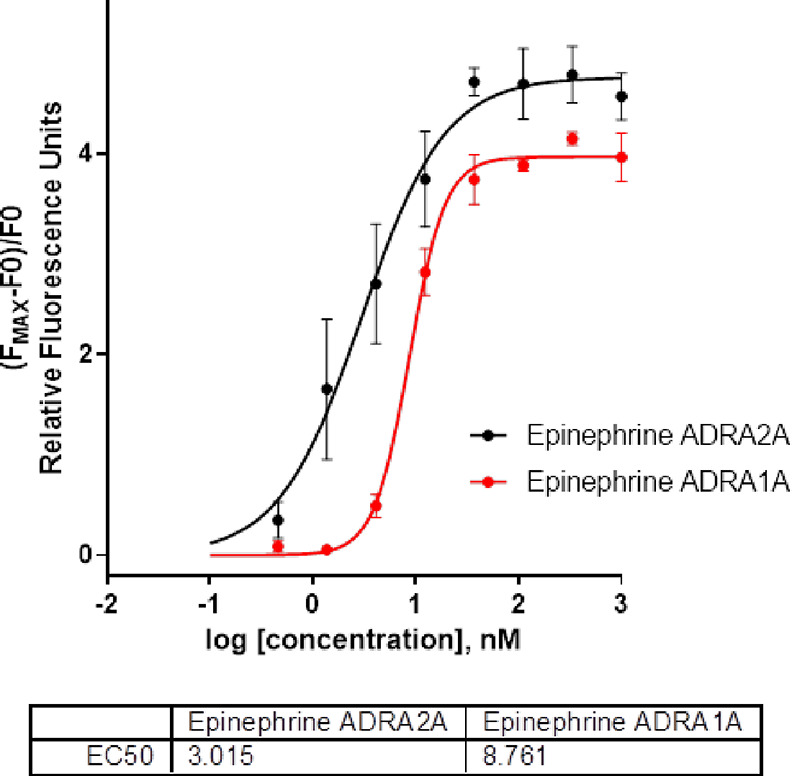
Representative data showing the activation of ADRA2A and ADRA1A adrenergic receptors expressed in Chem-1 cells induced by epinephrine. The cells were loaded with Fluo-8 AM, and calcium flux response was determined by the Spark multimode reader from Tecan. Measurement was performed in tetraplicate.

Chem-1 cells overexpressing ADRA2A or 1A adrenergic receptors were purchased from Merck Millipore (cat. #: HTS096RTA and HTS087RTA; Merck, Germany). The cells were cultured according to the manual. The day before the experiment, the cells were seeded 10,000 cells per well into black 384-well format micro plates with transparent bottom (cat. #: 142761, Nunc, Thermo Fisher Scientific, USA). The next day, the culture medium was changed for the assay buffer consisting of HBSS (cat. #: H6648-500 ML, Sigma-Aldrich, Merck, Germany), 20 mM HEPES (cat. #: L0180-100, Biowest, France), and 2.5 mM probenecid (cat. #: P8761, Sigma-Aldrich, Merck, Germany), adjusted for pH 7.4, and supplemented with 4.77 μM CalciFluor Fluo-8, AM (cat. #: 1345980-40-6, Santa Cruz Biotechnology, USA). After 30 min of incubation with the assay buffer at 37 °C and 5% CO_2_, the cells were incubated at RT in a dark place for another 15 min. After this time, the EC_80_ of the agonist epinephrine (cat. #: E4642-5G, Sigma-Aldrich, Merck, Germany) and the agonistic effect of the tested compounds were measured prior to the antagonistic effect. For the antagonistic effect of the positive control yohimbine and tested compounds, the cells were further incubated at RT in a dark place for another 15 min followed by adding EC_80_ of epinephrine. All compounds were added by an Agilent Bravo Automated Liquid Handling Platform (Agilent Technologies, USA). The kinetics was measured by the Spark multimode reader (Tecan Group Ltd., Switzerland) for 30 s (excitation wavelength 485 nm, emission wavelength 535 nm, bandwidth 10 nm). Before each kinetic measurement, the background fluorescence (F0) was measured, and the result was calculated as (Fmax – F0)/F0. Each compound was tested in seven concentrations (10 μM to 0.1 nM) in tetraplicate, and the IC_50_ value was calculated by GraphPad Prism (v 9.5.1. GraphPad Software, Boston, USA) using the log(inhibitor) vs response variable slope (four-parameter) model. EC_50_ of epinephrine on the cells was comparable to that provided by the manufacturer (Figure 7).

#### 4.2.2 Selectivity Profiling toward Off-Targets

A functional (agonist and antagonist) profile of compound **4n** and yohimbine toward off-targets (ADRA2B, ADRA2C, ADRA1A, and 5-HT1A) and a major target (ADRA2A) was performed by Multispan Inc. Brimonidine was used as a control agonist for ADRA2A, ADRA2B, and ADRA2C, whereas epinephrine and 5-hydroxytryptamine (5-HT) were used for ADRA1A and 5-HT1A, respectively. CHO or HEK293T cells overexpressing individual receptors of interest were seeded in a 384-well plate at an appropriate density. The calcium assay was conducted according to the manufacturer’s protocols (MULTISCREEN Calcium 1.0 No Wash Assay Kit). The calcium dye loading buffer was added to the cells and incubated for 1 h at 37 °C. Cells were injected with control agonist or test compound by FLIPR, and calcium mobilization was monitored for 180 s with the compound injected into the wells at the 19th second. Cells with test compounds were allowed to incubate for a total of 15 min at room temperature. Subsequently, for antagonist mode reading, the same wells were then injected with control agonists at the EC_80_ concentration. Calcium mobilization was again monitored for 180 s with EC_80_ injected into the wells at the 19th second. Fluorescent emissions were read at 525 nm with excitation at 490 nm in a FLIPR384 instrument (Molecular Devices). Dose–response curves were fitted using the “Sigmoidal dose–response (variable slope)” function in GraphPad Prism 6. EC_50_ values were calculated based on the fitted curves.

#### 4.2.3 Cytotoxicity Assay

The cytotoxicity of the compounds was evaluated in nontumor normal human dermal fibroblasts (NHDF) purchased from ATCC (PCS-201-012, Manassas, VA, USA). The cells were negatively tested for the presence of mycoplasma (cat. #: LT07-318 MycoAlert Mycoplasma Detection kit, Lonza). The cells were cultivated in DMEM high glucose 4.5 g/L medium (cat. #: R8758, Merck, Germany). The medium was supplemented with 10% fetal bovine serum (cat. #: F7524, Merck, Germany; inactivated at 56 °C for 30 min) and 1.0% of l-glutamine (cat. #: G7513, Merck, Germany) without antibiotics. Cells were seeded in 384-well format white micro plates (cat. #: 164610, Nunc, Thermo Fisher Scientific, USA) at a concentration of 60,000 cells per mL and left to rest overnight. Next day, 1 and 10 μM concentrations of the tested compounds were added in triplicates. The cells were cultivated at 37 °C and 5% CO_2_ for 72 h after which a luminescent cell viability assay (CellTiter-Glo 2.0, cat. #: Promega, Madison, USA) was added. The plate was left on a shaker (350 rpm) in the dark for 20 min at RT. Luminescence was measured by a Spark multimode reader from Tecan (Tecan Group Ltd., Switzerland). The signal of the compound-treated cells was related to the value of the untreated control, which was arbitrarily set to 100% viability.

#### 4.2.4 Radioligand Binding Assay

The radioligand binding assay was performed on Chem-1 cells with overexpression of ADRA2A adrenergic receptors as described in Section 4.2.1. The day before the experiment, 10,000 cells per well were seeded into white 384-well format micro plates with transparent bottom (cat. #: 142762, Nunc, Thermo Fisher Scientific, USA). Next day, the cells were washed with wash buffer (20 mM TRIS, 118 mM NaCl, 4.7 mM KCl, 5 mM MgCl_2_, pH = 7.4) and incubated in binding buffer (50 mM TRIS, 118 mM NaCl, 4.7 mM KCl, 5 mM MgCl_2_, 2g/L glucose, 0.1% BSA, pH = 7.4) with 3 nM [^3^H]-yohimbine (prepared in-house according to the above-mentioned procedure) and tested compounds at RT. After 30 min of incubation, the cells were washed three times with wash buffer and lysed in MicroScint-PS liquid scintillation cocktail (cat. #: 6013631, PerkinElmer, USA). The plates were measured in a MicroBeta^2^ Microplate Counter (PerkinElmer, USA) using a normal read. The IC_50_ values of compounds were calculated by GraphPad Prism (v 9.5.1. GraphPad Software, Boston, USA) using the log(inhibitor) vs response variable slope (four-parameter) model. The *K*_D_ value for [^3^H]*-*yohimbine was calculated using a one-sided total model. The *K*_D_ values for cold yohimbine and **4n** were calculated using the One Side-Fit *K*_i_ model.

#### 4.2.5 SPR Microscopy

For measurement of binding activities of yohimbine and **4n** directly on ADRA2A overexpressing Chem-1 cells, we used the label-free cell-based surface plasmon resonance microscopy (SPRM) system SPRm 200 (Biosensing Instrument, iBiotech, West Midlands, U.K.). The cells were seeded on a gold sensor chip with a silicone well for 24 h before the experiment in the concentration of 80,000 cells per well in a standard medium. Before the experiment, the wells were washed three times with assay buffer (HBSS with 20 mM HEPES and 0.1% BSA, pH = 7.4), which was used for the whole procedure. The cells were treated with the following concentrations: 0.14, 0.41, 1.2, 3.7, 11.1, 30, 100, and 300 nM and 1 and 3 μM for both yohimbine and **4n**. The program for incomplete dissociation was used to measure the dissociation constant (*K*_D_). The ImageSPR software was used to analyze the data and calculate the *K*_D_ values.

#### 4.2.6 Plasma Stability Assay

To determine the plasma stability of the compounds, 5 μM of these was incubated with human pooled plasma from 50 donors (Biowest) for 120 and 240 min at 37 °C. The reactions were terminated by adding 4 vol of ice-cold methanol, and the samples were then mixed vigorously, frozen at −20 °C for 1 h, and left overnight at 8 °C. After that, the samples were centrifuged, and the supernatant was analyzed by means of LC–MS/MS Sciex 6500 triple quadrupole (Sciex, Framingham, MA, USA). Zero time points were prepared by adding ice-cold methanol to the compound prior to the addition of the plasma.

#### 4.2.7 Microsomal Stability

Microsomal stability assay was performed using the 0.5 mg/mL human pooled liver microsomal preparation (Thermo Scientific) and 10 μM compounds in 90 mM TRIS-Cl buffer pH = 7.4 containing 2 mM NADPH and 2 mM MgCl_2_ for 10, 30, and 45 min at 37 °C. The reactions were terminated by the addition of 4 vol of ice-cold methanol, mixed vigorously, frozen at −20 °C for 1 h, and left overnight at 8 °C. After that, the samples were centrifuged, and the supernatants were analyzed by means of the LC–MS/MS Sciex 6500 triple quadrupole (Sciex, Framingham, MA, USA). Zero time points were prepared by adding ice-cold methanol to a mixture of the compound with cofactors prior to the addition of microsomes. The microsomal half-lives (*t*_1/2_'s) were calculated using the equation *t*_1/2_ = 0.693/*k*, where *k* is the slope found in the linear fit of the natural logarithm of the fraction remaining of the parent compound vs incubation time. Intrinsic clearance (CL_int_) was calculated using the following formula:
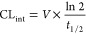
where *V* = incubation volume per milligram of microsomal protein (μL/mg) and *t*_1/2_ = microsomal half-life.

#### 4.2.8 Caco-2 Permeability Assay

The transepithelial bidirectional transport of tested compounds (5 μM, pH 7.4/7.4) across the Caco-2 monolayers was studied using the BD BioCoat HTS Caco-2 assay system (BD Biosciences, Bedford, MA) as described in detail elsewhere.^47^ Aliquots were collected from the donor and acceptor compartment at the end of the transport period (3 h), the integrity of monolayers was verified using Lucifer Yellow dye, and the compounds were quantified using LC–MS/MS (Triple Quad 6500+, Sciex). The apparent permeability coefficient (*P*_app_) was calculated from the following equation: *P*_app_ = (d*Q*/d*t*)/*C*_0_ × *A*, where d*Q*/d*t* is the rate of absorption of the drug across the cells, *C*_0_ is the donor compartment concentration at time zero, and *A* is the area of the monolayer. The efflux ratio was expressed as (*P*_app_ B–A)/(*P*_app_ A–B). Recovery (%) was determined as (total compound mass in donor and receiver compartments at the end of the incubation/initial compound mass in the donor compartment) × 100.

#### 4.2.9 MDCKII-MDR1 Permeability Assay

Canine MDR1 Knockout, Human MDR1 Knockin MDCKII cells (Sigma-Aldrich) were employed to estimate CNS permeability of the compounds. Briefly, the cells were seeded at a density of 30,000 cells per 24-well plate insert of the Transwell sandwich (CellQART, SABEU GmbH&Co.KG, Germany). They were left to differentiate for 5 days with medium exchange every other day. Prior to the experiment, the medium from both apical and basolateral well was exchanged for HBSS buffer (containing 10 mM HEPES and 25 mM glucose) and left to equilibrate for 30 min. Compound permeability was assayed by replacing pure HBSS with HBSS containing 10 μM compounds and 220 μM Lucifer yellow (LY) solution in an apical insert (A to B transport) or in a basolateral well (B to A transport). The treated cells were then returned to the incubator for 2 h at 37 °C. After that, samples were collected from both apical inserts and basolateral wells and analyzed using LC–MS (Triple Quad 6500+, Sciex). LY permeation was used to check for the integrity of monolayer in each sample. The plate containing receiver samples was placed into a fluorescent reader, and the fluorescence was determined at 485 nm excitation and 535 nm emission. Only wells displaying LY permeability <2% were qualified for compound transport quantification. Apparent permeability coefficients (*P*_app_'s), efflux ratios, and recoveries for the compounds were calculated as described in Section 4.2.8. The data represent means ± SD of two independent experiments performed in triplicate.

#### 4.2.10 Molecular Modeling

The docking study was performed using the Molecular Operating Environment software (MOE) Crystal structures of the receptors ADRA2A (6KUX),^29^ ADRA2B (6K41),^30^ ADRA2C (6KUW),^31^ ADRA1A (AlphaFold model),^32^ and 5-HT_1A_ (7E2Z)^33^ were prepared with the MOE QuickPrep tool with default setup; the structure was not minimized. Structures of all final compounds were properly protonated and minimized to an RMS gradient of 0.001. For docking studies on ADRA2B and ADRA1A, an induced fit dock protocol was used; for the rest of the receptors, a rigid dock protocol was chosen with structures where molecules of water were excluded and ligand rotation bonds was enabled. The default placement and refinement methods were used with 50 retained structures after the first refinement and 10 retained structures after the second refinement. Pharmacophore restraints were applied for yohimbine and compound **4n** (features volume in brackets): hydrogen bond donor (1.4), aromatic ring (1.9). For all calculations, the Amber 10:EHT mixed force field was used with R-Field solvent model.

## Supporting Information Available

Kinetic solubility of selected compounds and copies of the NMR (^1^H and ^13^C), MS, HR-MS, and UPLC/MS spectra of the prepared compounds (PDF)
Molecular formula strings (CSV)

## ABBREVIATIONS USED

ADRA2A: alpha-2A adrenergic receptor
BBB: blood–brain barrier
BSA: bis(trimethylsilyl)acetamide
CL_int_: intrinsic clearance
D receptor: dopamine receptor
DBU: 1,8-diazabicyclo[5.4.0]undec-7-ene
DCM: dichloromethane
DIAD: diisopropyl azodicarboxylate
EDC: 1-ethyl-3-(3-(dimethylamino)propyl)carbodiimide
HATU: hexafluorophosphate azabenzotriazole tetramethyl uranium
5-HT receptor: 5-hydroxytryptamine receptor
IL: interleukin
MDCK cells: Madin–Darby canine kidney cells
NHDF: normal human dermal fibroblasts
RBA: radioligand binding assay
SI: selectivity index
TEA: triethylamine
TFA: trifluoroacetic acid
TMS: trimethylsilyl
TNF-alpha: tumor necrosis factor-alpha
