# Supplementary material for: Structure-Based Drug Design of ADRA2A Antagonists Derived from Yohimbine
Source: J Med Chem. 2024 Jun 10;67(12):10135–51. doi: 10.1021/acs.jmedchem.4c00323 (PMC11215778; doi:10.1021/acs.jmedchem.4c00323)
Supplement: Supplementary file 1 — jm4c00323_si_001.pdf [file jm4c00323_si_001.pdf]

SUPPORTING INFORMATION

Structure-based drug design of ADRA2A antagonists derived from yohimbine

Artem Chayka, Michal Česnek, Erika Kužmová, Jaroslav Kozák, Eva Tloušťová, Alexandra Dvořáková, Timotej Strmeň, Břetislav Brož, Zuzana Osifová, Martin Dračínský, Helena Mertlíková-Kaiserová*, Zlatko Janeba*

Institute of Organic Chemistry and Biochemistry of the Czech Academy of Sciences, Flemingovo nám. 2, 160 00 Prague 6, Czech Republic

* *helena.kaiserova@uochb.cas.cz; *janeba@uochb.cas.cz*

TABLE OF CONTENTS

Kinetic solubility of selected compounds (**Table S1**) ……..…………………… S2

The numbering system for ^{1}H NMR and ^{13}C NMR spectra (**Figure S1**) S3

Spectral data and characterization of prepared compounds …………………….. S4

Table S1. Kinetic solubility of selected compounds

Compound	Concentration (µM)
**1** (Yohimbine)	120.1 ± 7.8
**3a**	95.4 ± 8.5
**3b**	95.7 ± 6.8
**4a**	107.9 ± 3.3
**4c**	110.1 ± 0.5
**4d**	112.7 ± 0.1
**4g**	100.1 ± 6.1
**4i**	106.5 ± 2.1
**4j**	98.0 ± 5.9
**4k**	95.4 ± 1.8
**4l**	96.1 ± 2.0
**4m**	95.5 ± 1.8
**4n**	94.7 ± 1.9
**4o**	99.8 ± 2.8
**4p**	89.6 ± 0.2
**4q**	96.7 ± 1.0
**4s**	89.3 ± 1.5
**5a**	100.7 ± 1.7

Conditions: Phosphate-buffered saline (PBS), pH 7.4, compound concentration at 100 µM, incubation for 120 min at RT, LC-MS/MS analysis.

Results: Expressed as means of duplicates (Mean ± SD); calibration curves used to determine concentrations.

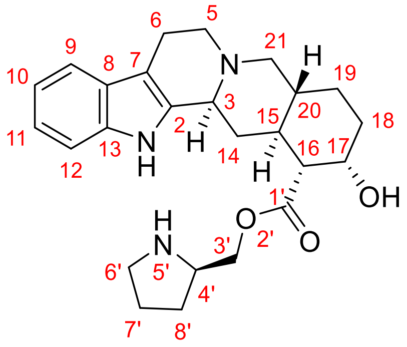

4a

Figure S1. The numbering system for ^{1}H NMR and ^{13}C NMR spectra of compound **4a**.

Spectral data and characterization of prepared compounds:

17-α-Hydroxyyohimban-16-α-carboxylic acid ethyl ester (3a)

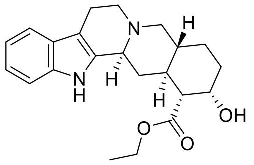

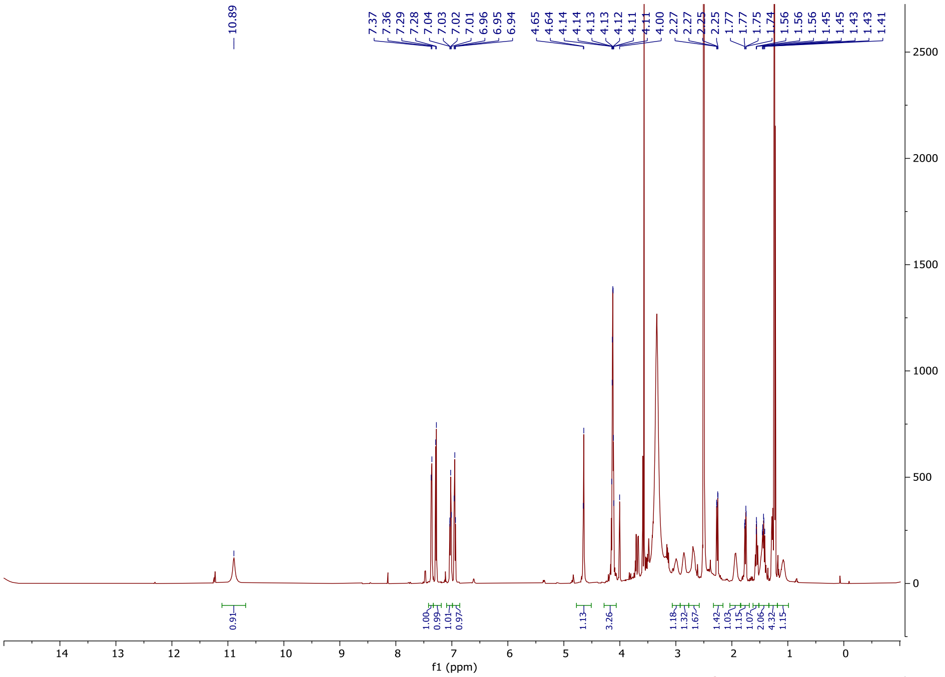

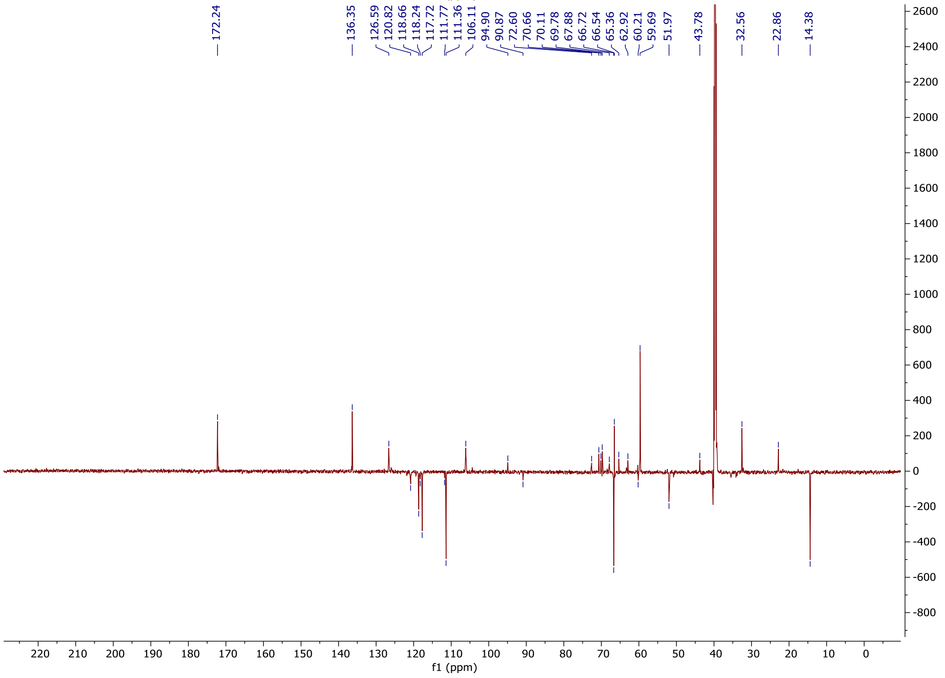

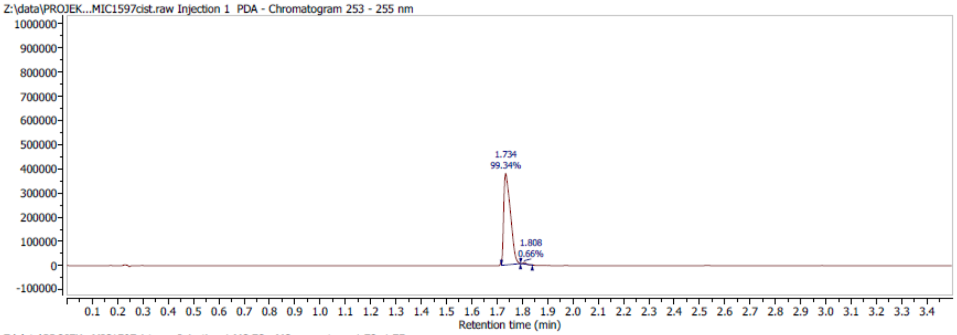

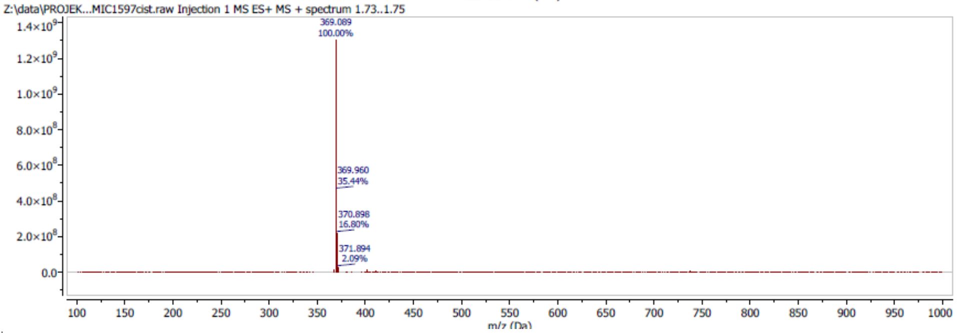

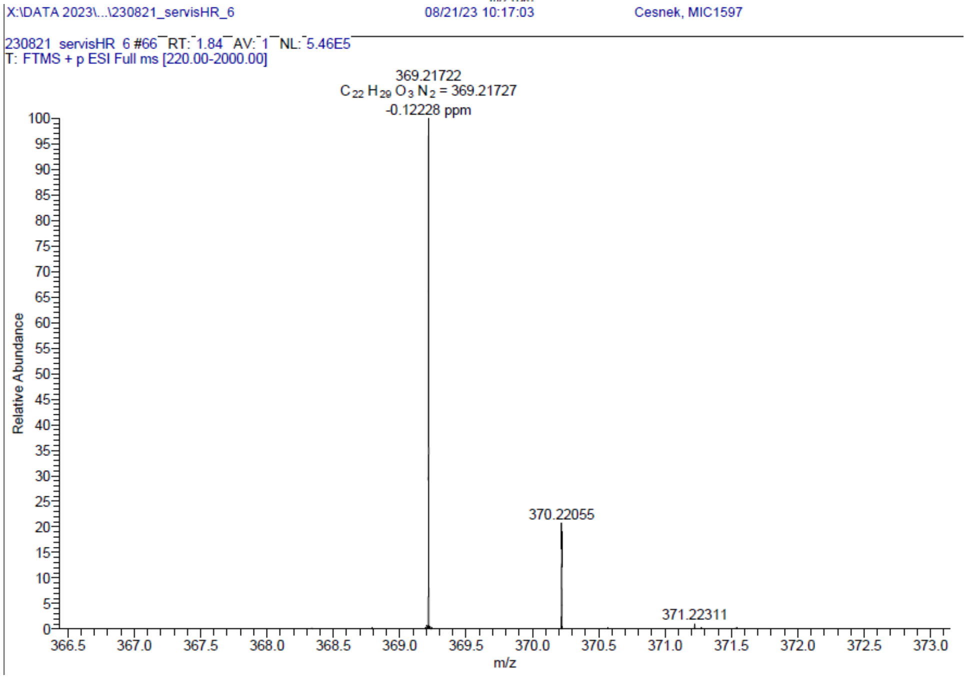

HRMS (ESI+): *m/z* [M + H]$^+$ calculated for $C_{22}H_{29}O_3N_2$ = 369.2173, found: 369.2172.

17-α-Hydroxyyohimban-16-α-carboxylic acid isopropyl ester (3b)

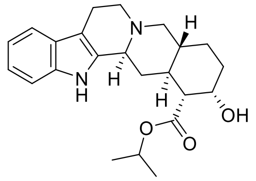

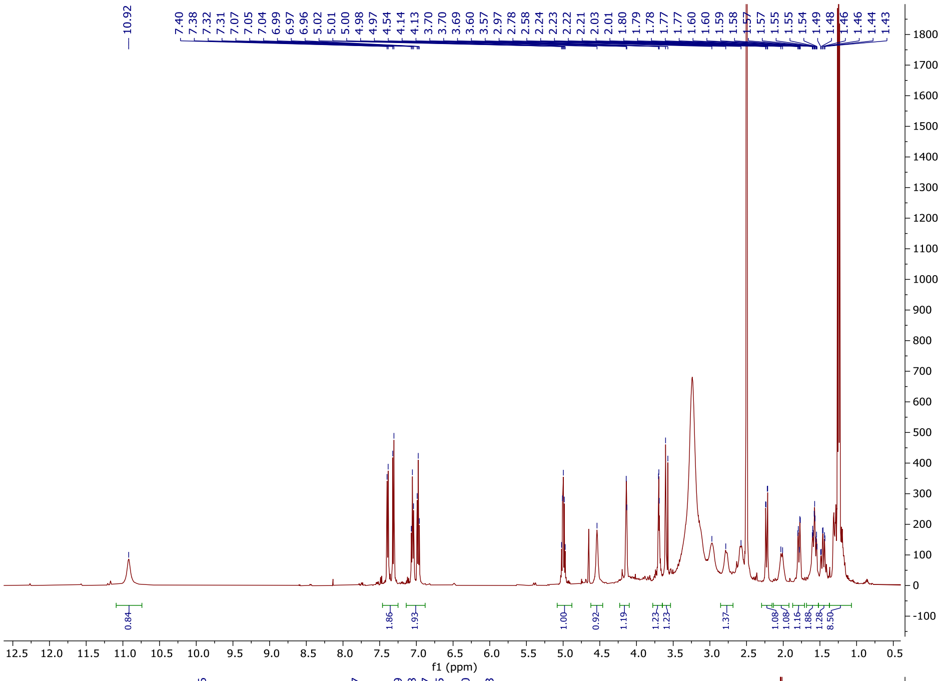

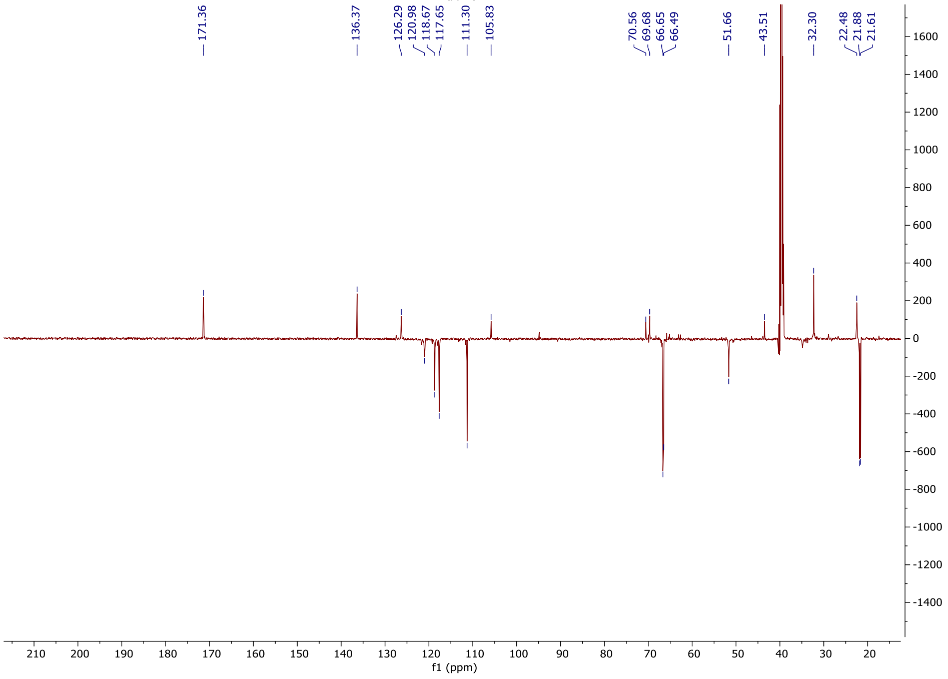

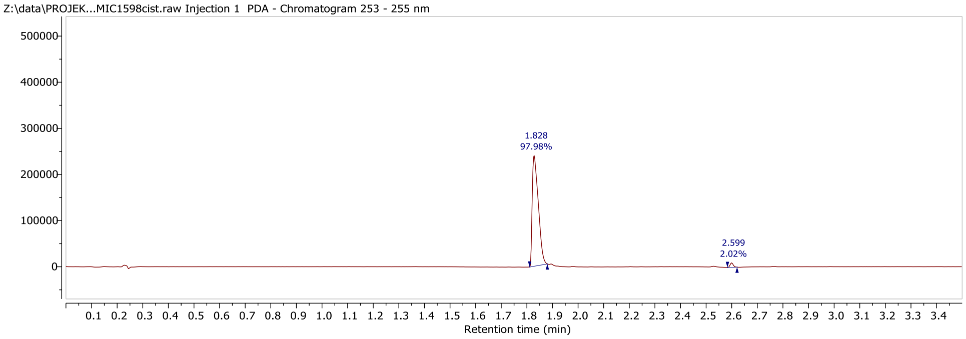

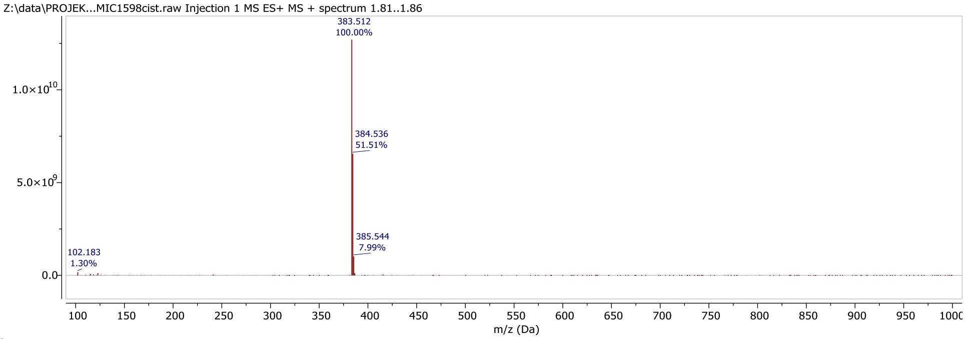

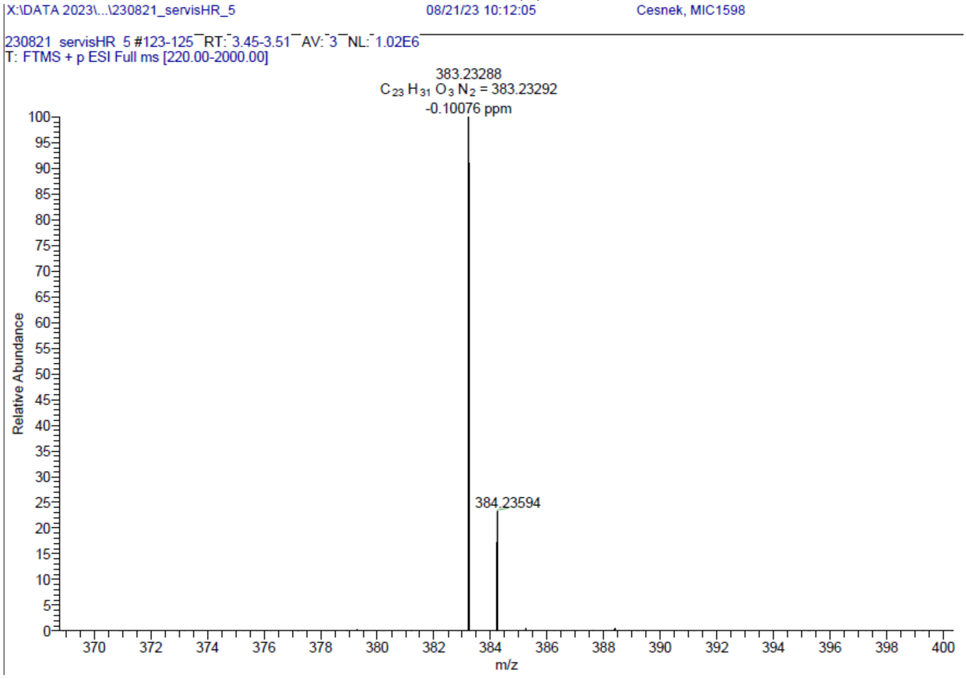

HRMS (ESI+): *m/z* $[M + H]^+$ calculated for $C_{23}H_{31}O_3N_2$ = 383.2329, found: 383.2329.

17-α-Hydroxyyohimban-16-α-carboxylic acid (3-methylbutyl-1-yl) ester (3c)

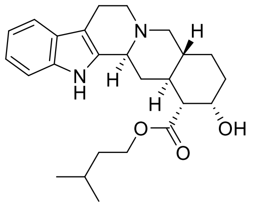

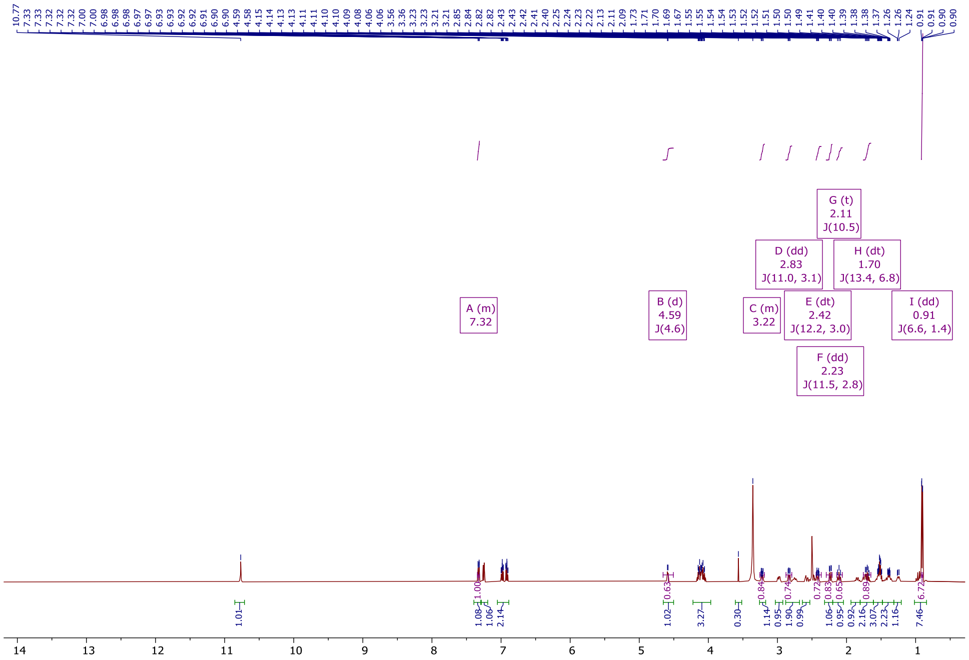

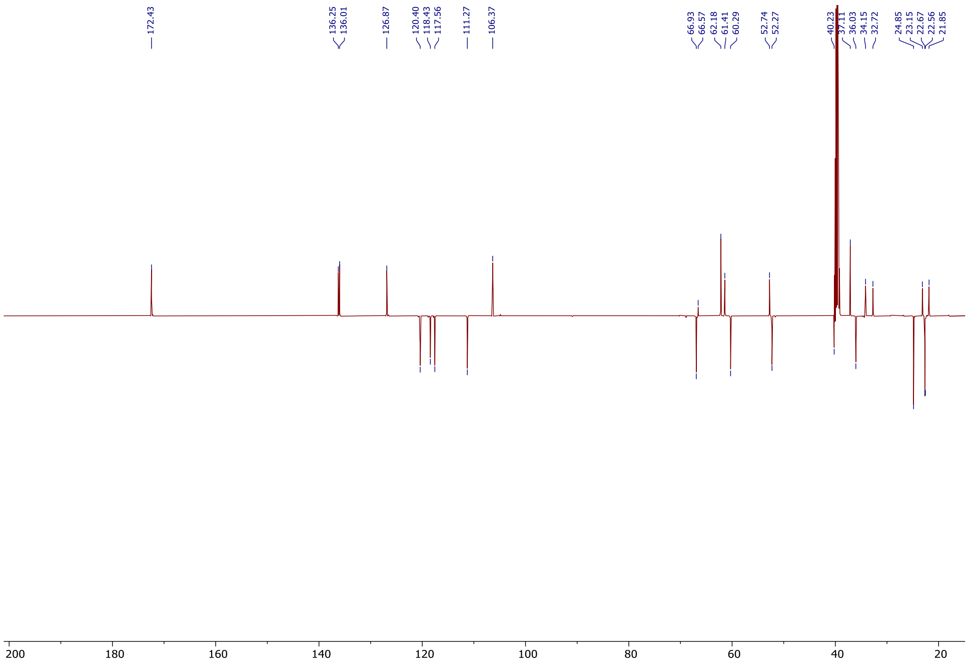

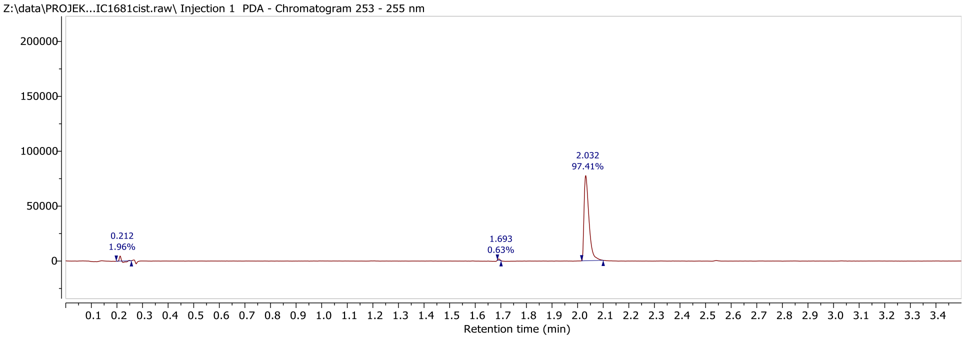

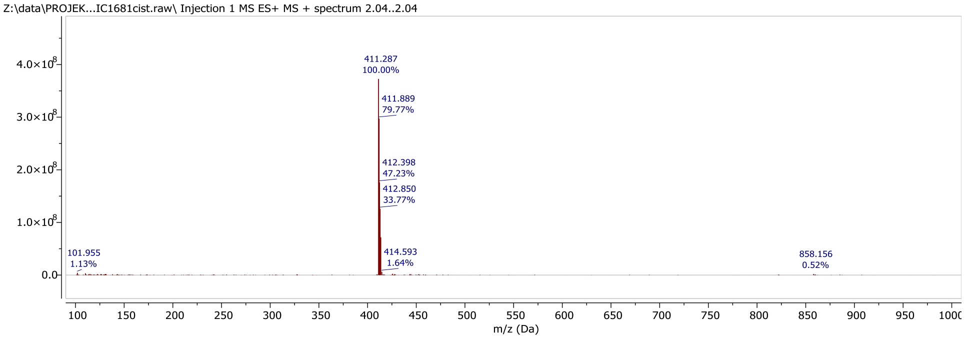

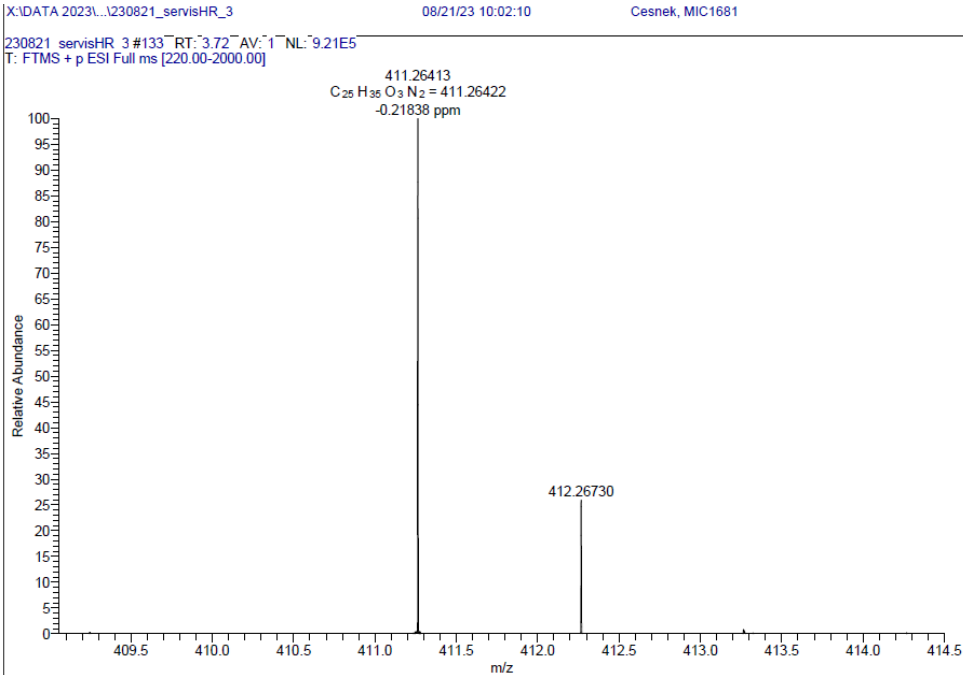

HRMS (ESI+): m/z $[M + H]^+$ calculated for $C_{25}H_{35}O_3N_2$ = 411.2642, found: 411.2641.

17-α-Hydroxyyohimban-16-α-carboxylic acid cyclopentyl ester (3d)

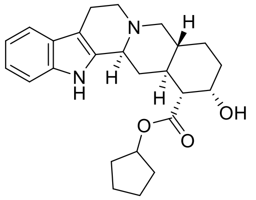

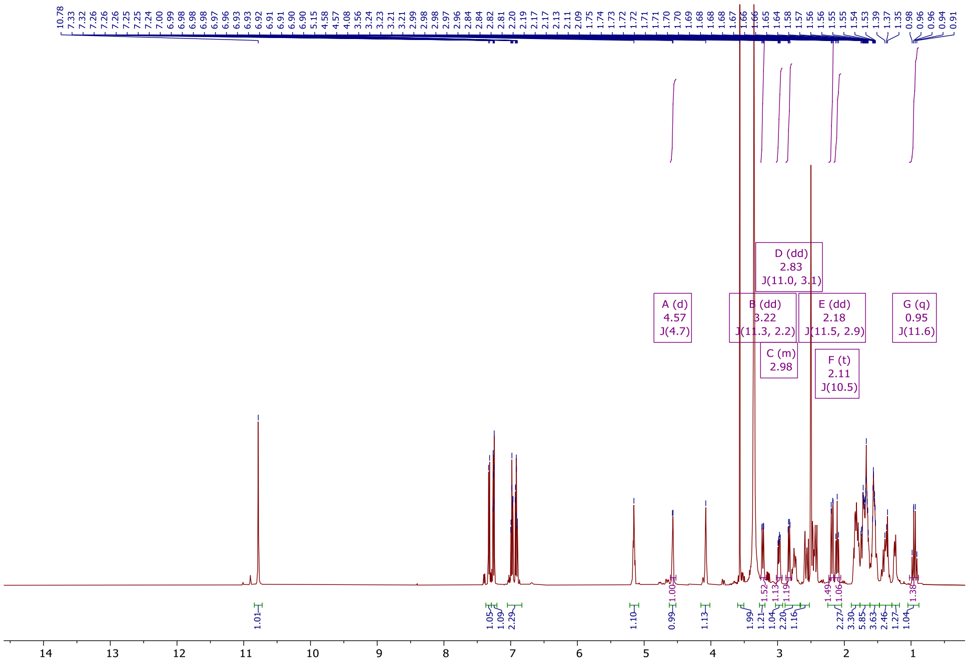

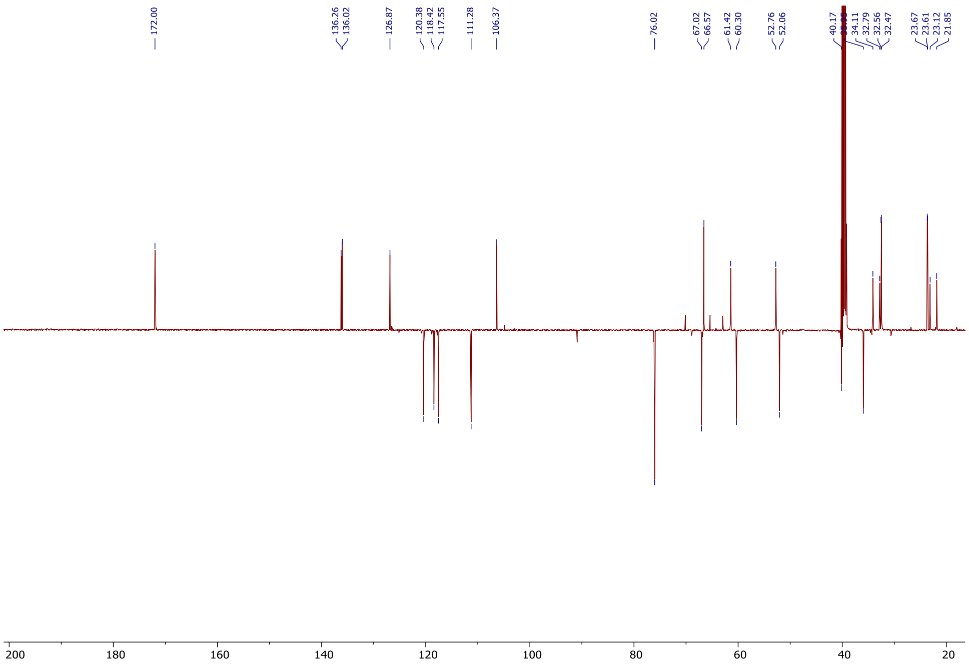

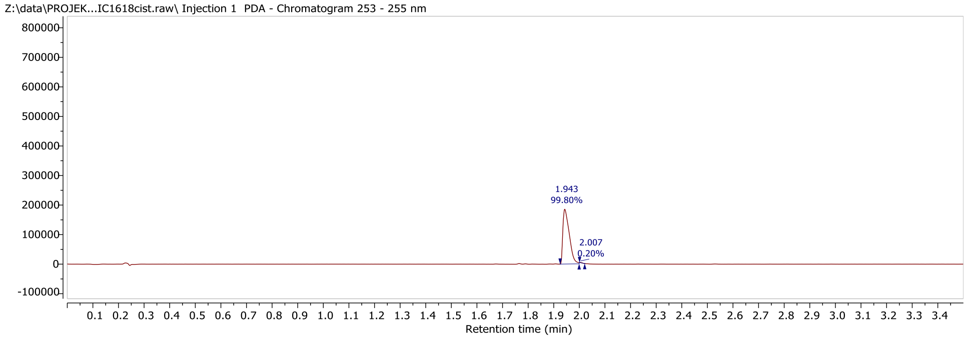

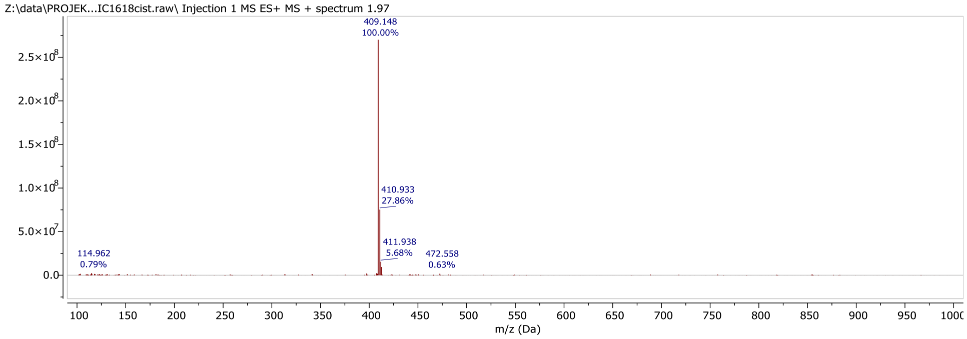

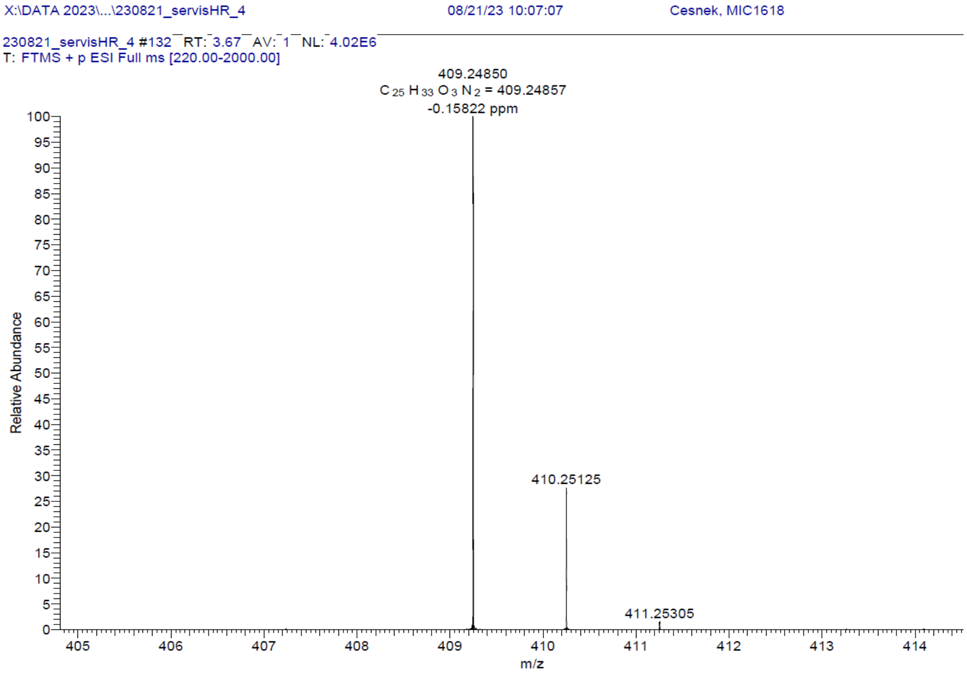

HRMS (ESI+): *m/z* $[M + H]^+$ calculated for $C_{25}H_{33}O_3N_2$ = 409.2486, found: 409.2485.

17-α-Hydroxyyohimban-16-α-carboxylic acid tert-butyl ester (3e)

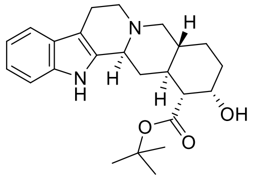

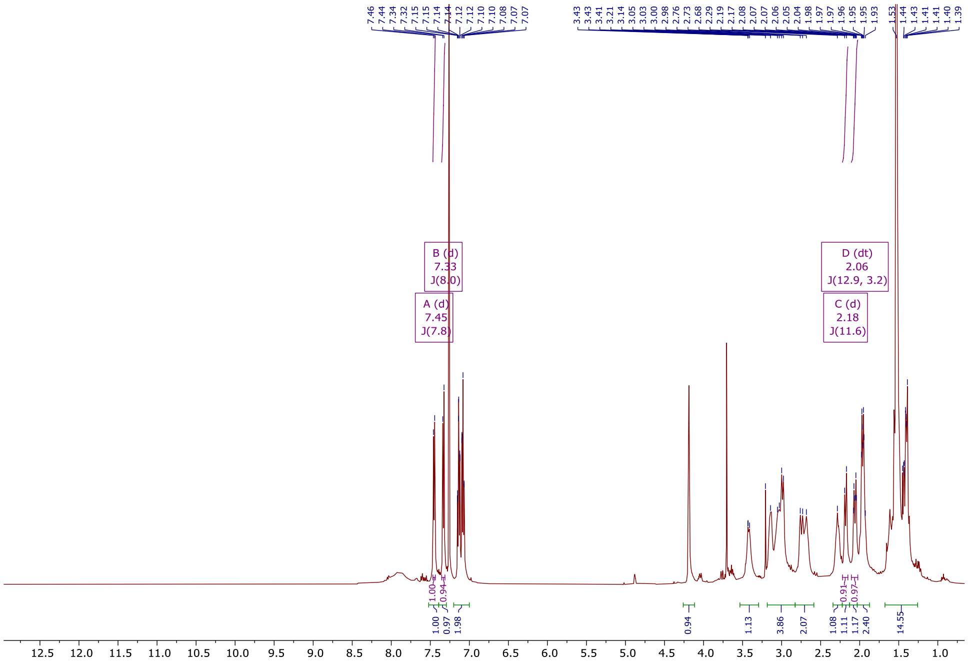

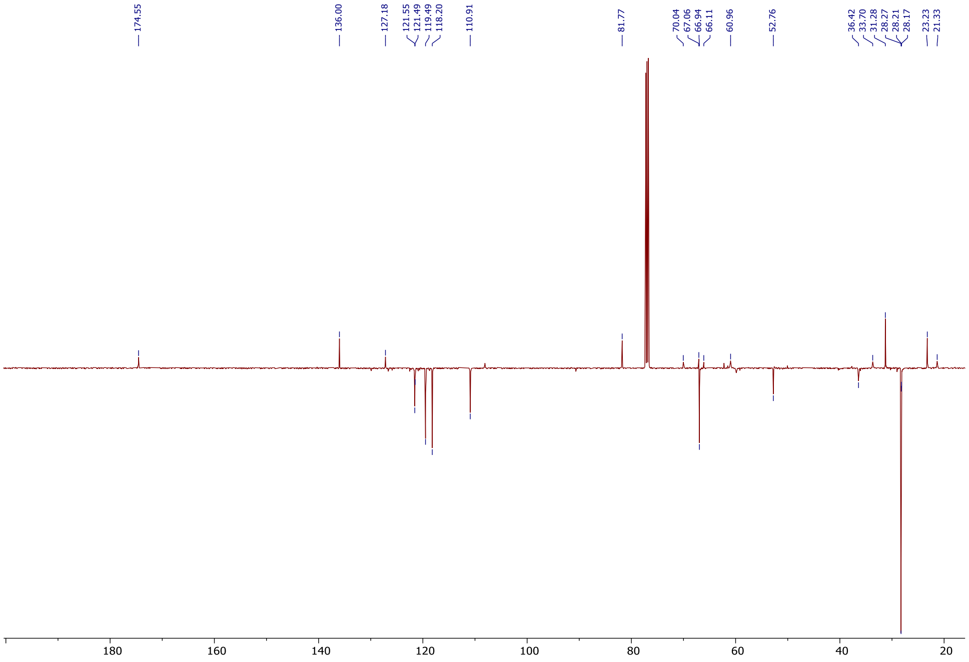

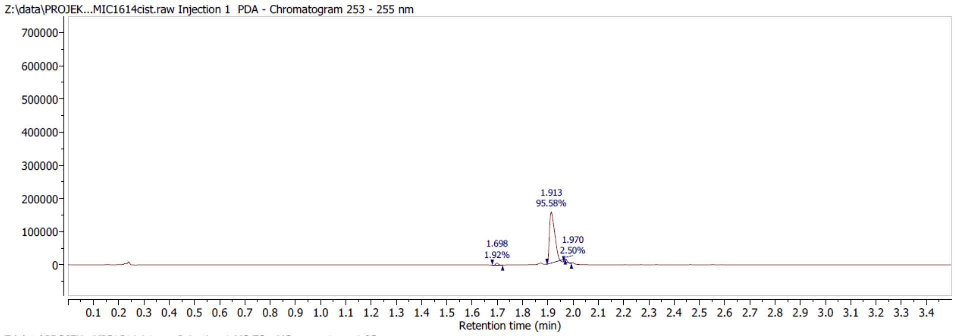

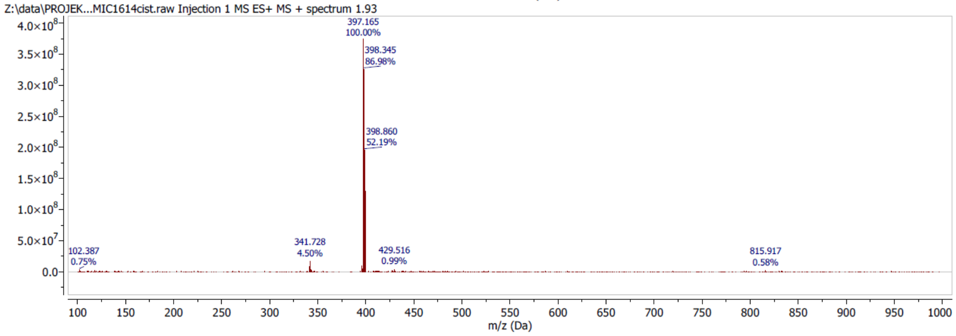

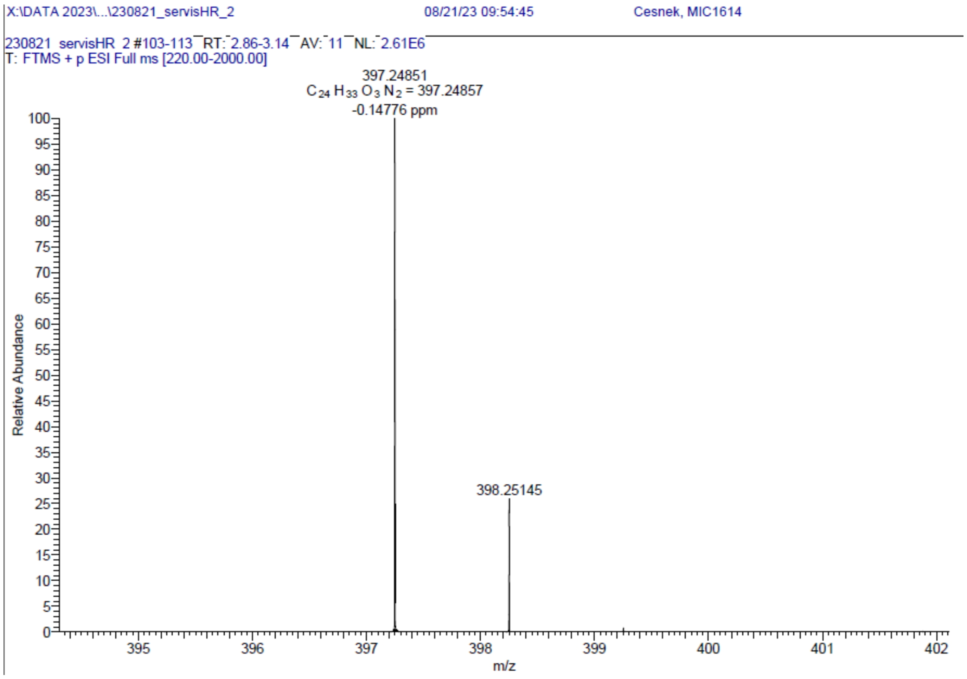

HRMS (ESI+): *m/z* $[M + H]^+$ calculated for $C_{24}H_{33}O_3N_2$ = 397.2486, found: 397.2485.

17-α-Hydroxyyohimban-16-α-carboxylic acid (*R*)-pyrrolidin-2-ylmethyl ester (4a)

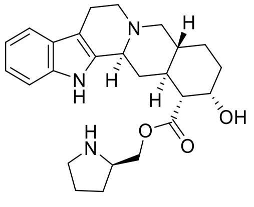

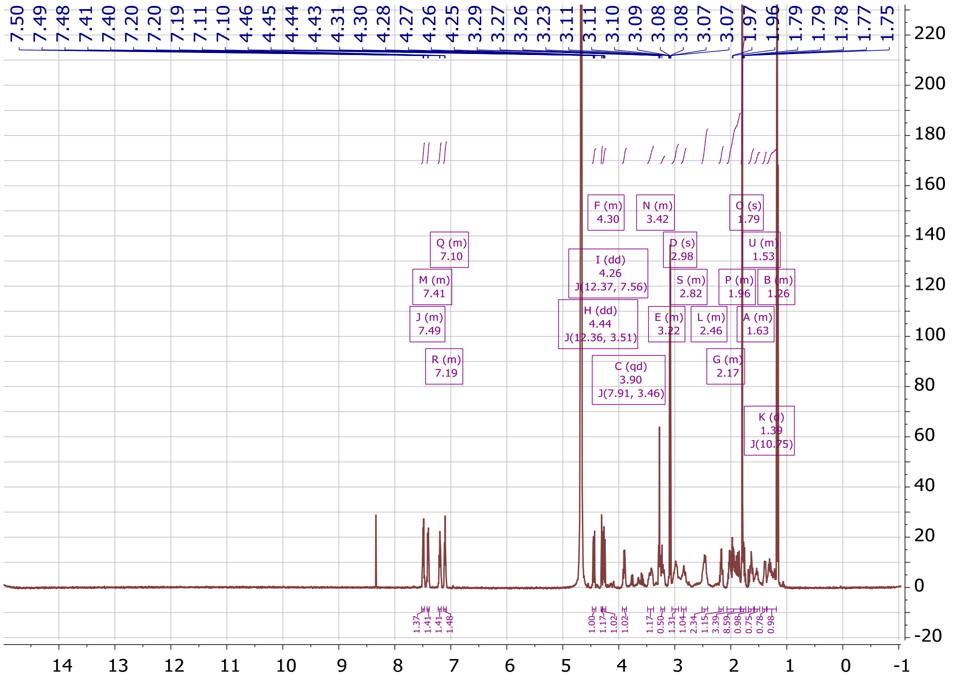

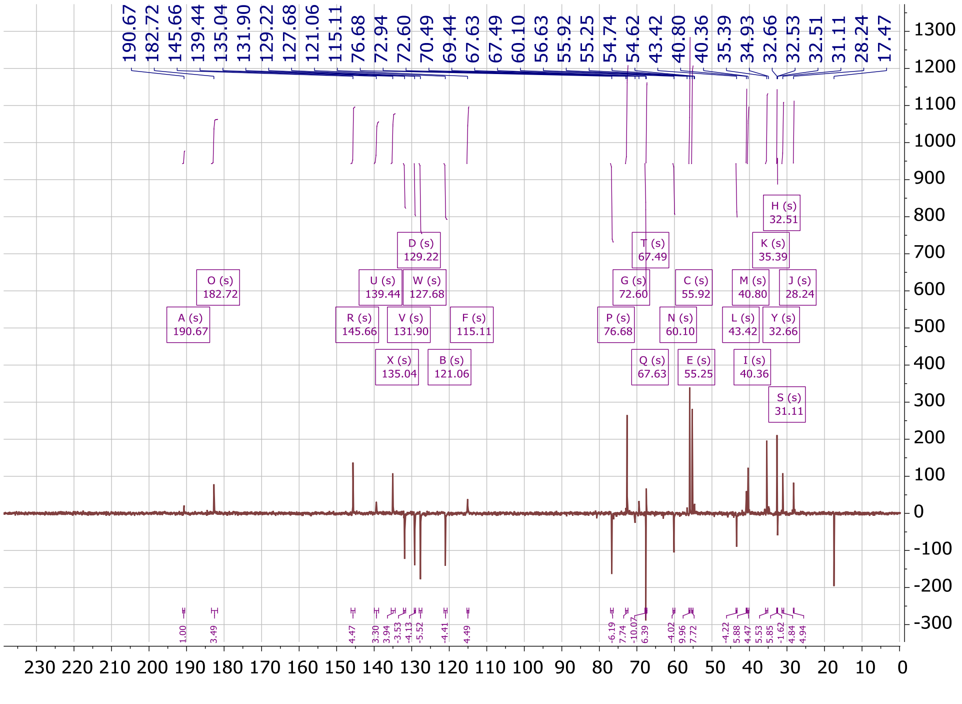

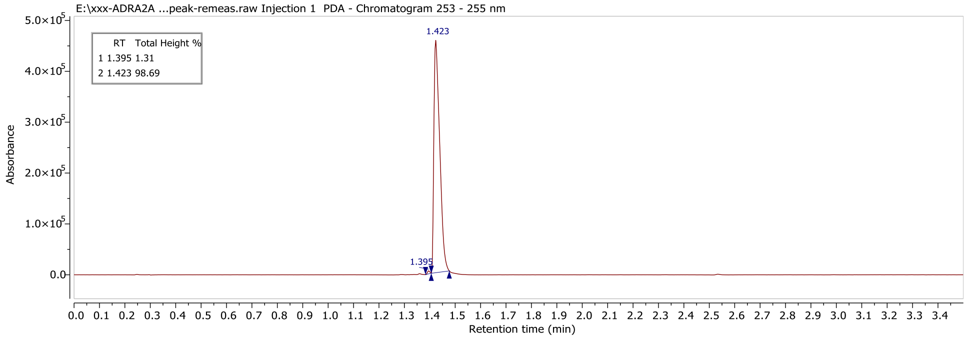

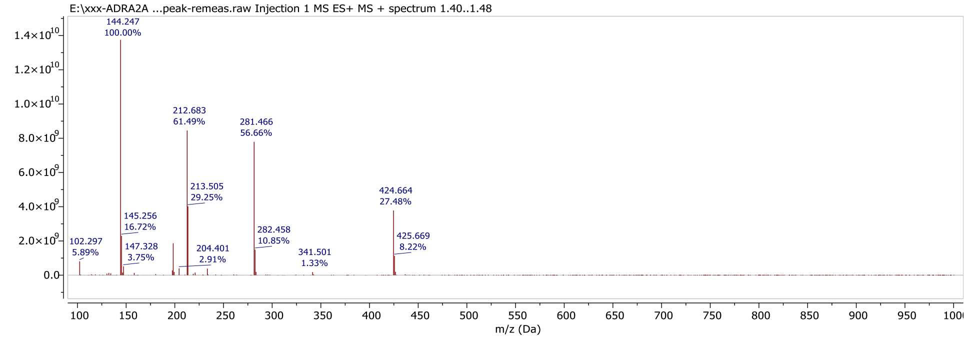

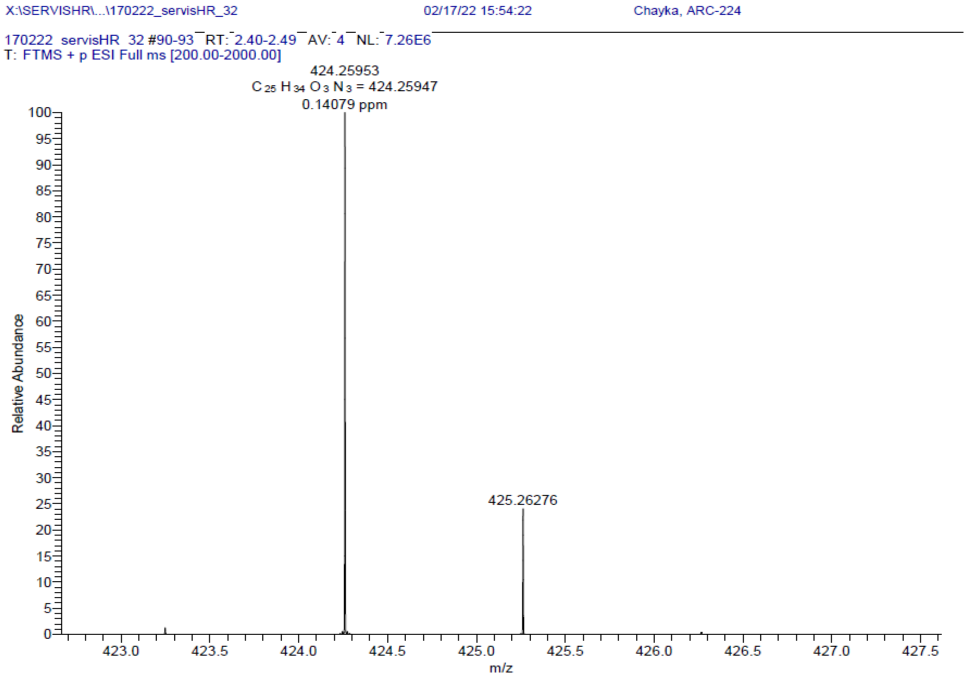

HRMS (ESI+): *m/z* $[M + H]^+$ calculated for $C_{25}H_{34}O_3N_3$ = 424.2595, found: 424.2595

17-α-Hydroxyyohimban-16-α-carboxylic acid (*S*)-pyrrolidin-2-ylmethyl ester (4b)

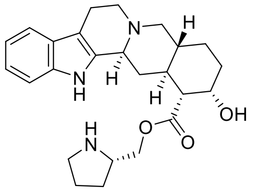

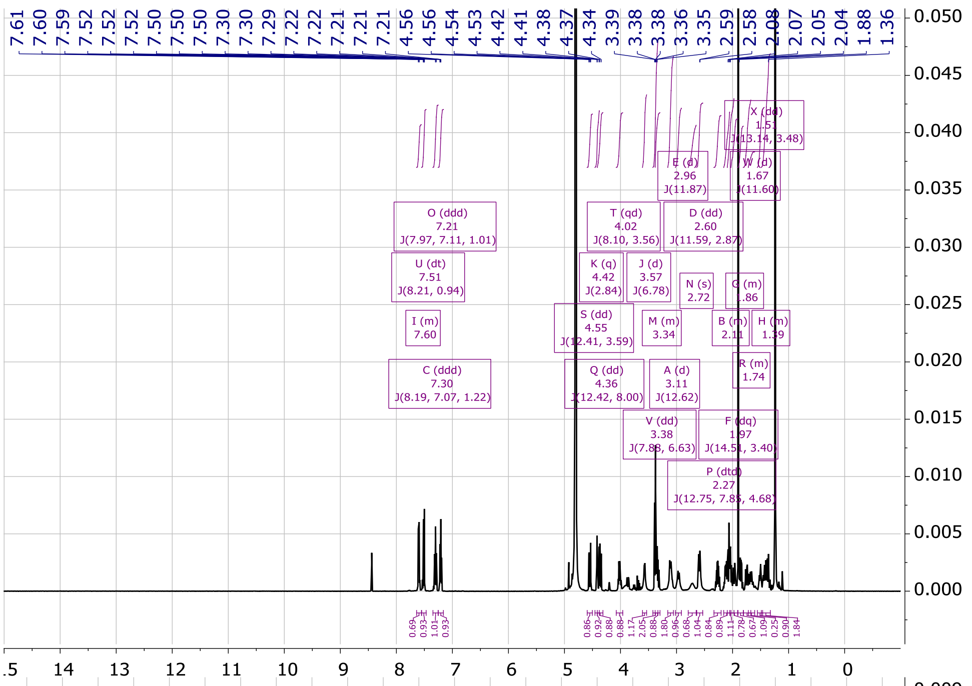

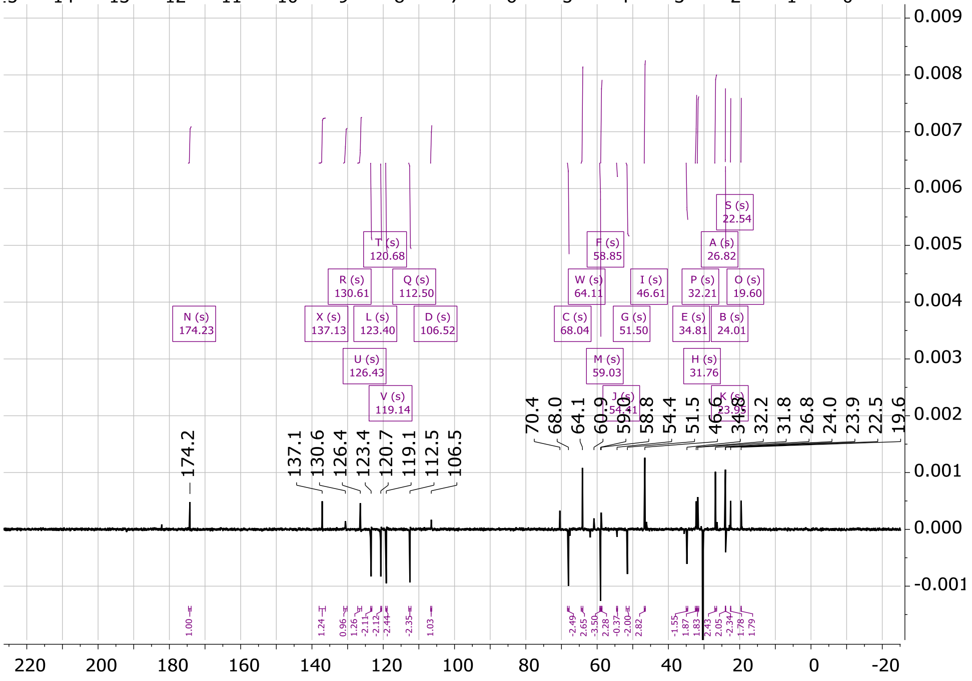

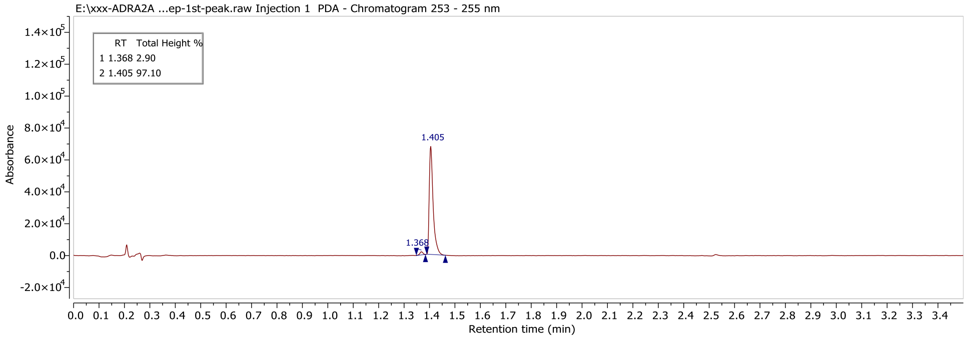

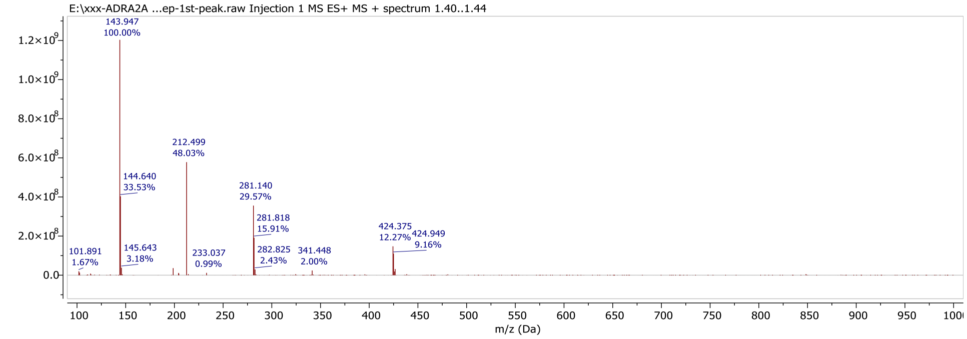

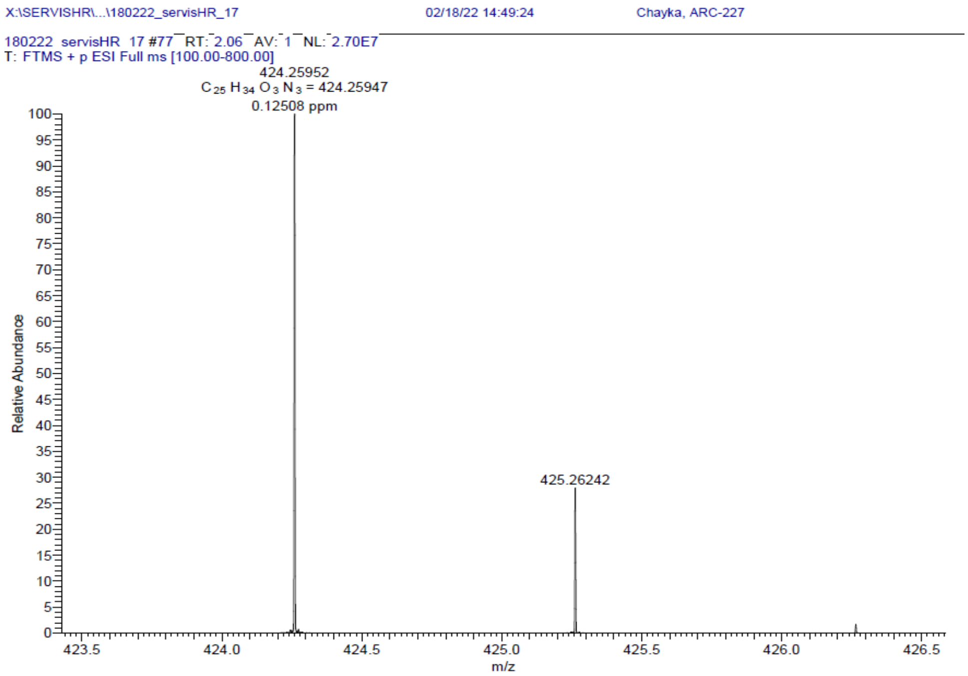

HRMS (ESI+): *m/z* [M + H]$^+$ calculated for $C_{25}H_{34}O_3N_3$ = 424.2595, found: 424.2595

17-α-Hydroxyyohimban-16-α-carboxylic acid 2-(methylamino)ethyl ester (4c)

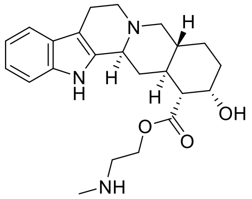

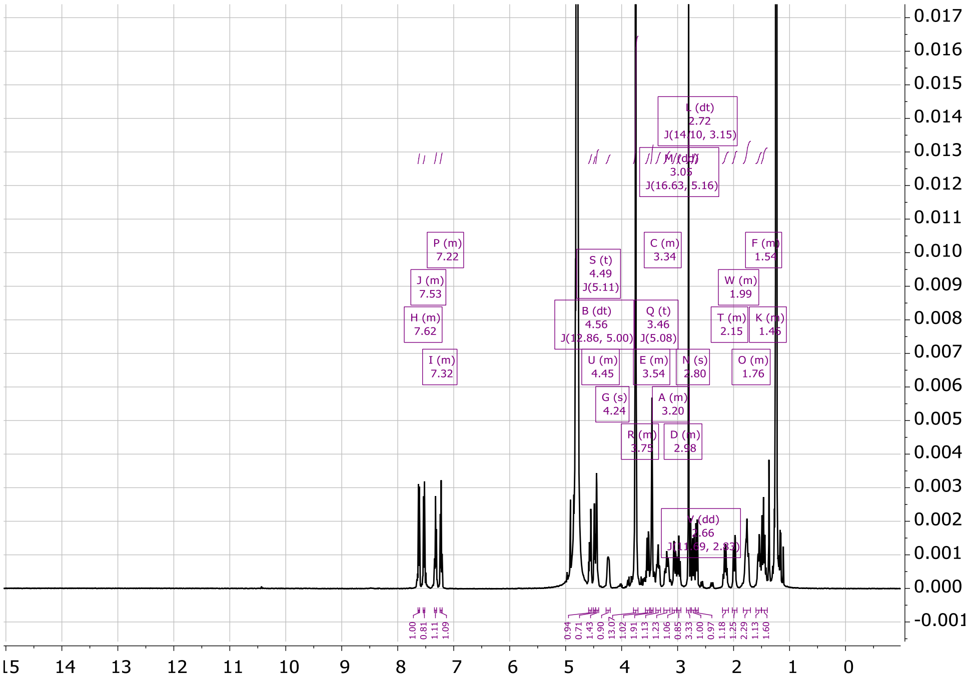

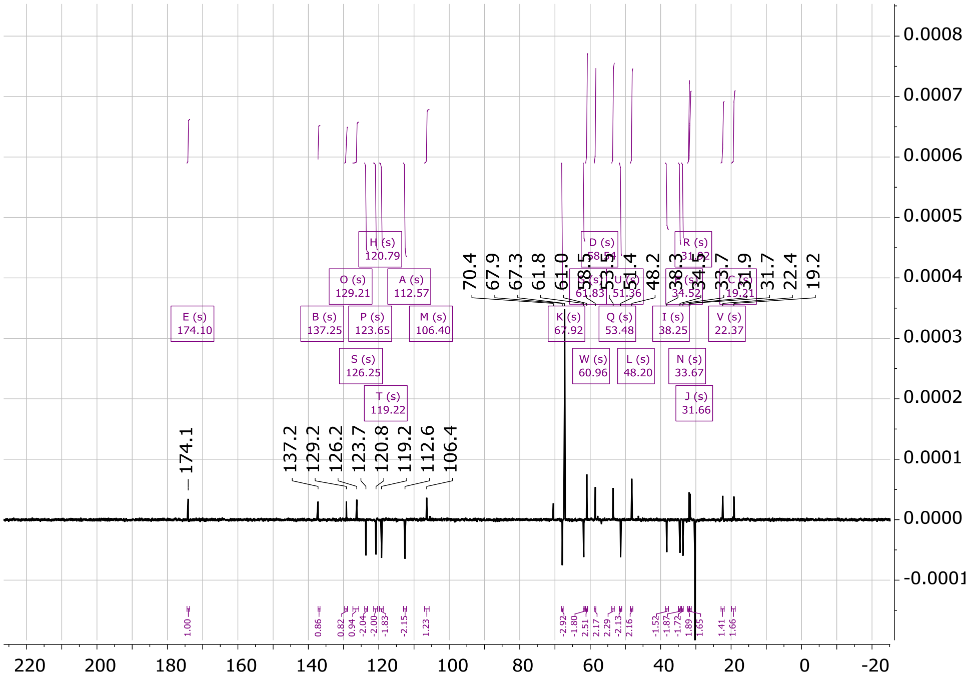

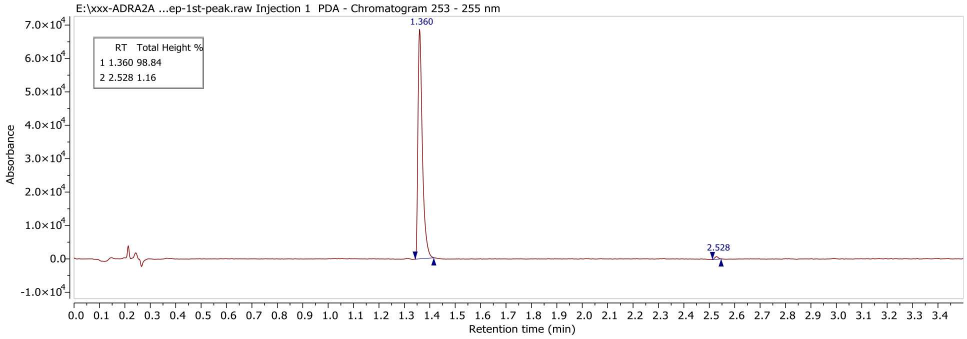

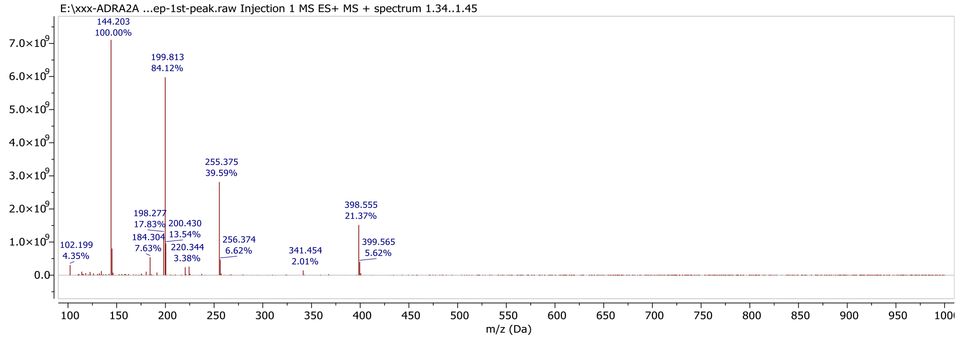

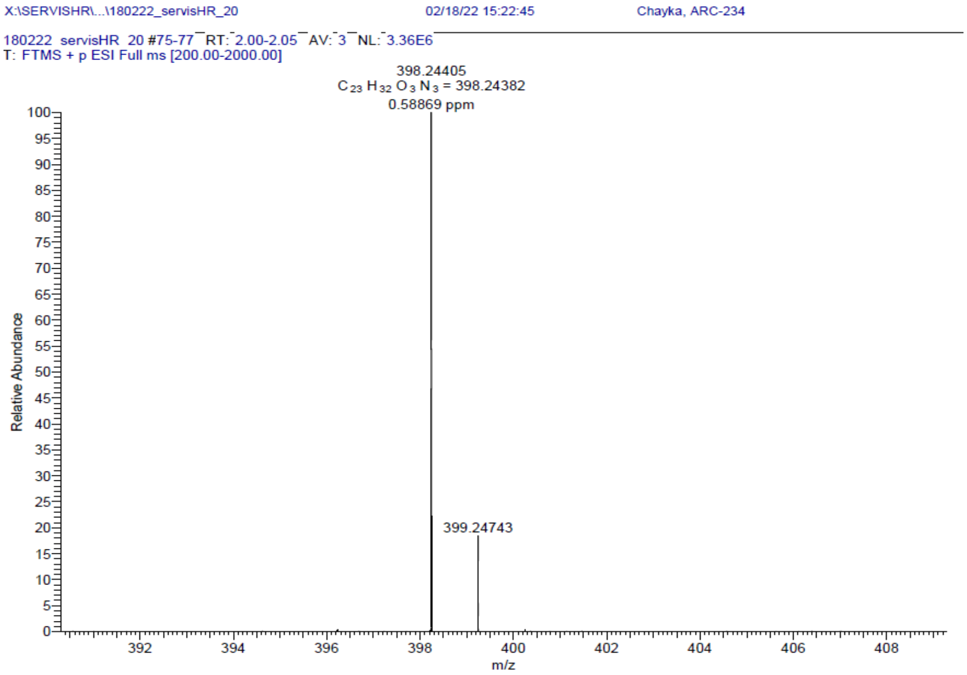

HRMS (ESI+): *m/z* $[M + H]^+$ calculated for $C_{23}H_{32}O_3N_3$ = 398.2438, found: 398.2441.

17-α-Hydroxyyohimban-16-α-carboxylic acid piperidine-4-yl ester (4d)

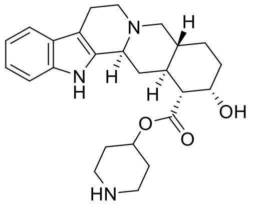

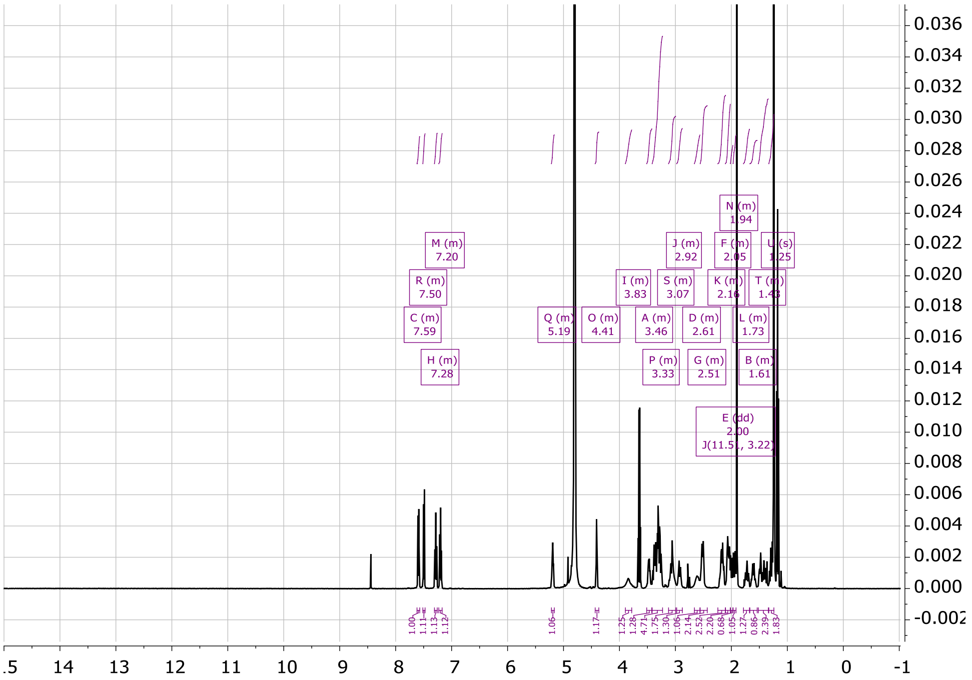

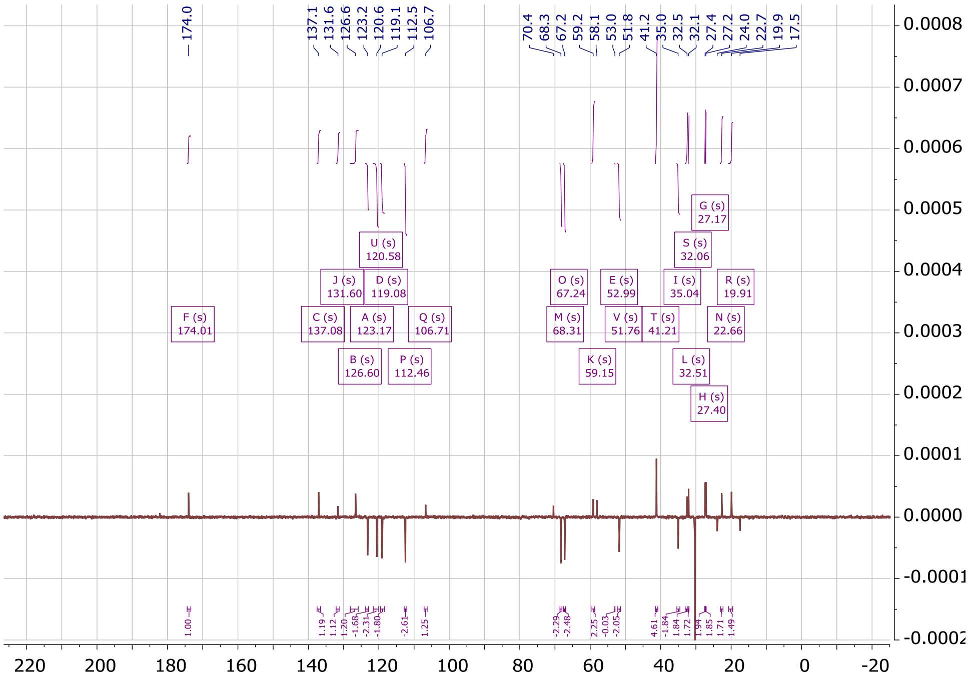

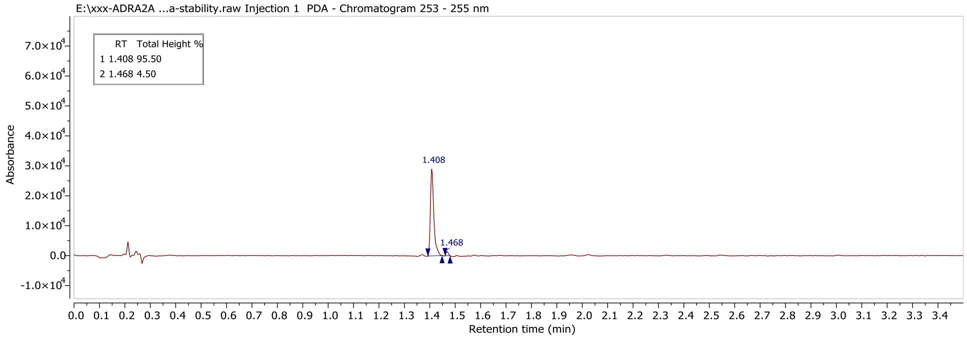

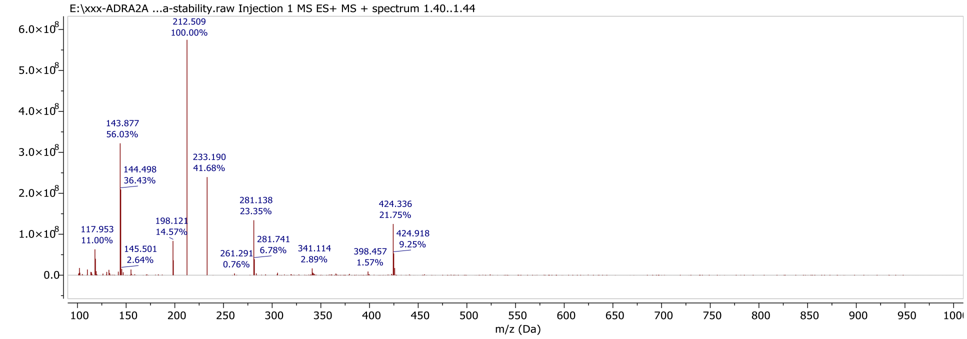

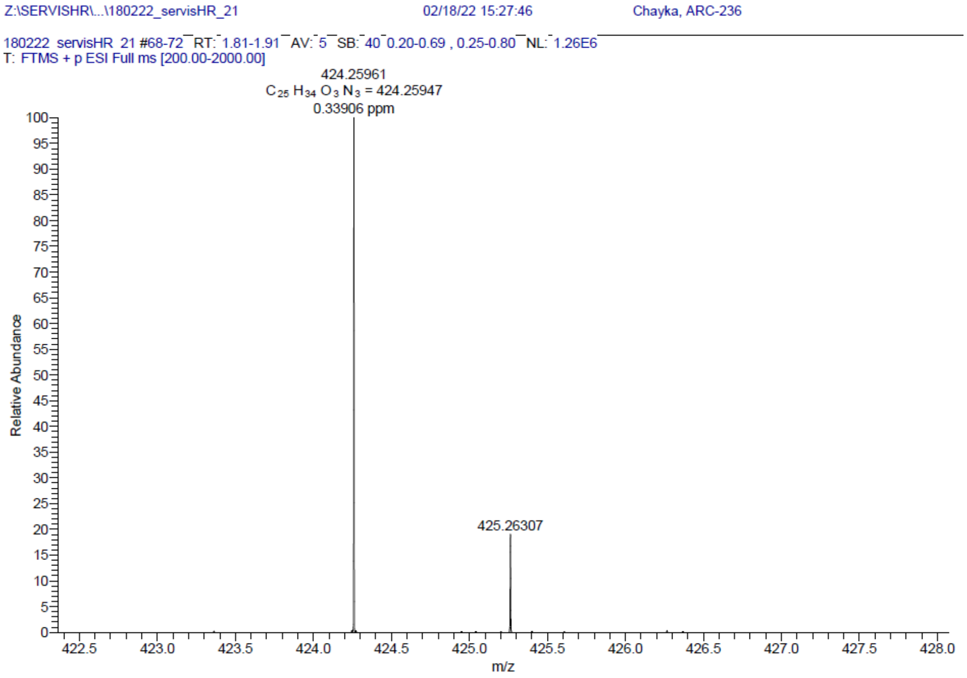

HRMS (ESI+): m/z $[M + H]^+$ calculated for $C_{25}H_{34}O_3N_3$ = 424.2595, found: 424.2596.

17-α-Hydroxyyohimban-16-α-carboxylic acid piperidine-3-yl ester (4e)

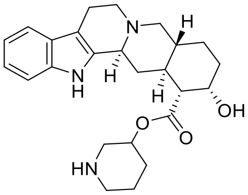

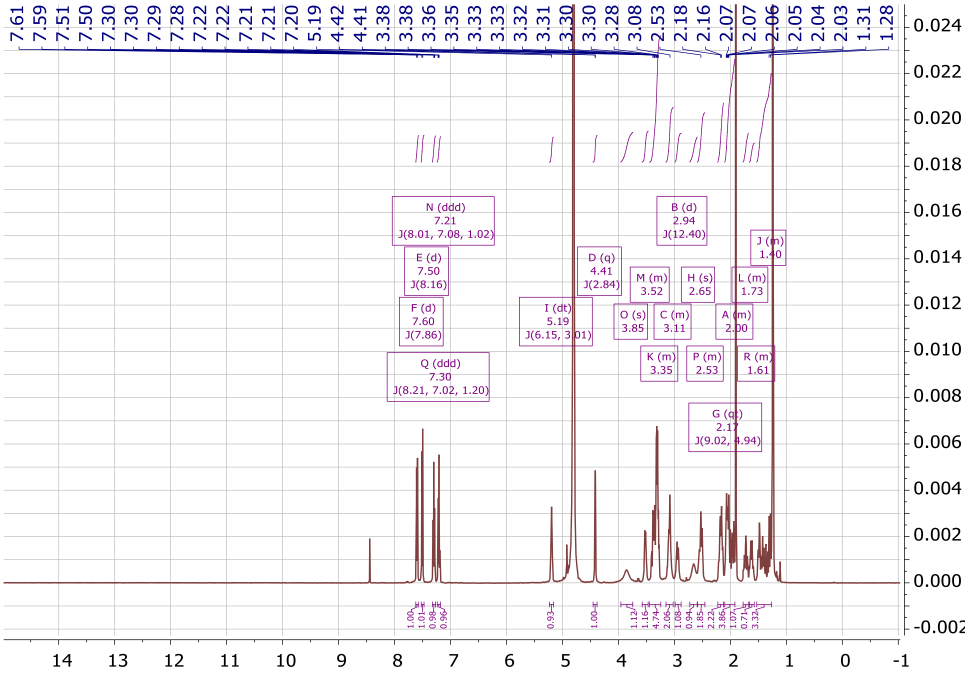

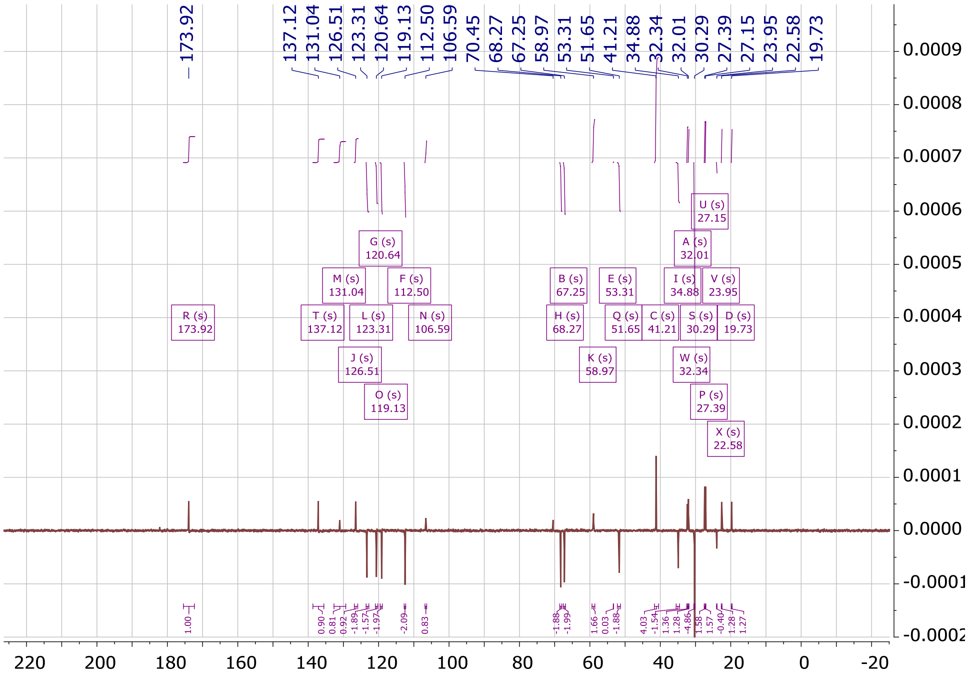

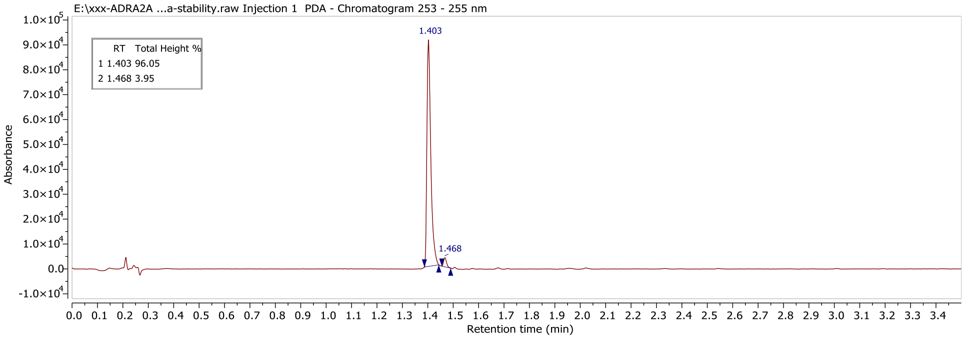

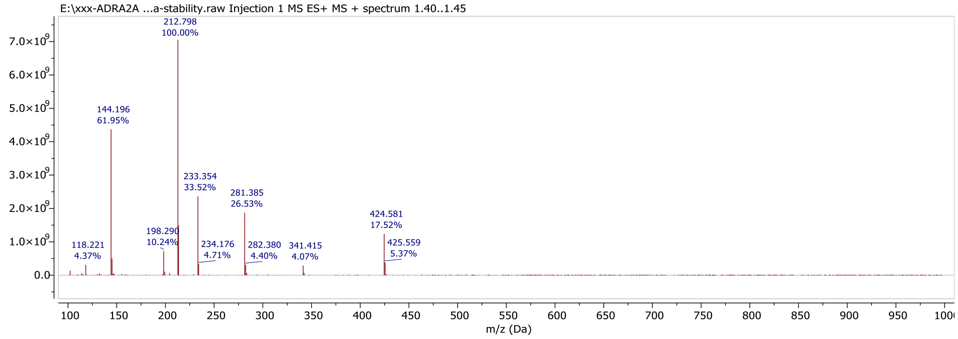

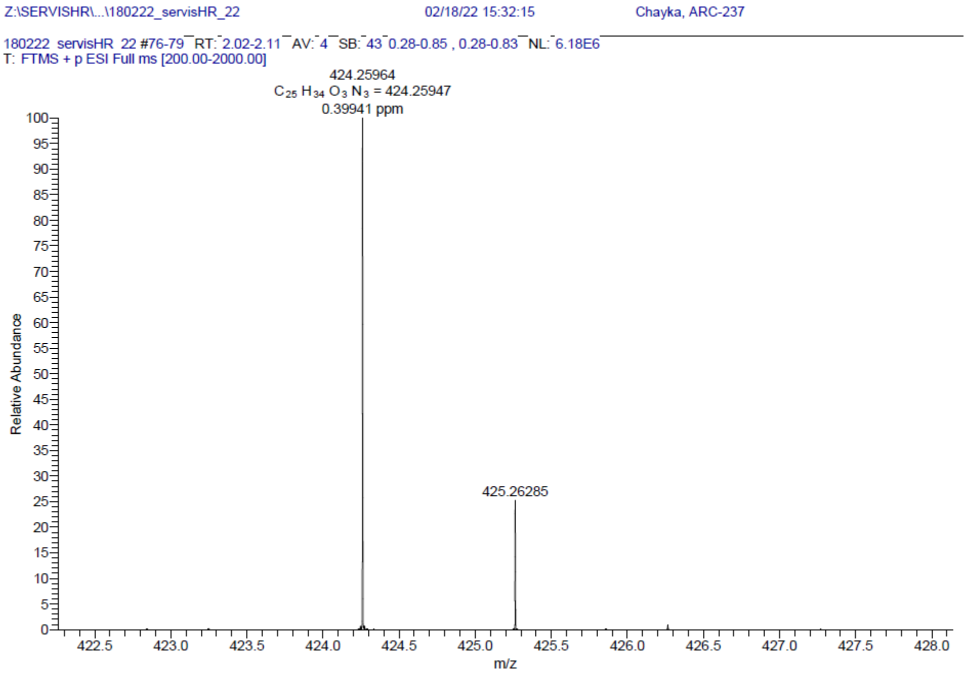

HRMS (ESI+): *m/z* [M + H]$^+$ calculated for $C_{25}H_{34}O_3N_3$ = 424.2595, found: 424.2596.

17-α-Hydroxyyohimban-16-α-carboxylic acid azetidin-3-ylmethyl ester (4f)

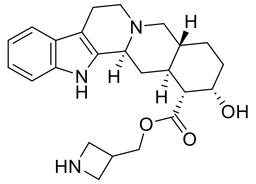

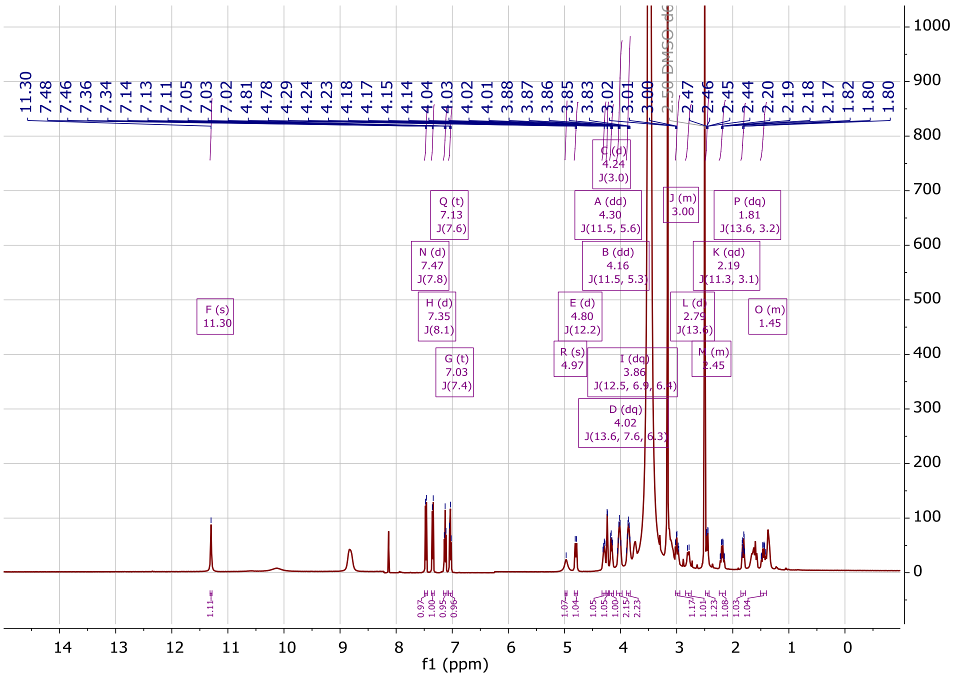

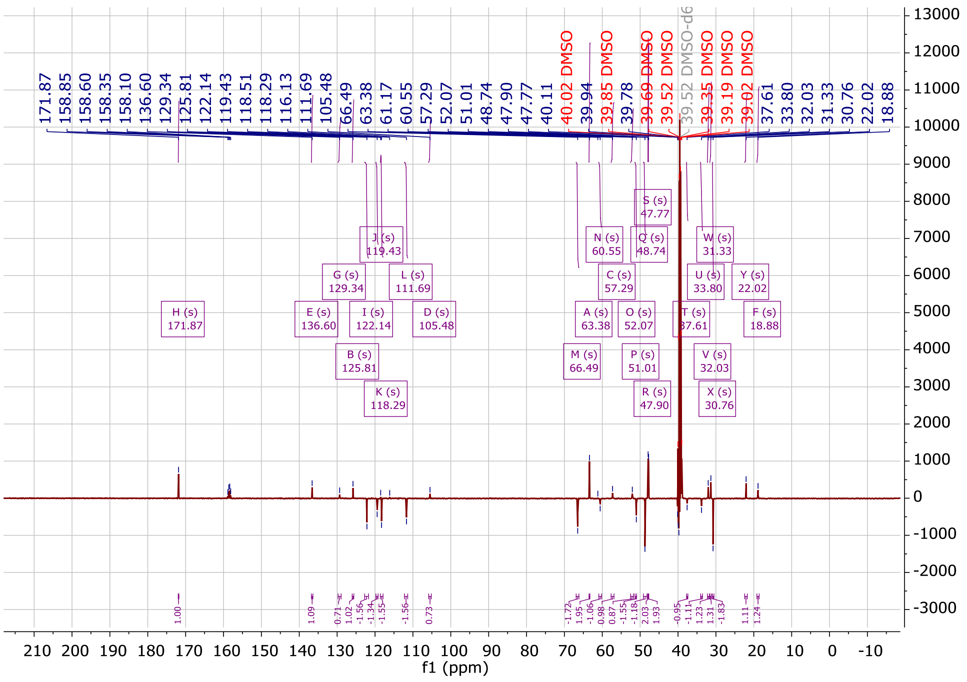

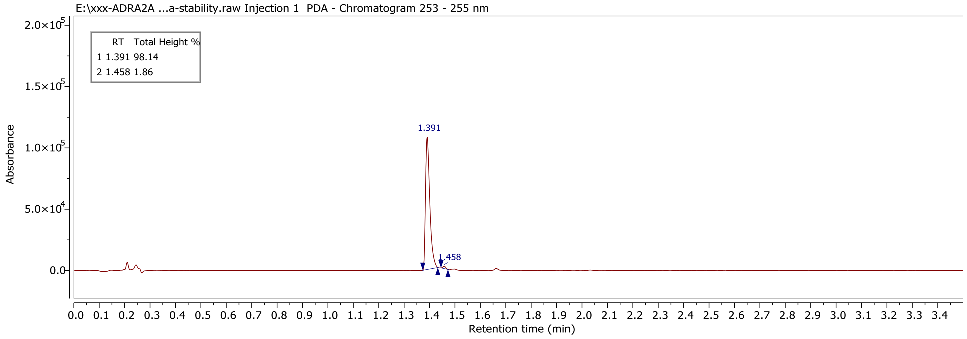

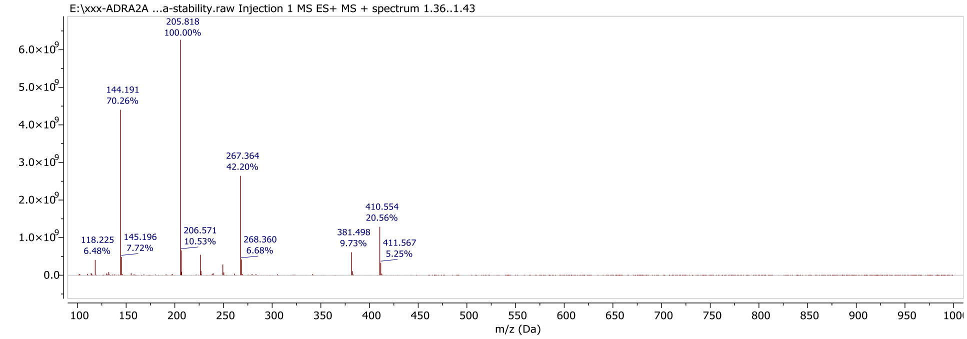

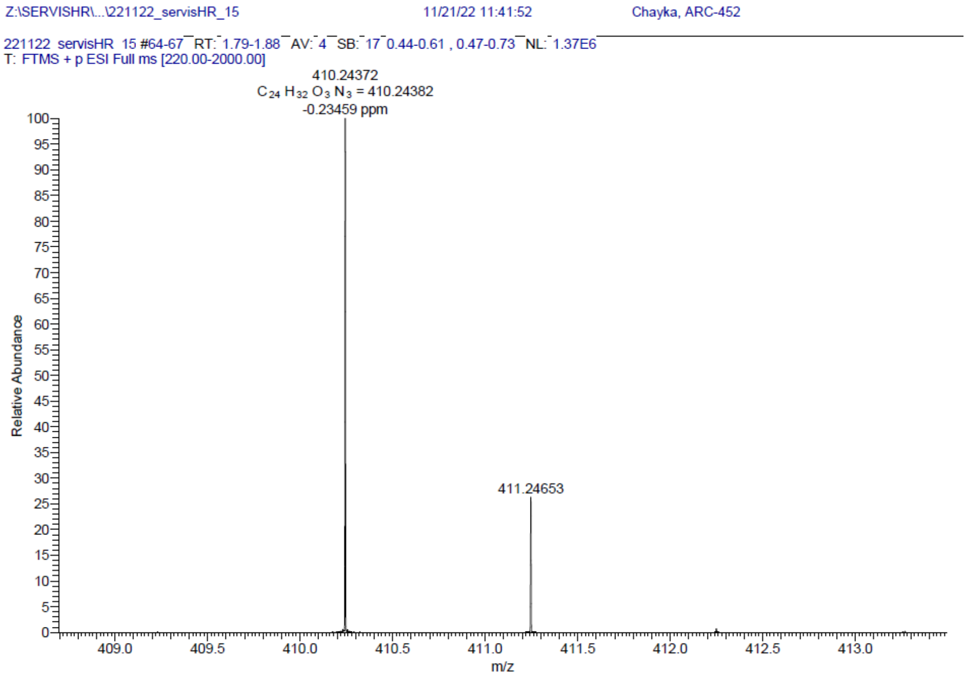

HRMS (ESI+): *m/z* $[M + H]^+$ calculated for $C_{24}H_{32}O_3N_3$ = 410.2438, found: 410.2437.

17-α-Hydroxyyohimban-16-α-carboxylic acid (*R*)-azetidin-2-ylmethyl ester (4g)

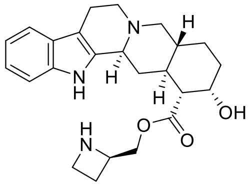

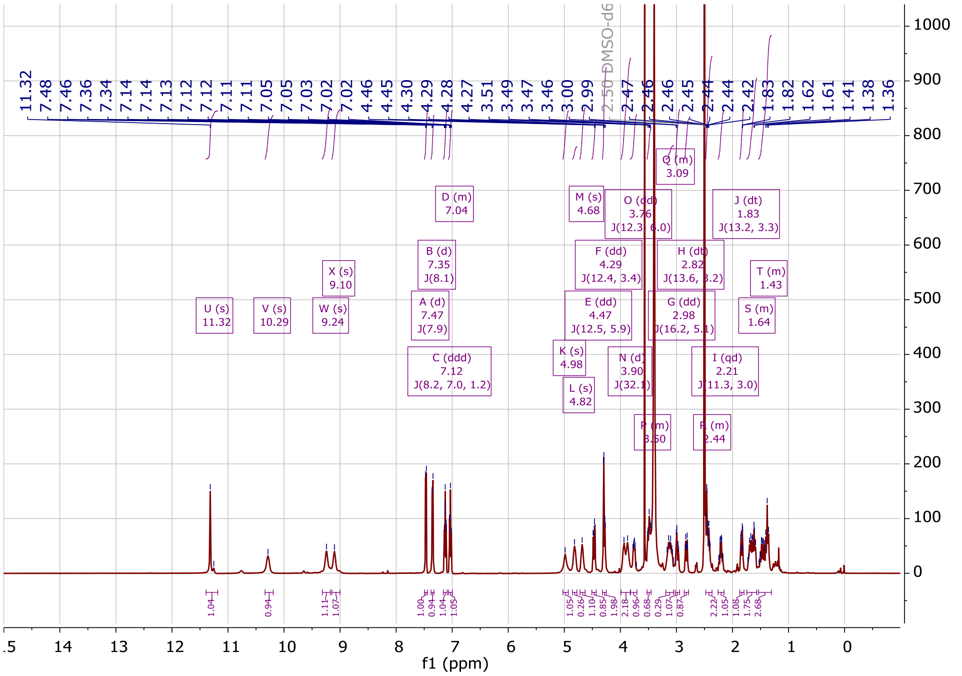

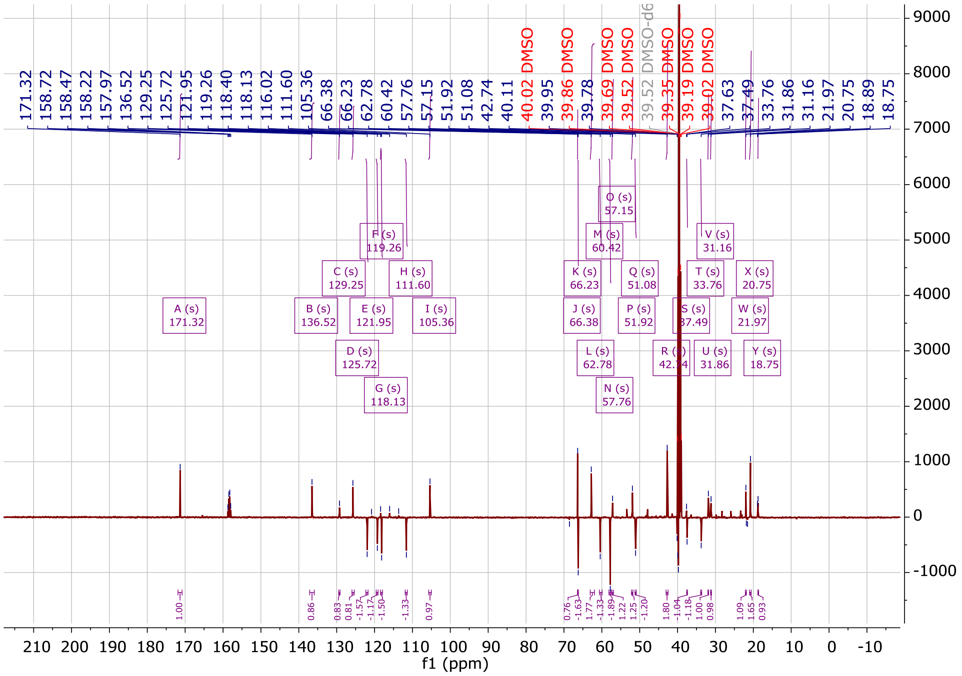

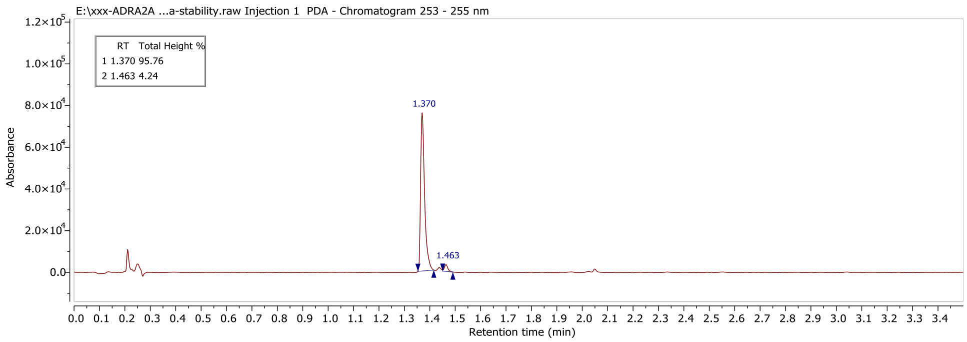

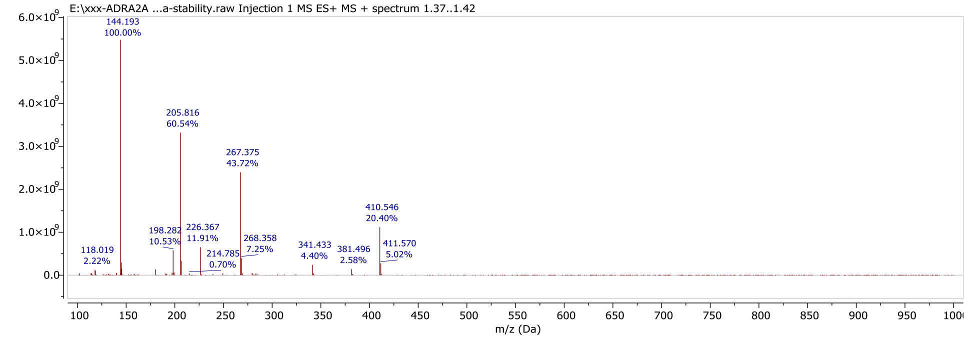

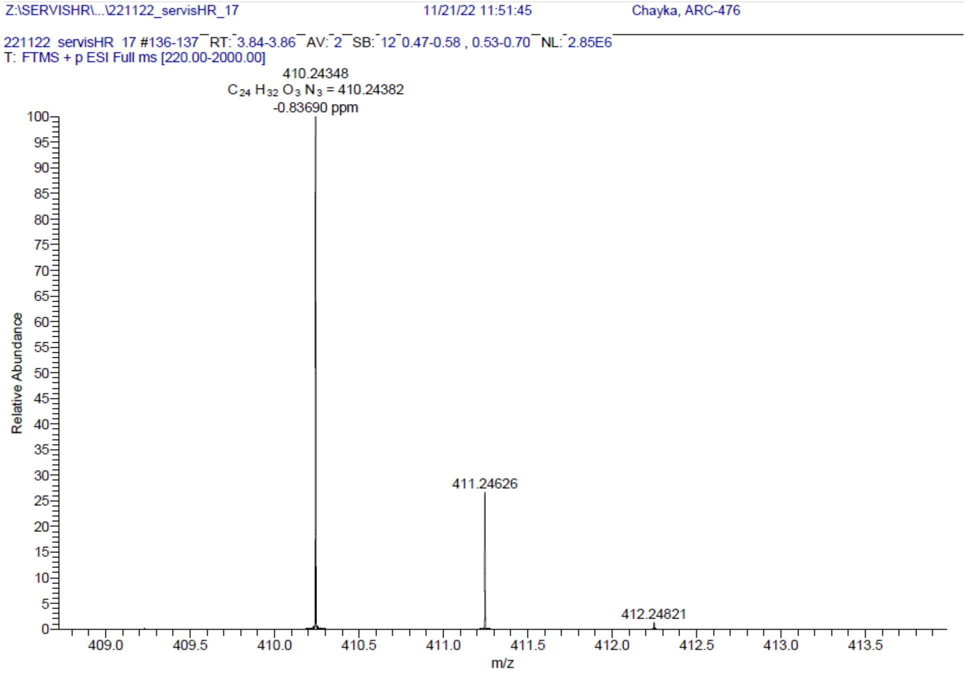

HRMS (ESI+): *m/z* $[M + H]^+$ calculated for $C_{24}H_{32}O_3N_3$ = 410.2438, found: 410.2435.

17-α-Hydroxyyohimban-16-α-carboxylic acid (*S*)-azetidin-2-ylmethyl ester (4h)

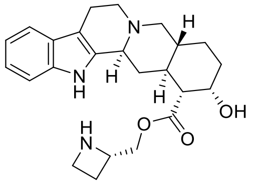

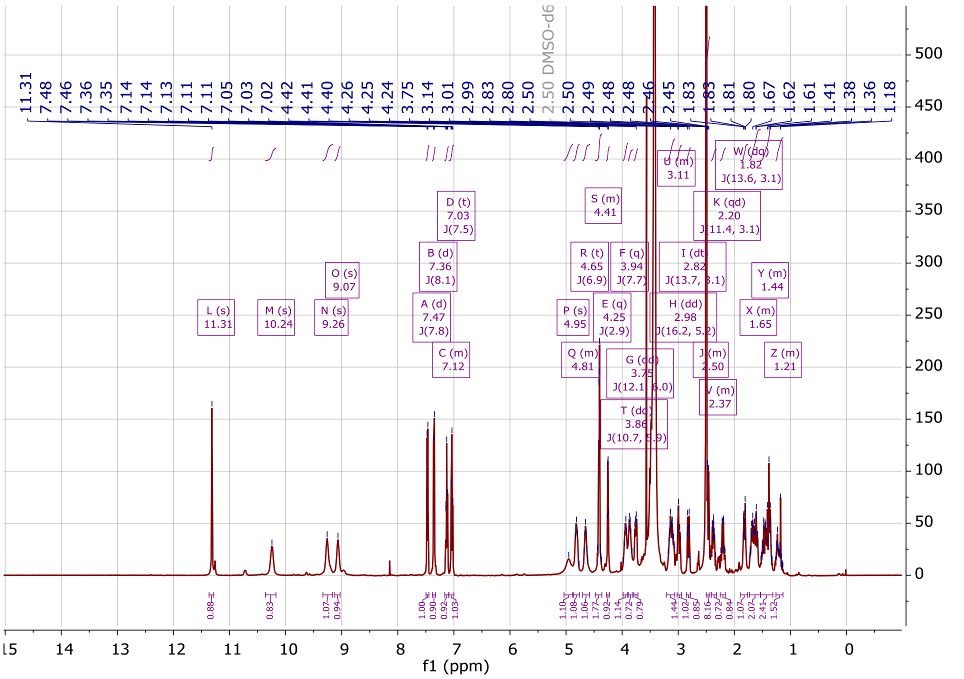

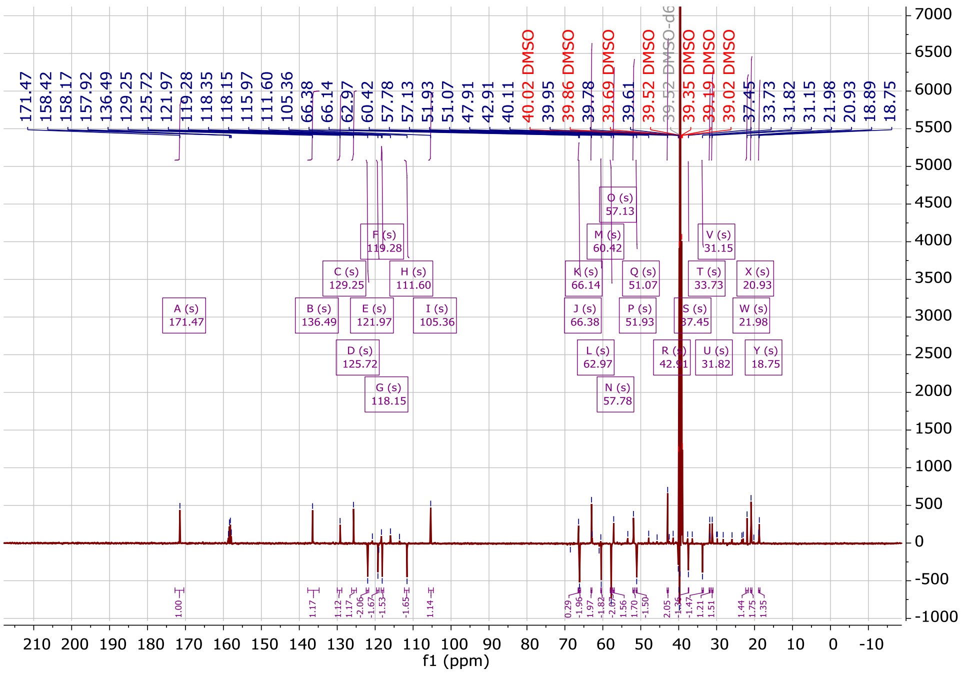

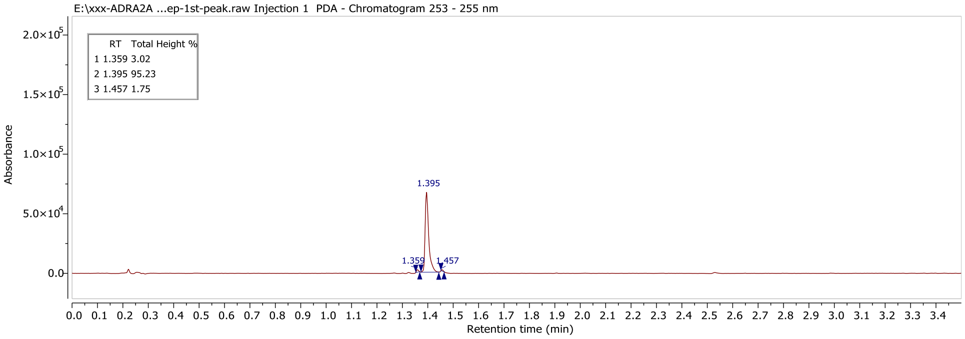

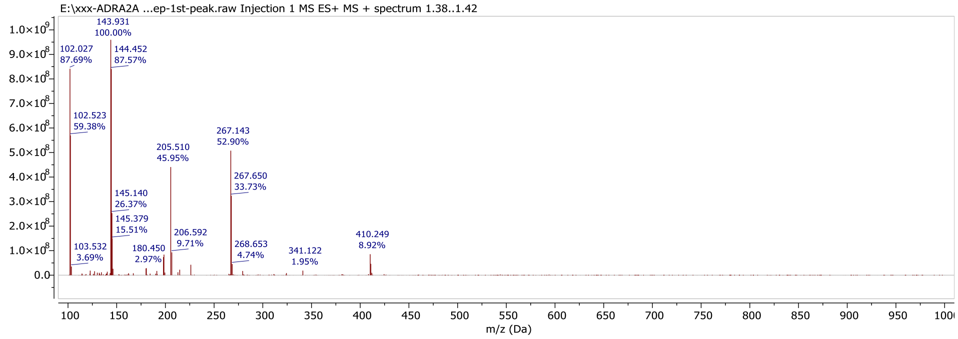

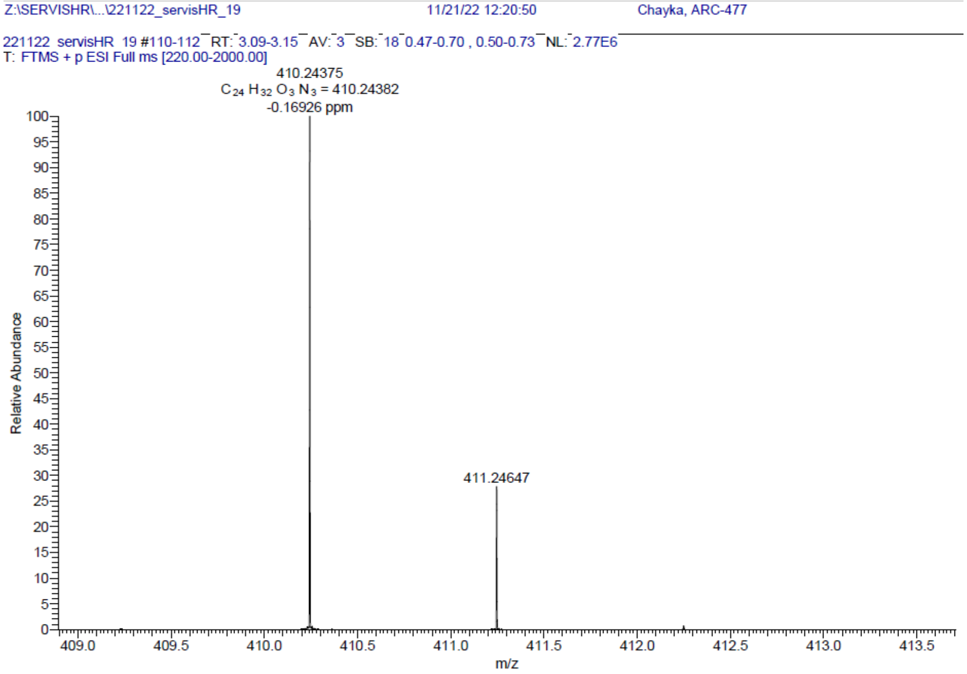

HRMS (ESI+): *m/z* [M + H]$^+$ calculated for $C_{24}H_{32}O_3N_3$ = 410.2438, found: 410.2438.

17-α-Hydroxyyohimban-16-α-carboxylic acid (*R*)-pyrrolidin-3-ylmethyl ester (4i)

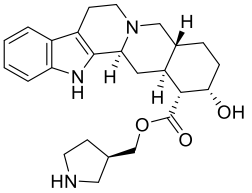

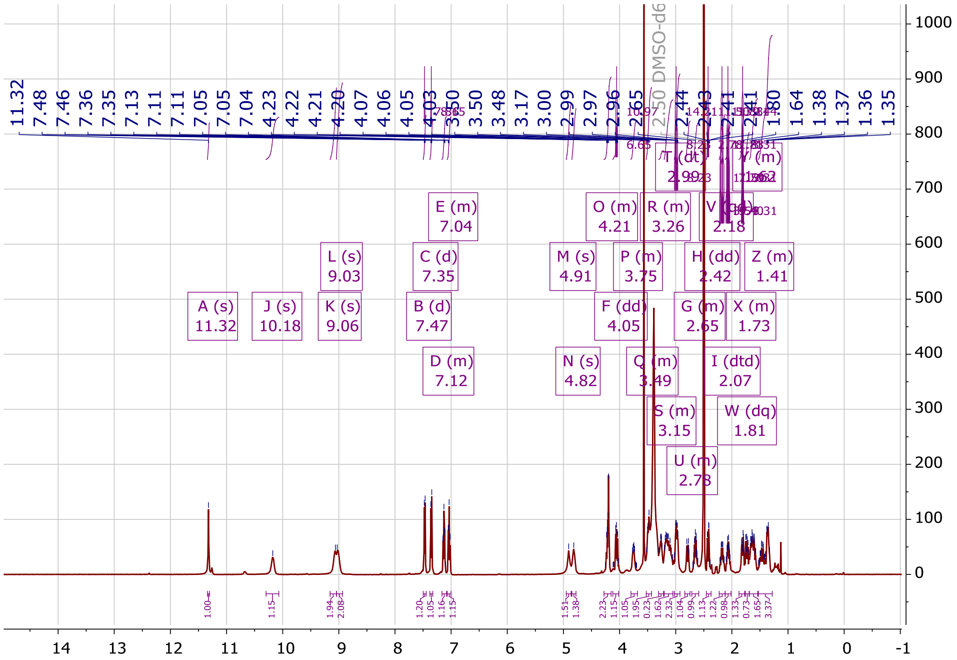

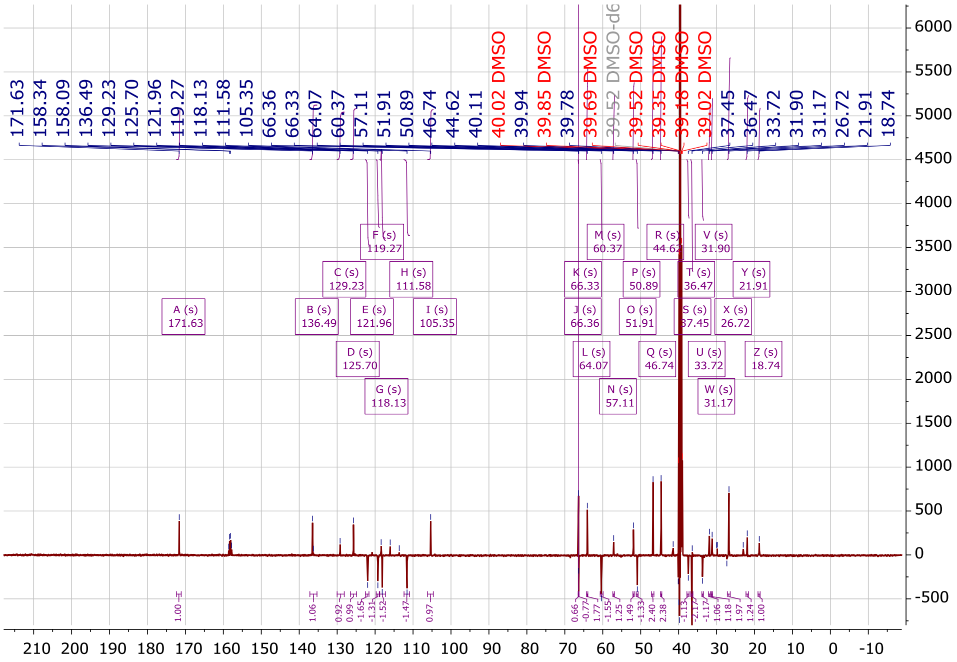

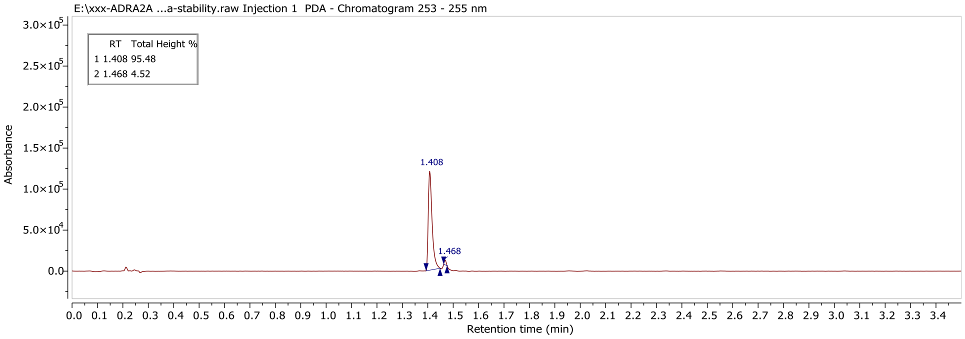

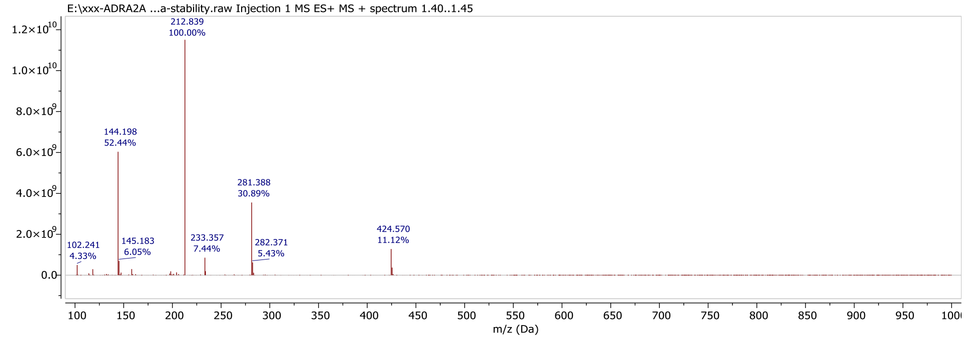

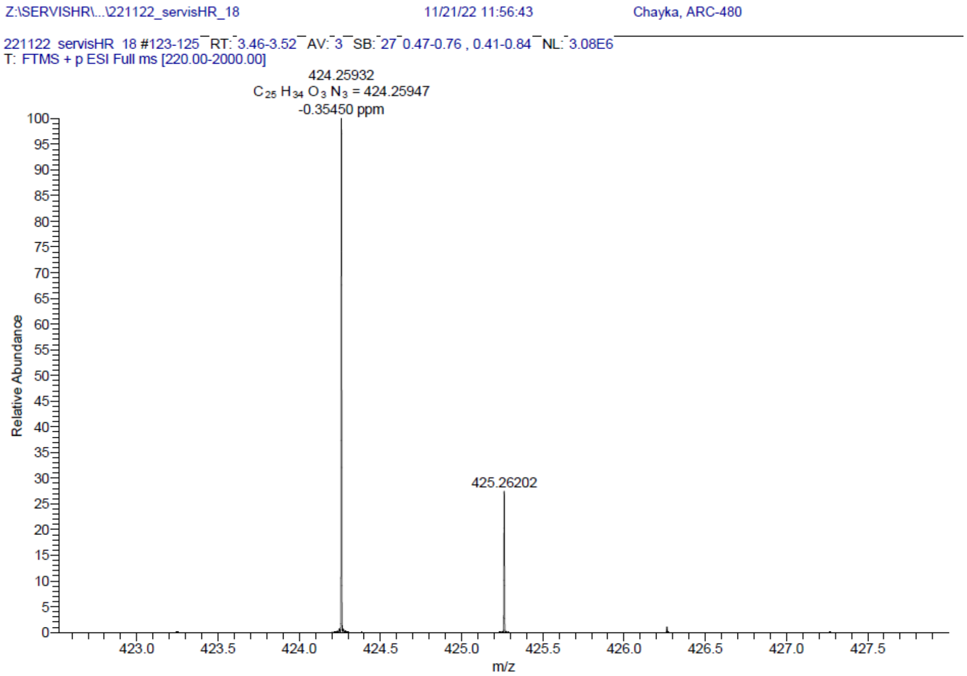

HRMS (ESI+): *m/z* $[M + H]^+$ calculated for $C_{25}H_{34}O_3N_3$ = 424.2595, found: 424.2593.

17-α-Hydroxyyohimban-16-α-carboxylic acid (*S*)-pyrrolidin-3-ylmethyl ester (4j)

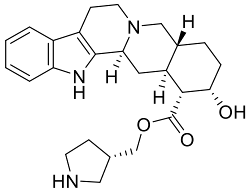

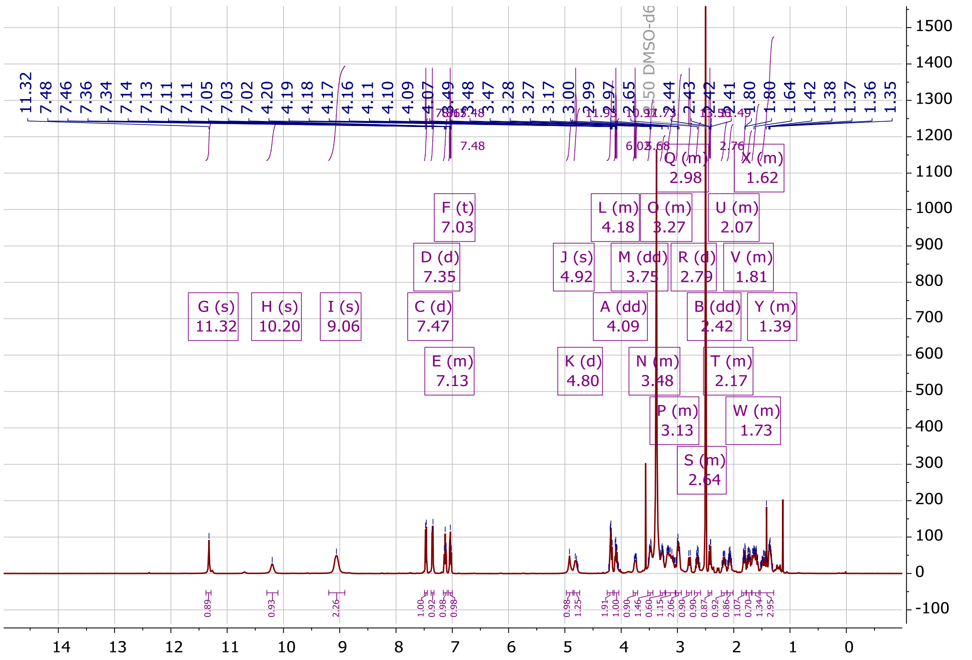

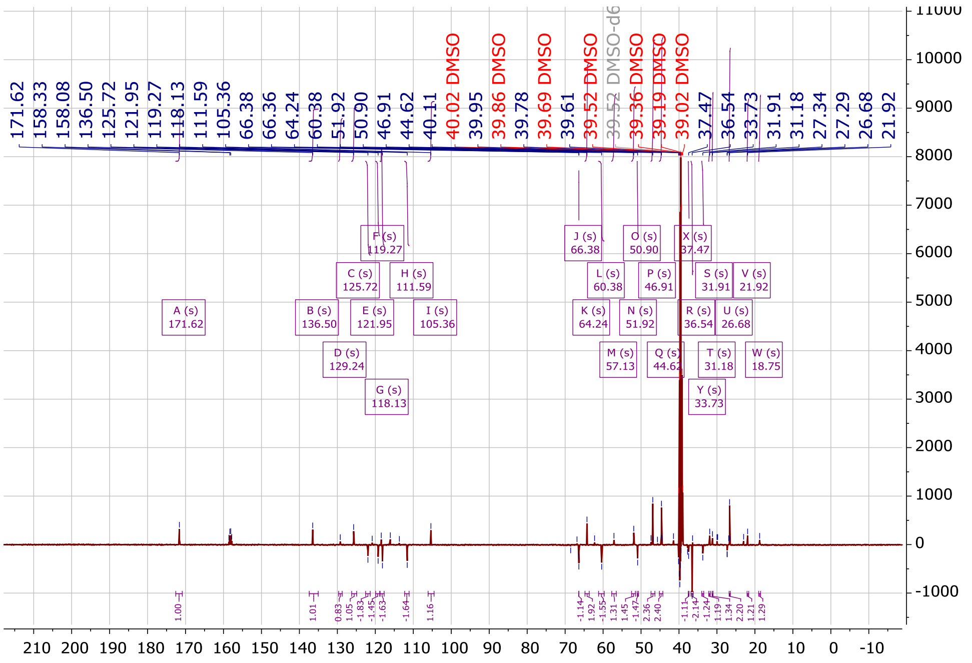

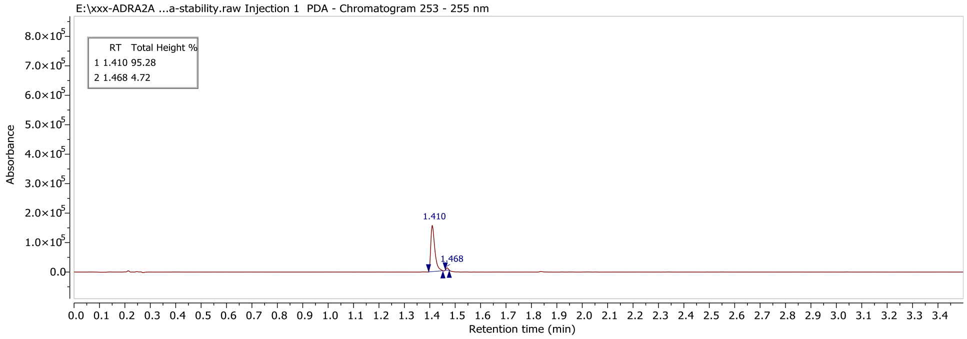

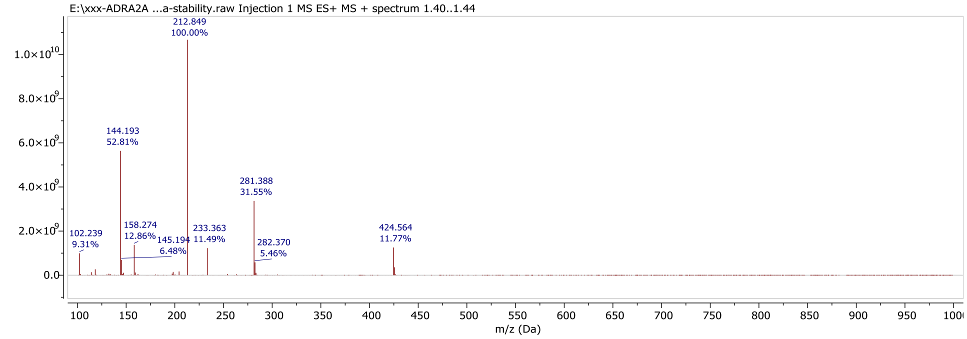

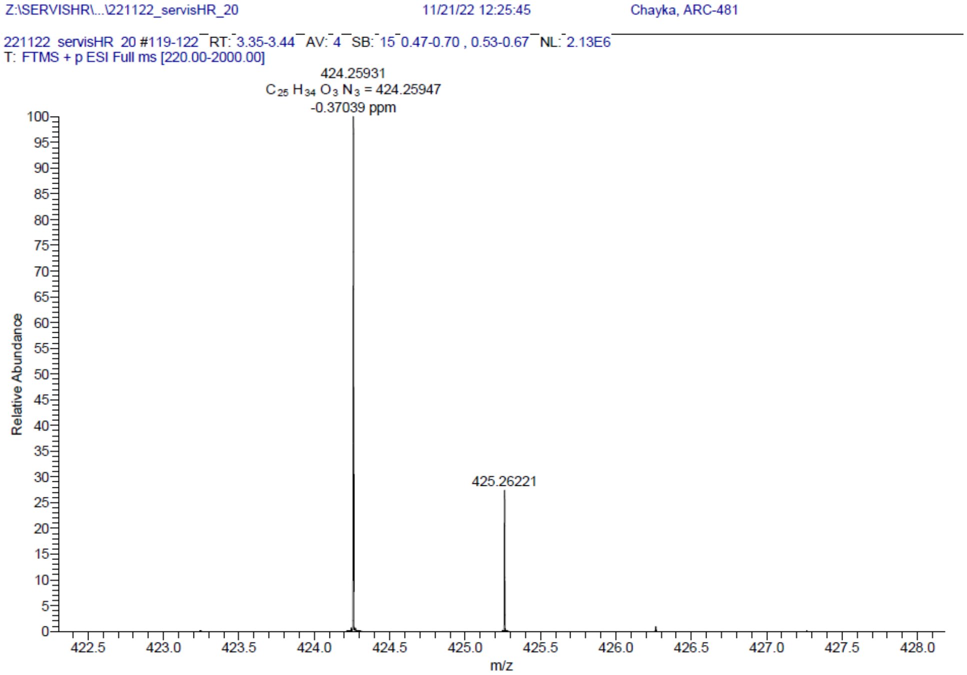

HRMS (ESI+): *m/z* $[M + H]^+$ calculated for $C_{25}H_{34}O_3N_3$ = 424.2595, found: 424.2593.

17-α-Hydroxyyohimban-16-α-carboxylic acid (*R*)-pyrrolidin-3-yl ester (4k)

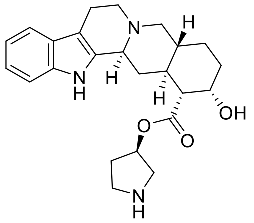

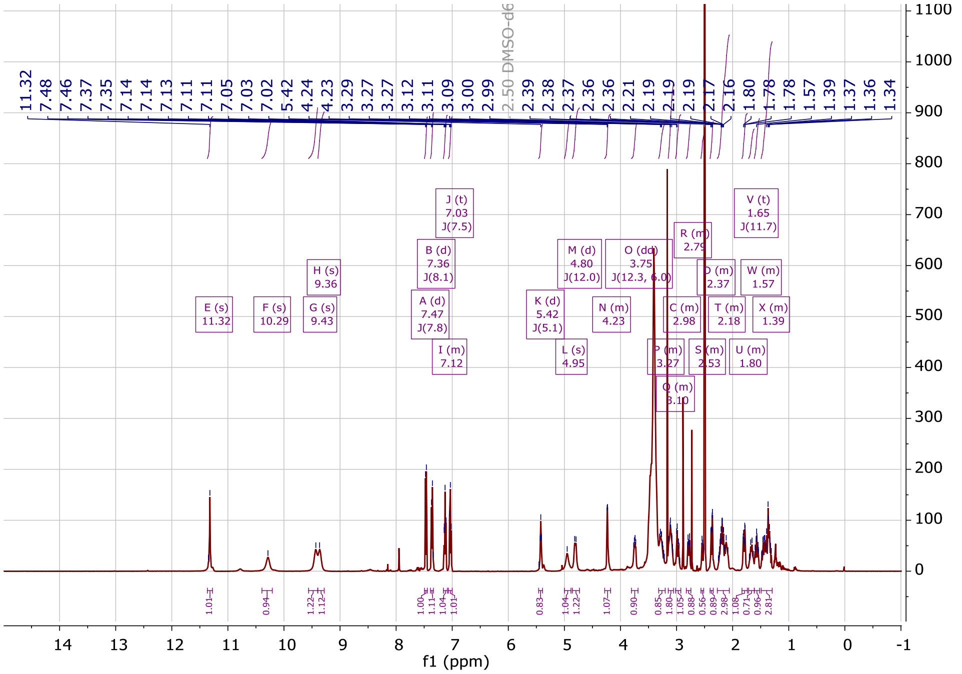

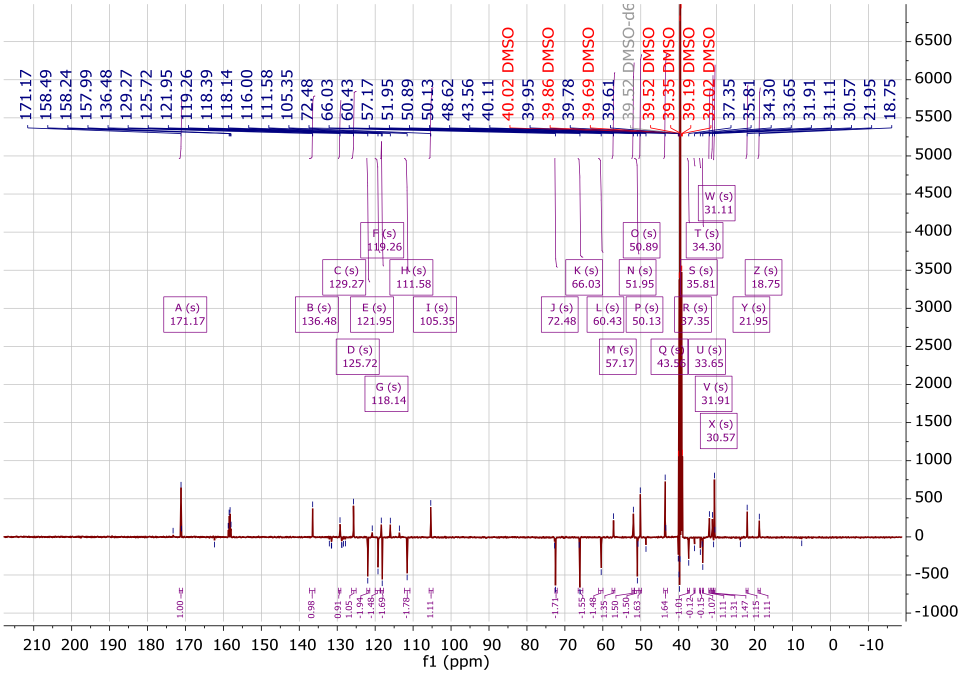

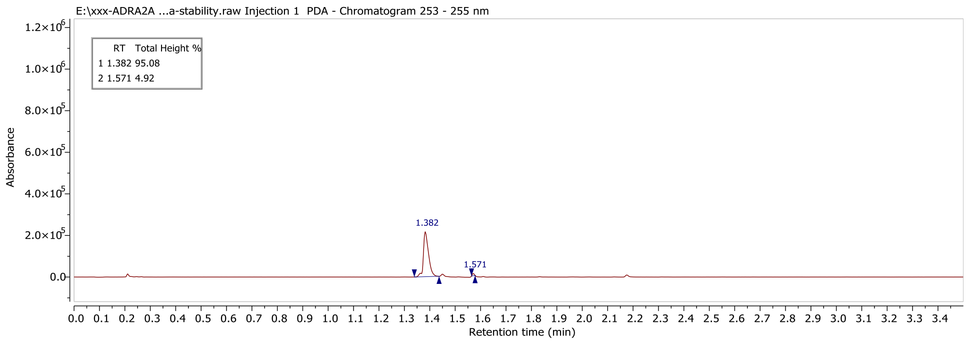

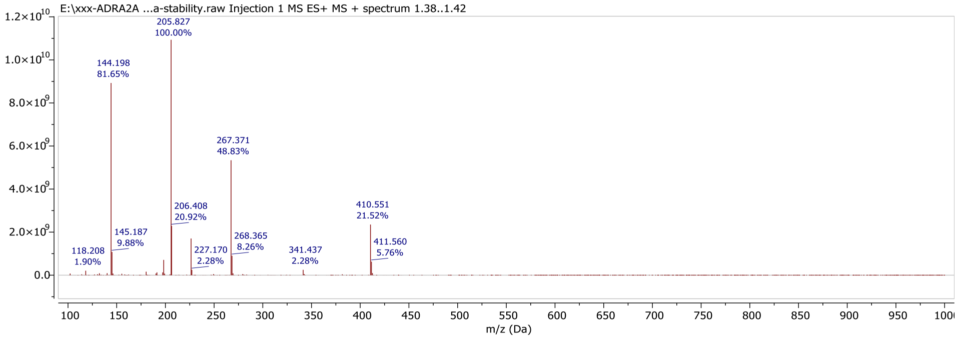

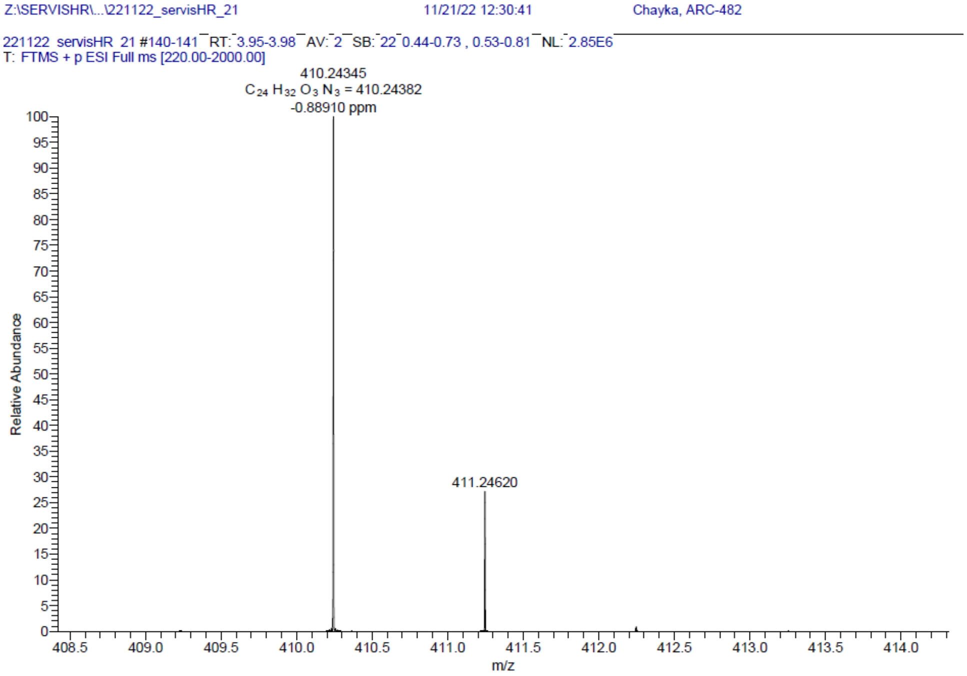

HRMS (ESI+): *m/z* $[M + H]^+$ calculated for $C_{24}H_{32}O_3N_3$ = 410.2438, found: 410.2435.

17-α-Hydroxyyohimban-16-α-carboxylic acid (*S*)-pyrrolidin-3-yl ester (4l)

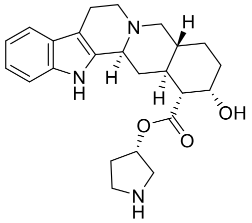

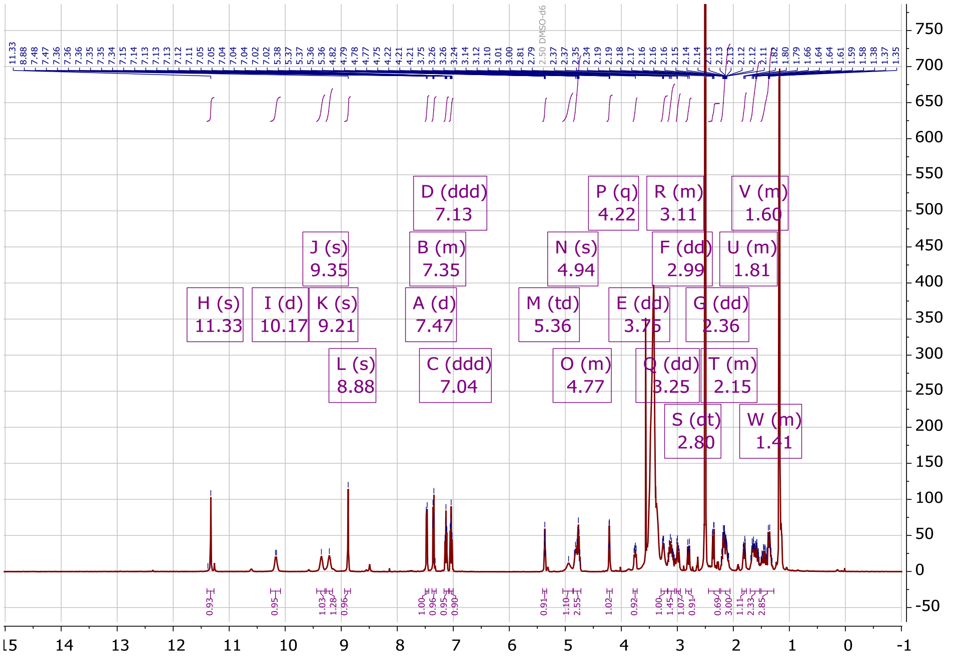

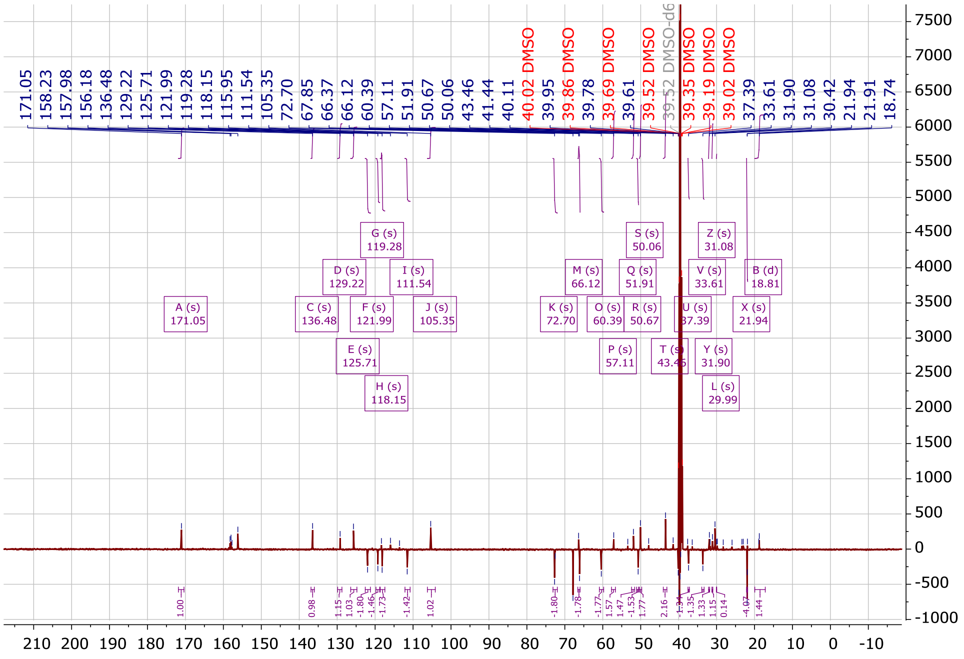

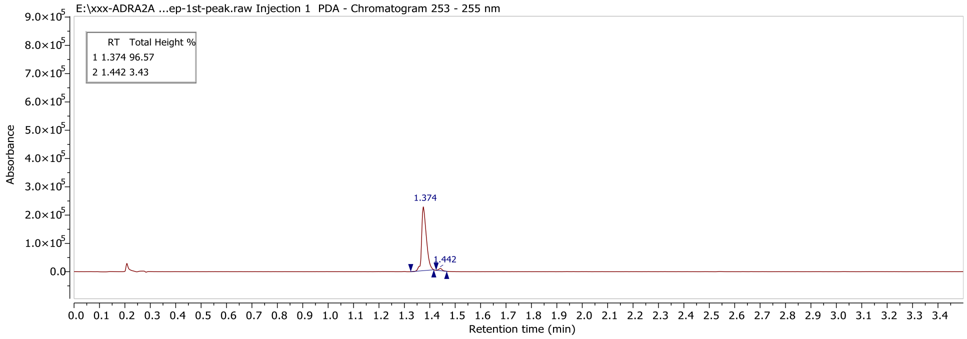

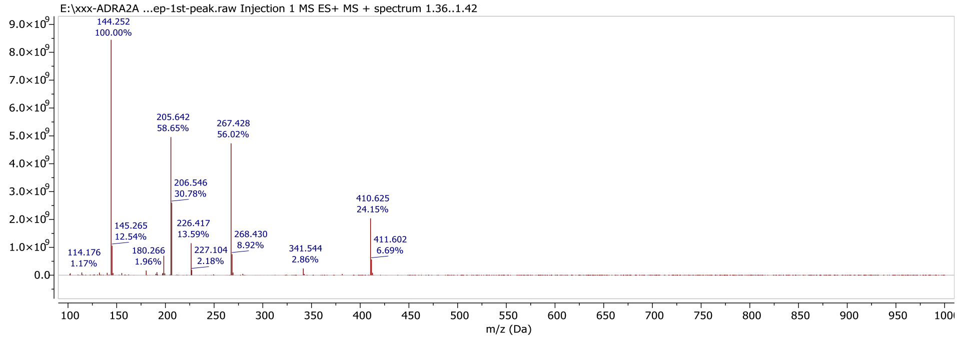

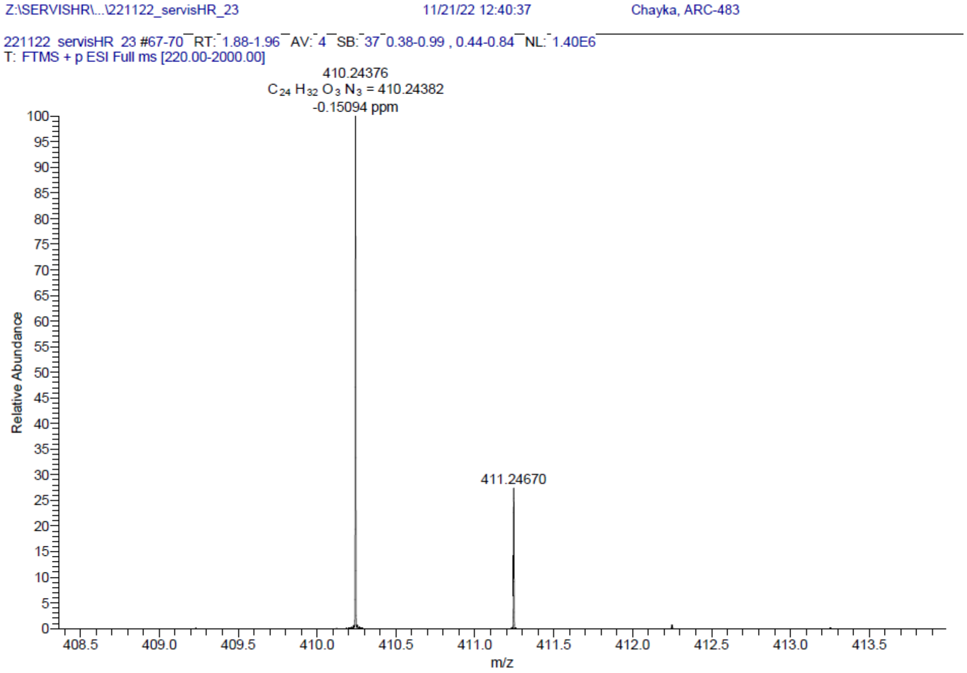

HRMS (ESI+): *m/z* $[M + H]^+$ calculated for $C_{24}H_{32}O_3N_3$ = 410.2438, found: 410.2438.

17-α-Hydroxyyohimban-16-α-carboxylic acid azetidin-3-yl ester (4m)

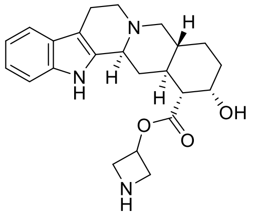

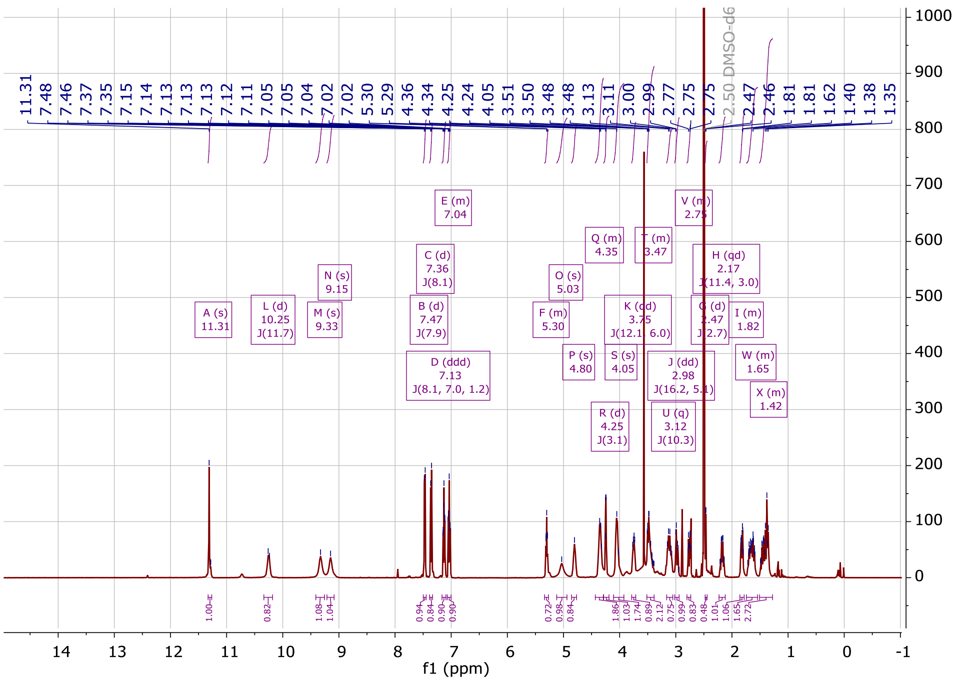

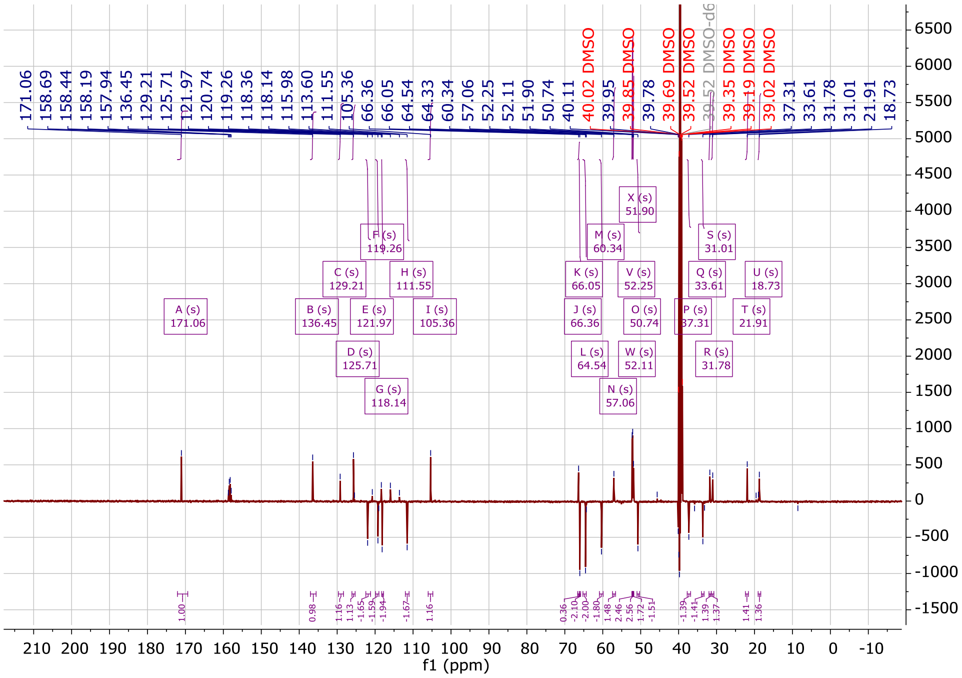

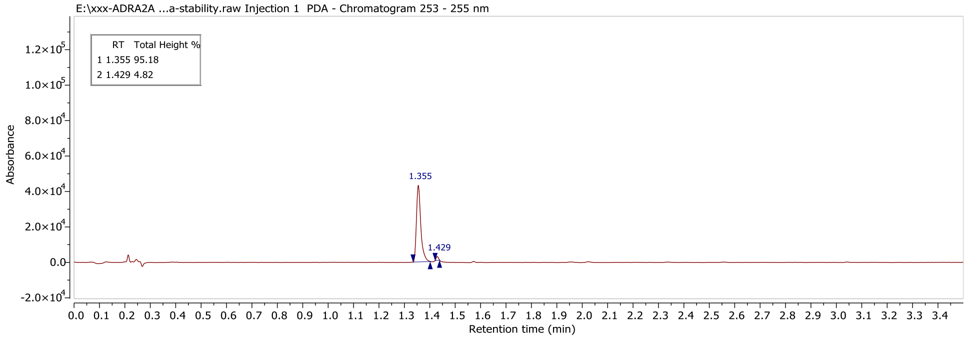

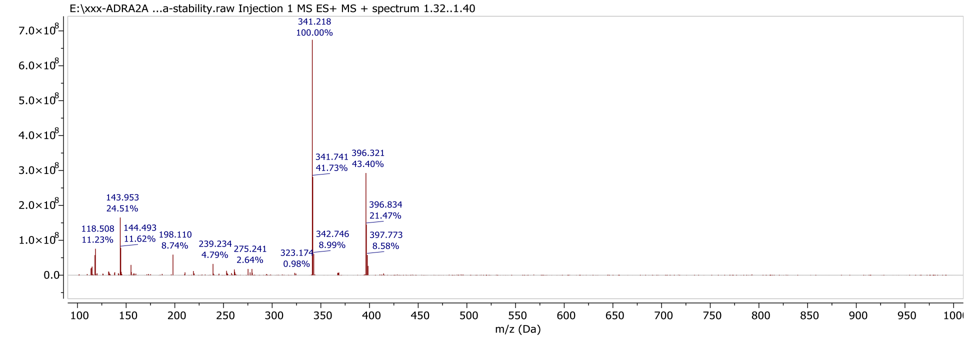

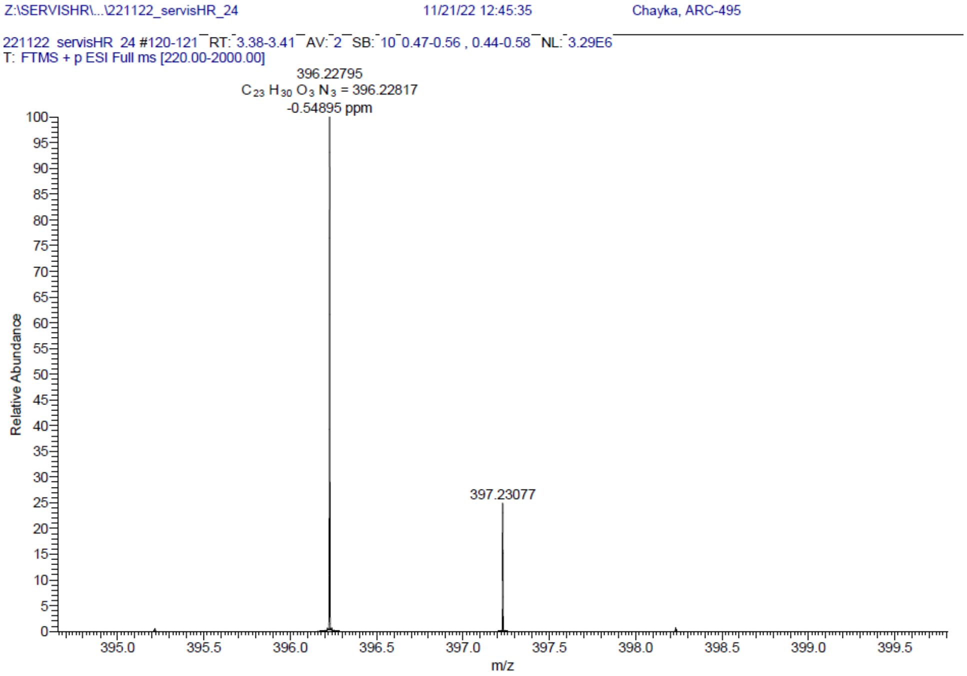

HRMS (ESI+): *m/z* $[M + H]^+$ calculated for $C_{24}H_{32}O_3N_3$ = 396.2282, found: 396.2280.

17-α-Hydroxyyohimban-16-α-carboxylic acid *trans*-3-aminocyclobutyl ester (4n)

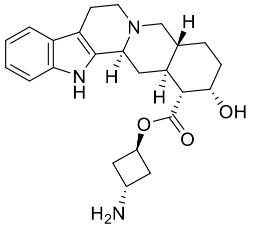

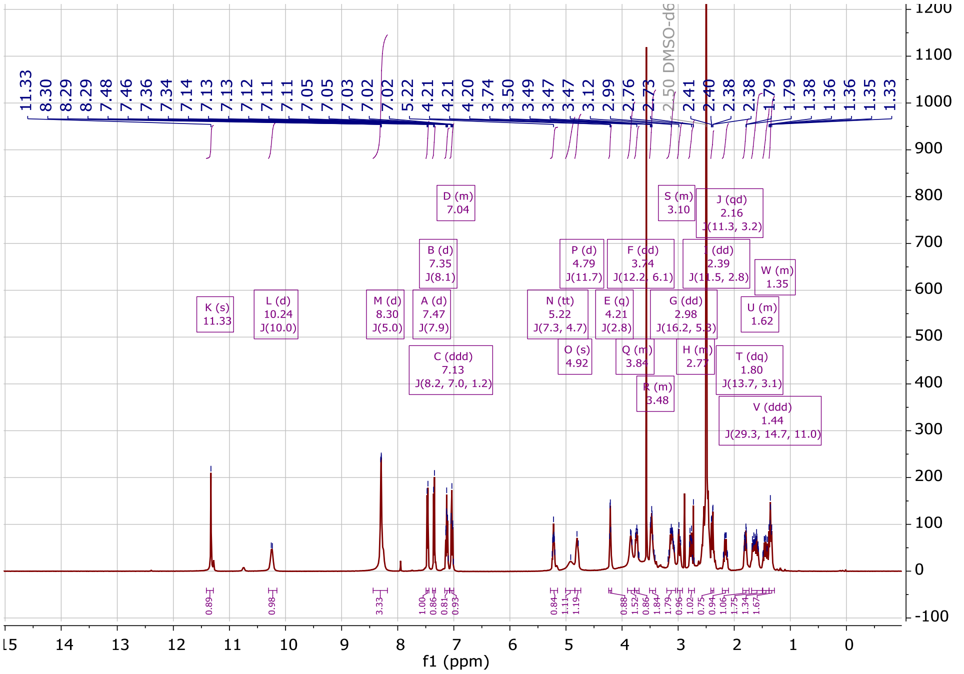

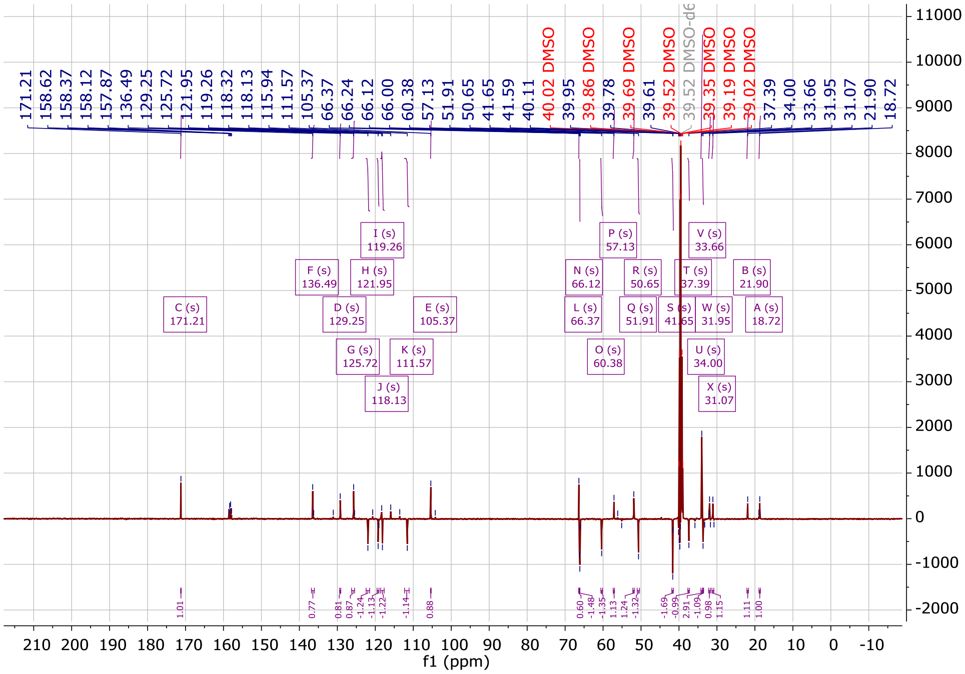

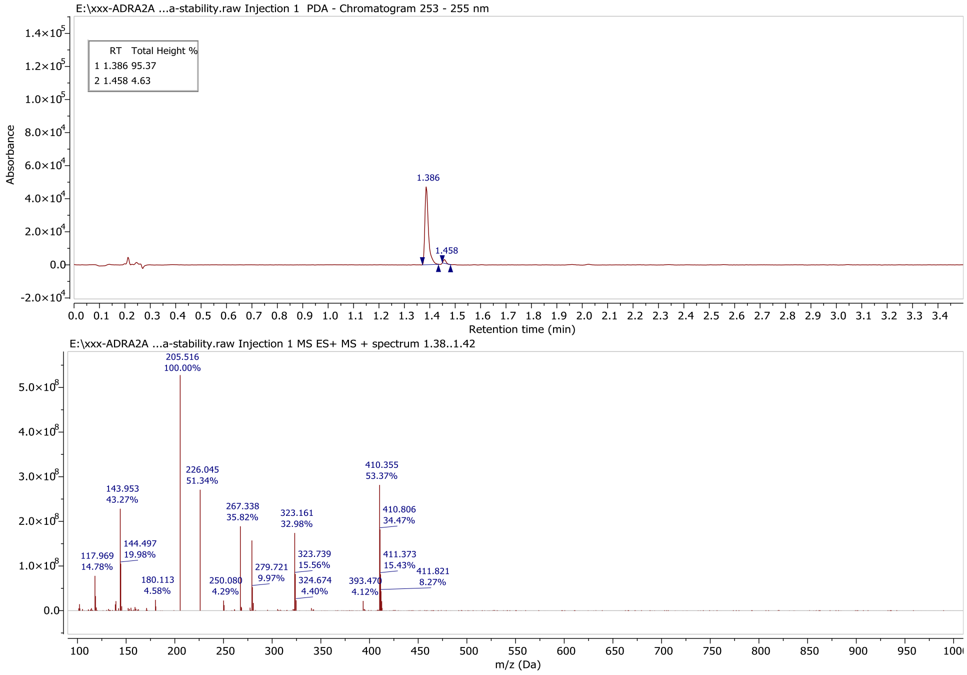

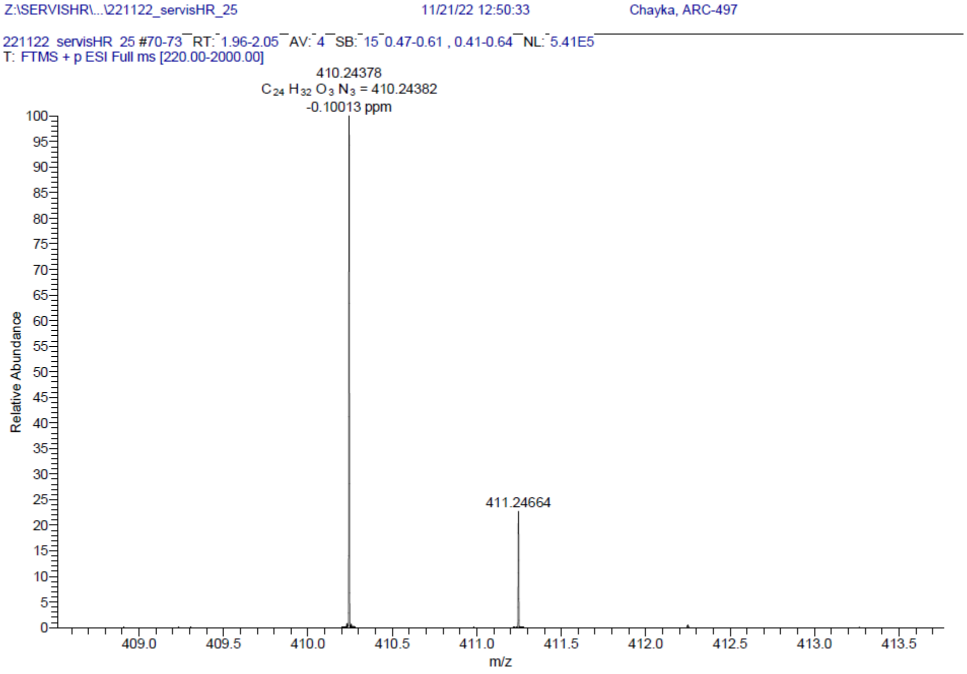

HRMS (ESI+): *m/z* [M + H]$^+$ calculated for $C_{24}H_{32}O_3N_3$ = 410.2438, found: 410.2438.

17-α-Hydroxyyohimban-16-α-carboxylic acid *cis*-3-aminocyclobutyl ester (4o)

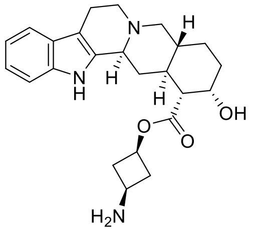

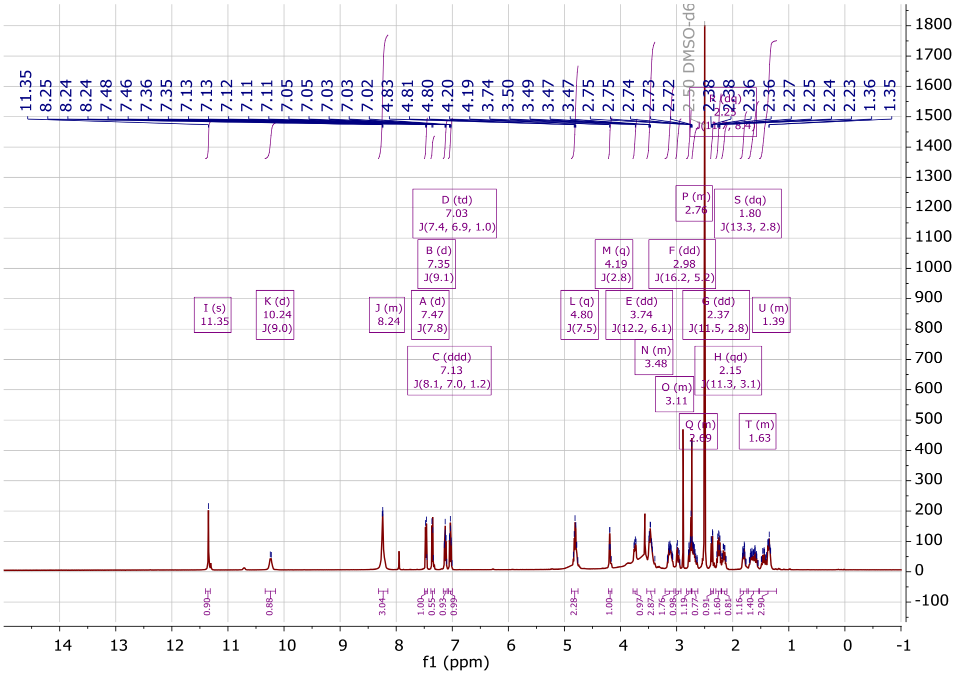

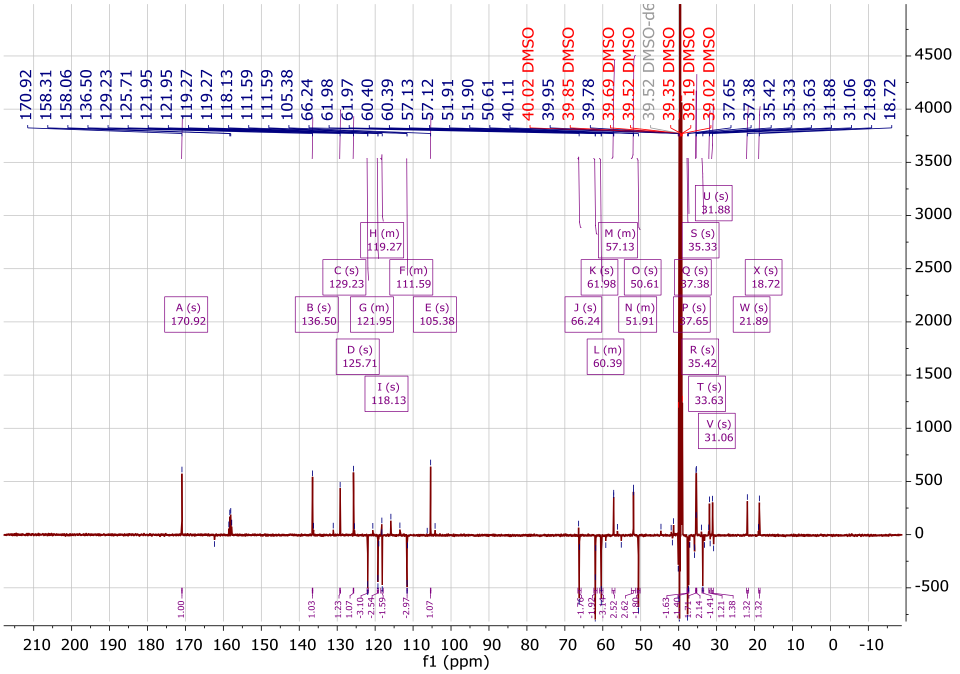

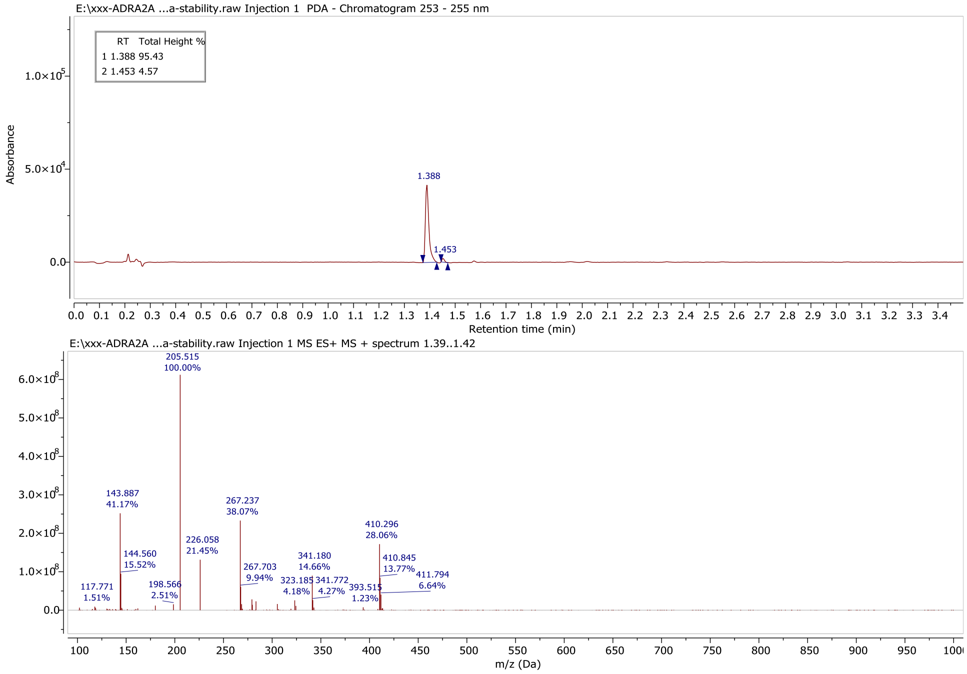

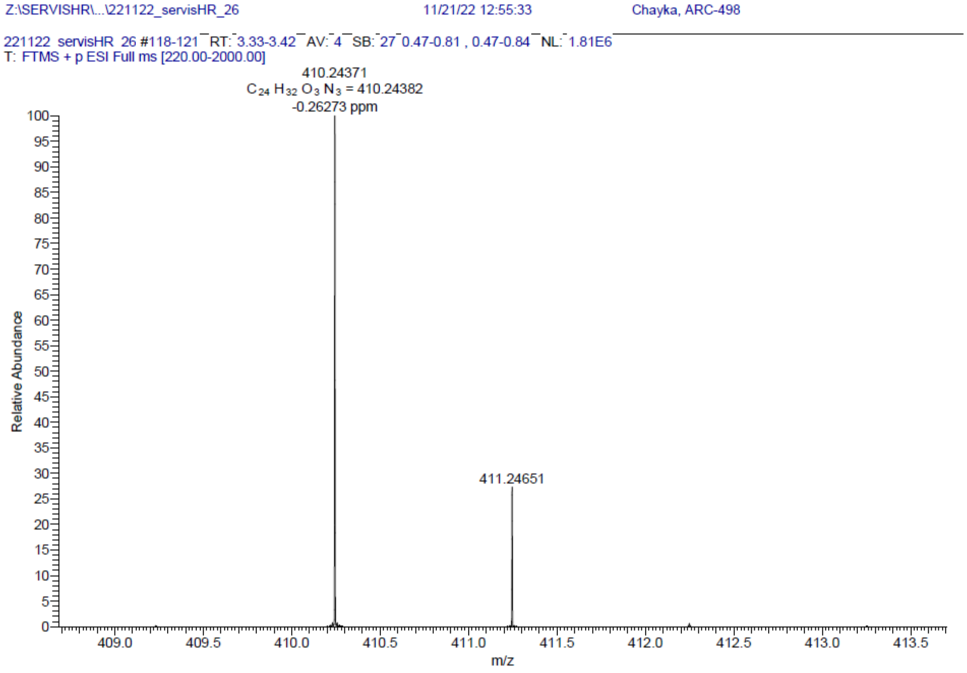

HRMS (ESI+): *m/z* [M + H]$^+$ calculated for $C_{24}H_{32}O_3N_3$ = 410.2438, found: 410.2437.

17-α-Hydroxyyohimban-16-α-carboxylic acid (*S*)-*N*-methylpyrrolidin-3-yl ester (4p)

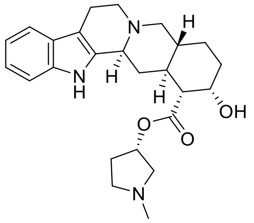

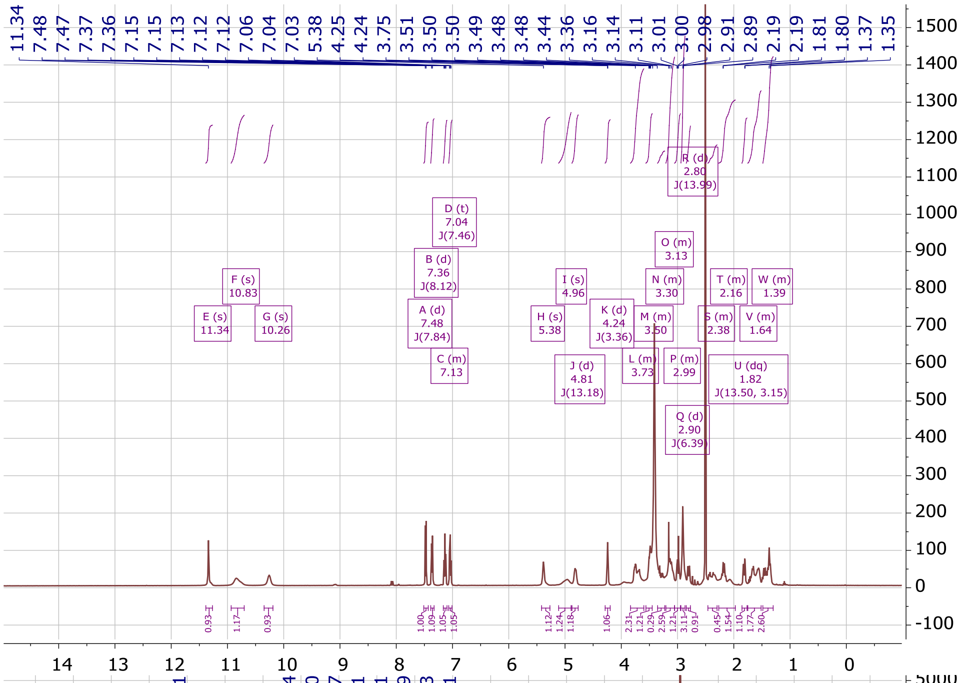

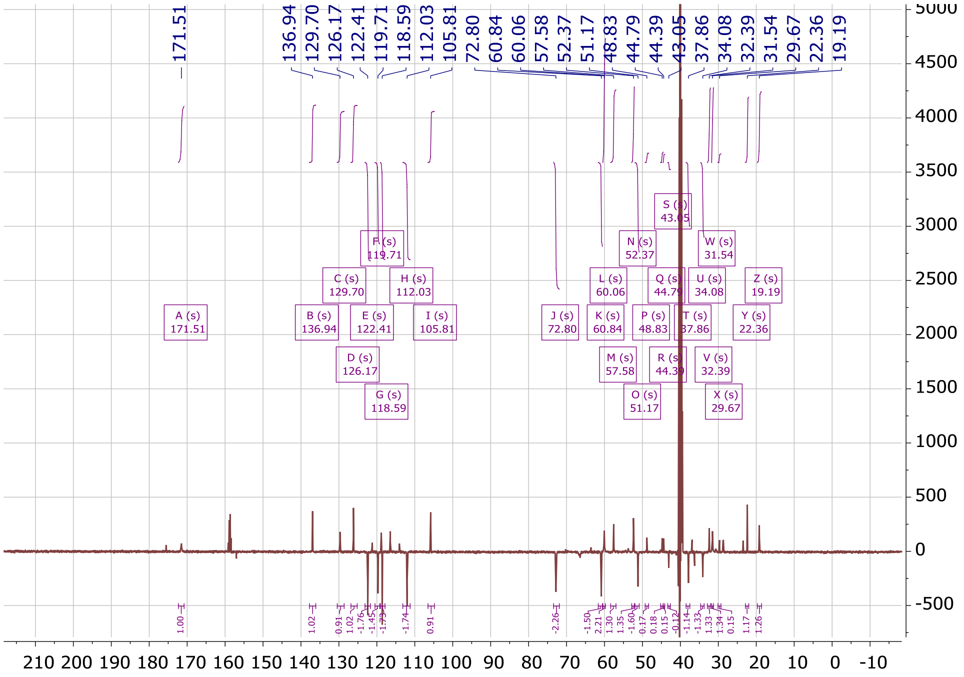

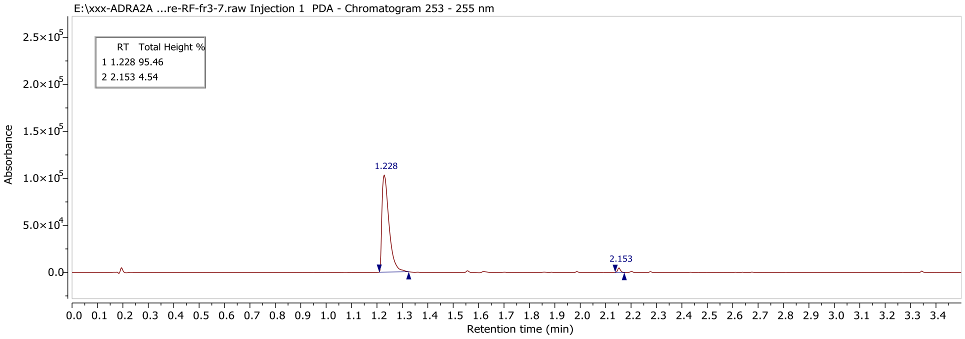

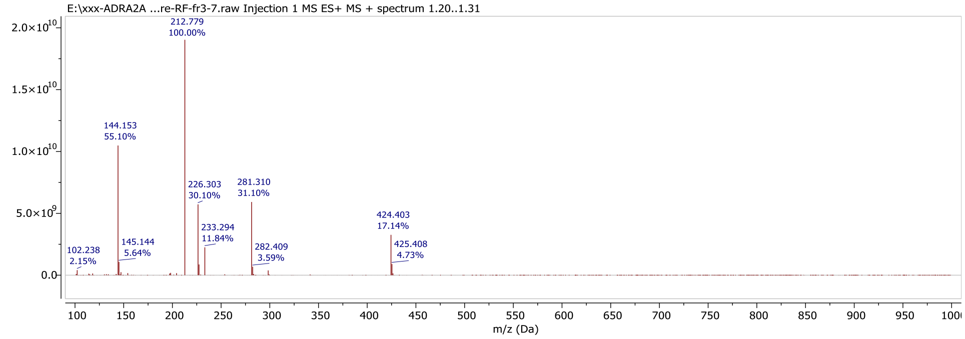

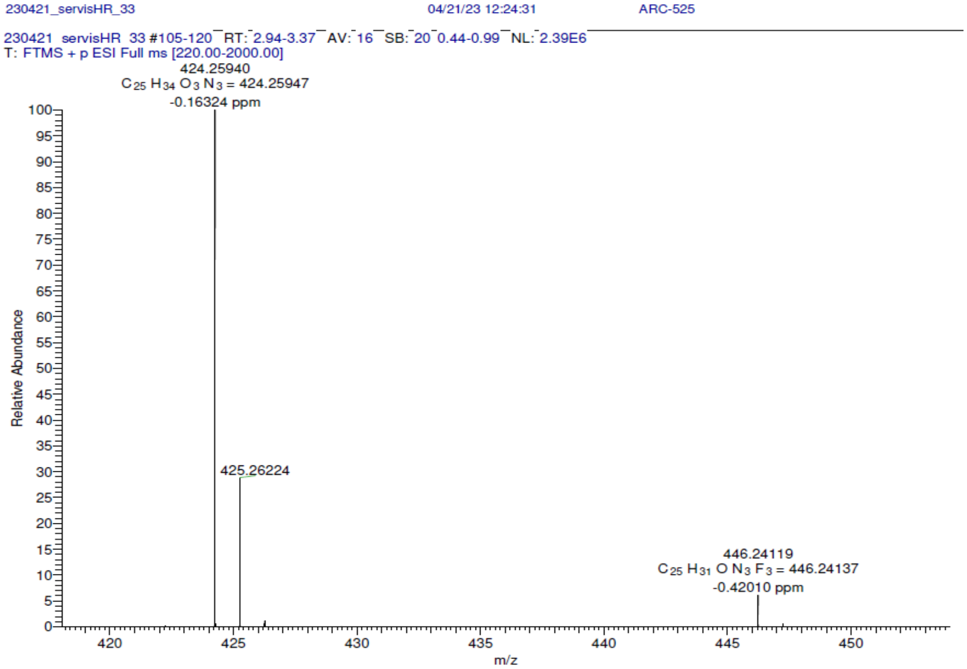

HRMS (ESI+): *m/z* [M + H]$^+$ calculated for $C_{25}H_{34}O_3N_3$ = 424.2595, found: 424.2594.

17-α-Hydroxyyohimban-16-α-carboxylic acid (*S*)-tetrahydrofuran-3-yl ester (4q)

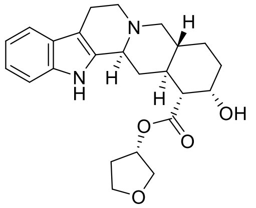

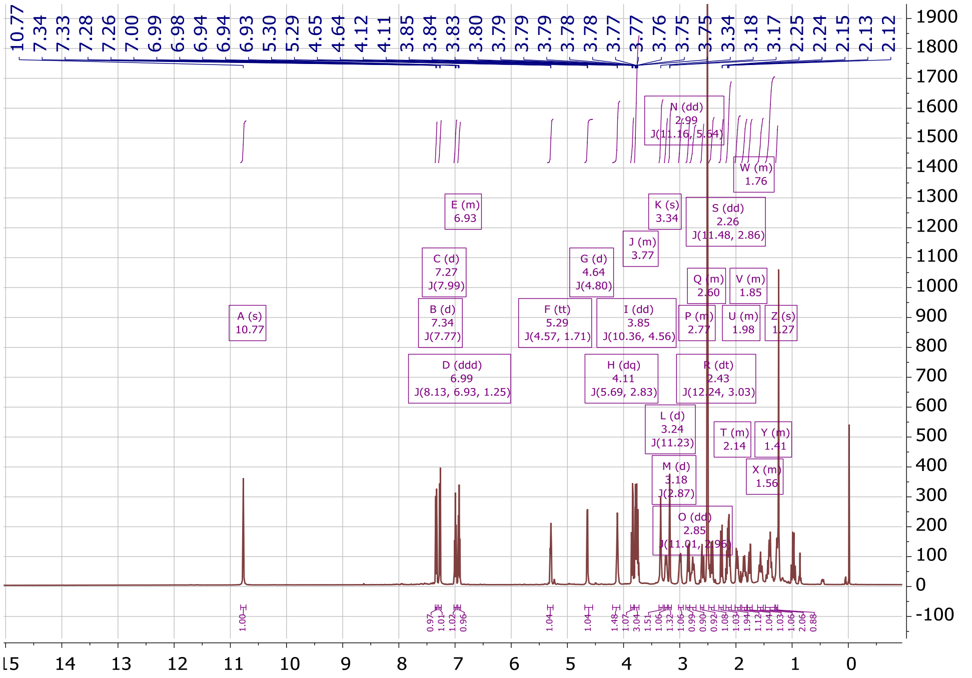

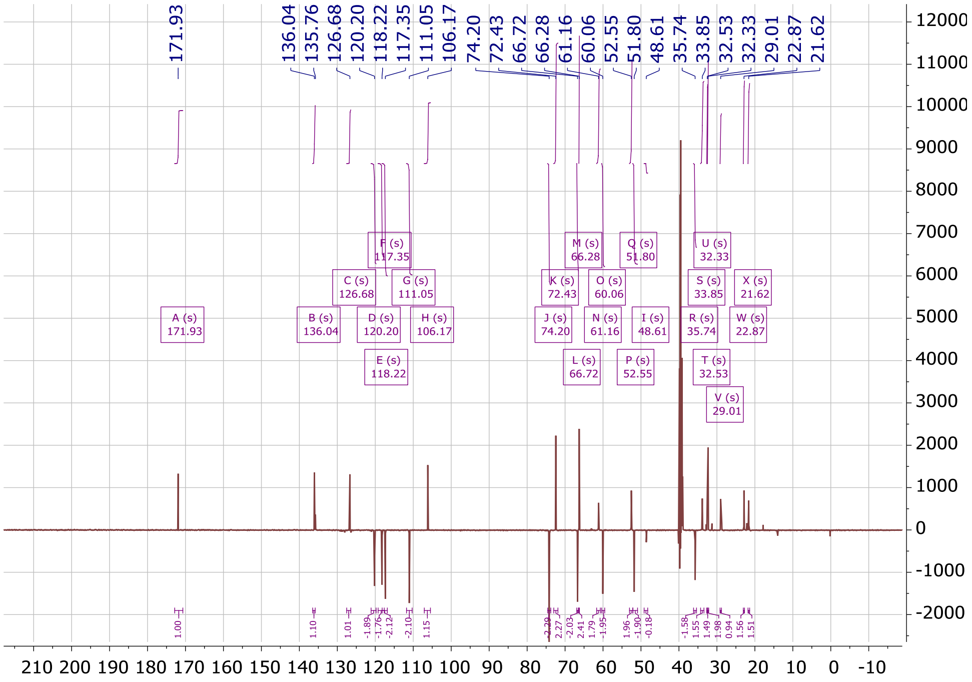

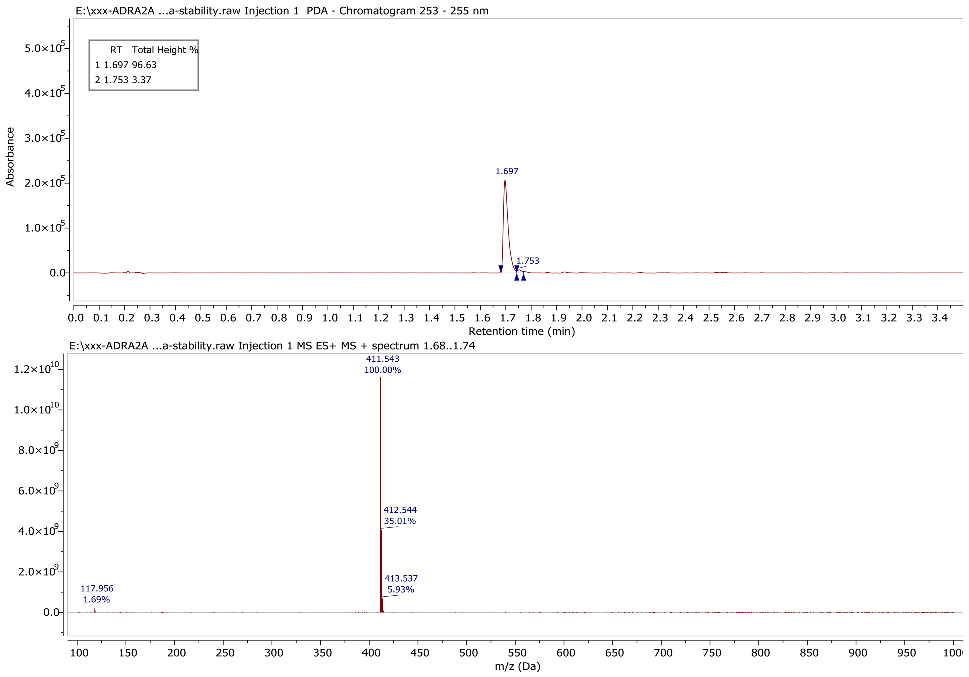

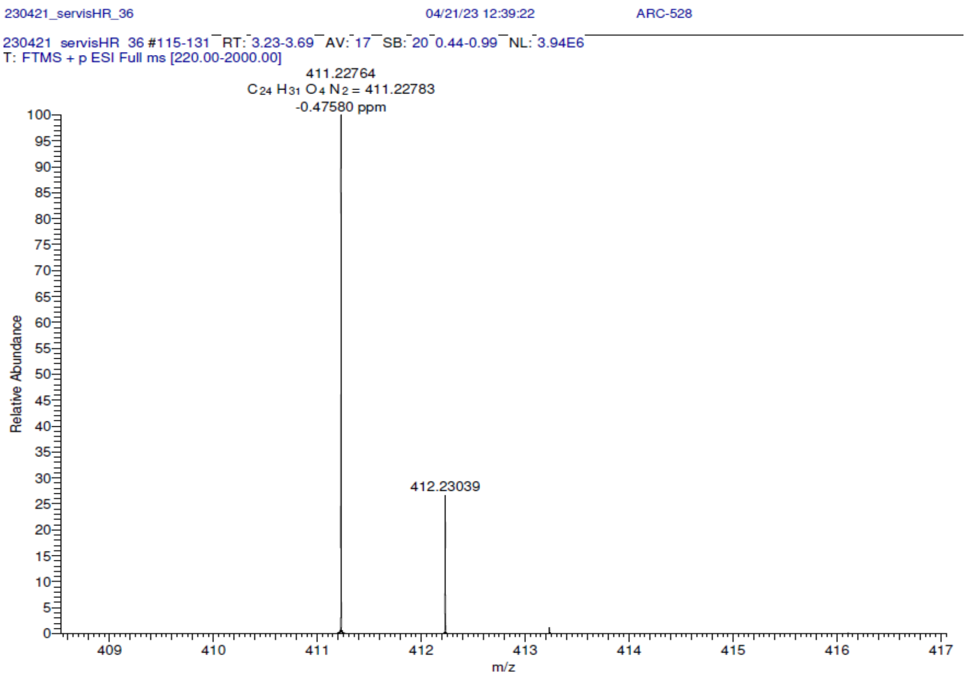

HRMS (ESI+): *m/z* $[M + H]^+$ calculated for $C_{24}H_{31}O_4N_2$ = 411.2278, found: 411.2276.

17-α-Hydroxyyohimban-16-α-carboxylic acid oxetan-3-yl ester (4r)

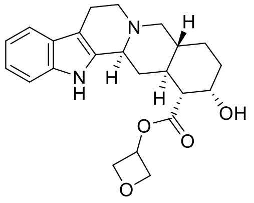

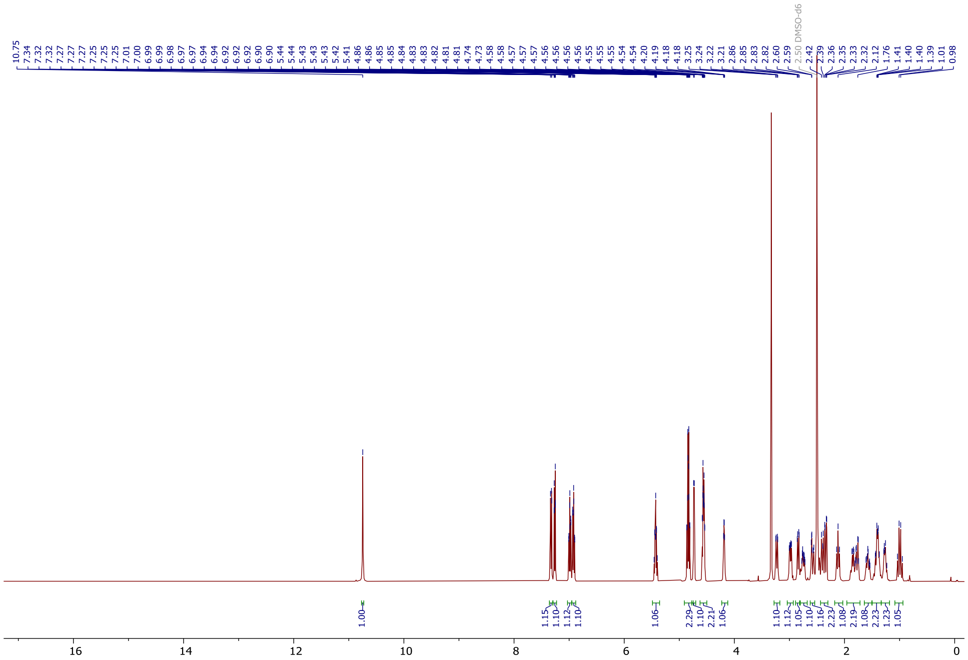

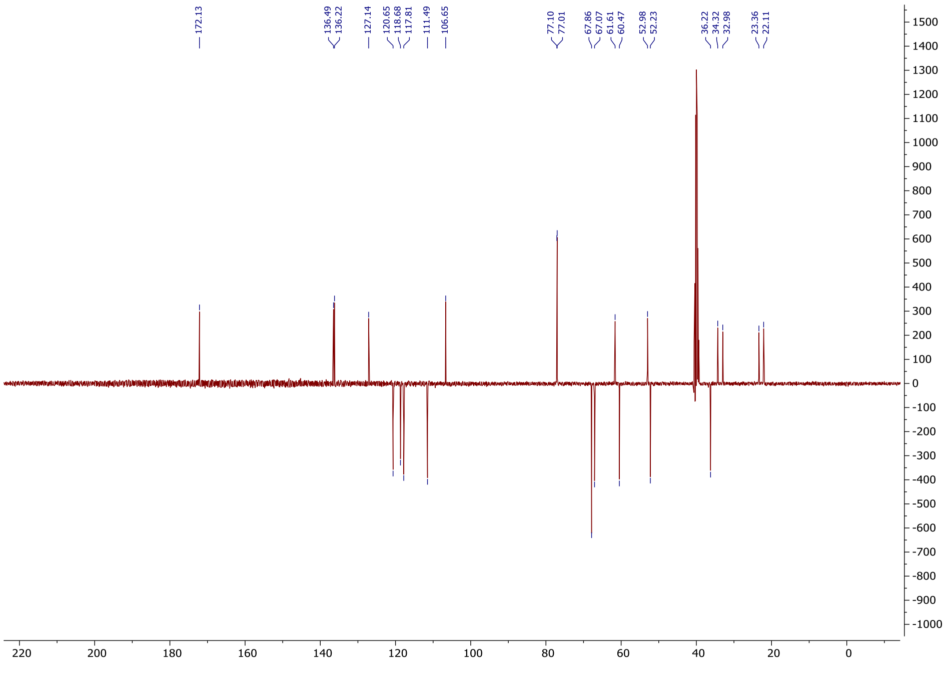

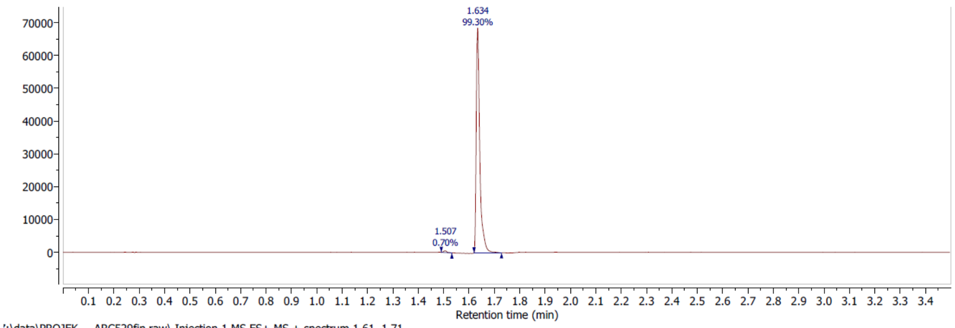

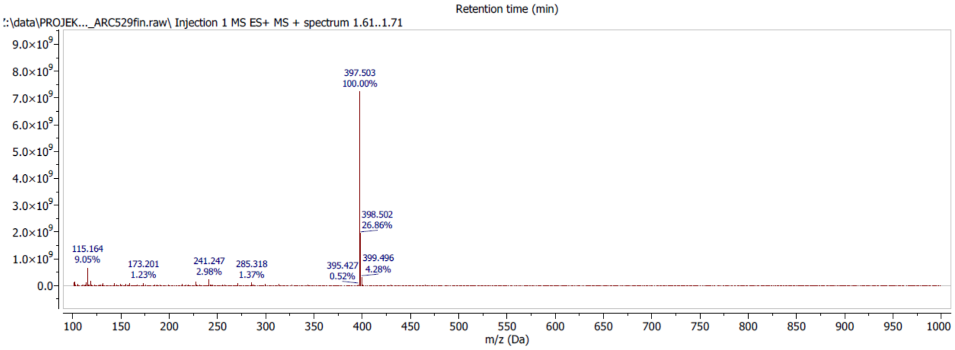

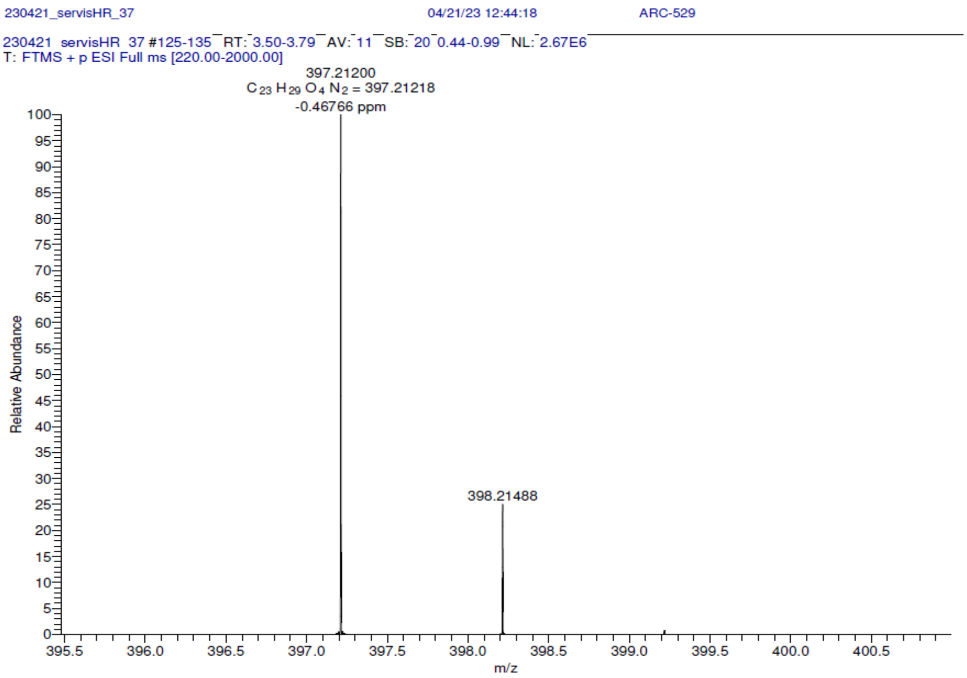

HRMS (ESI+): *m/z* $[M + H]^+$ calculated for $C_{23}H_{29}O_4N_2$ = 397.2122, found: 397.2120.

17-α-Hydroxyyohimban-16-α-carboxylic acid *(S)-N*-(pyrrolidin-3-yl)amide (5a)

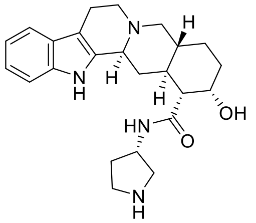

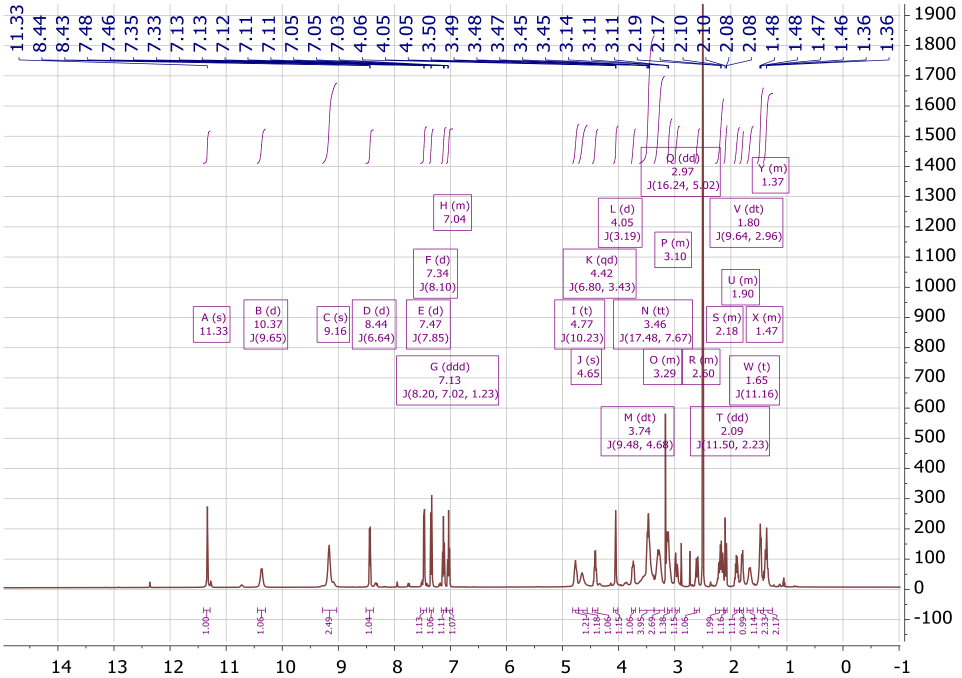

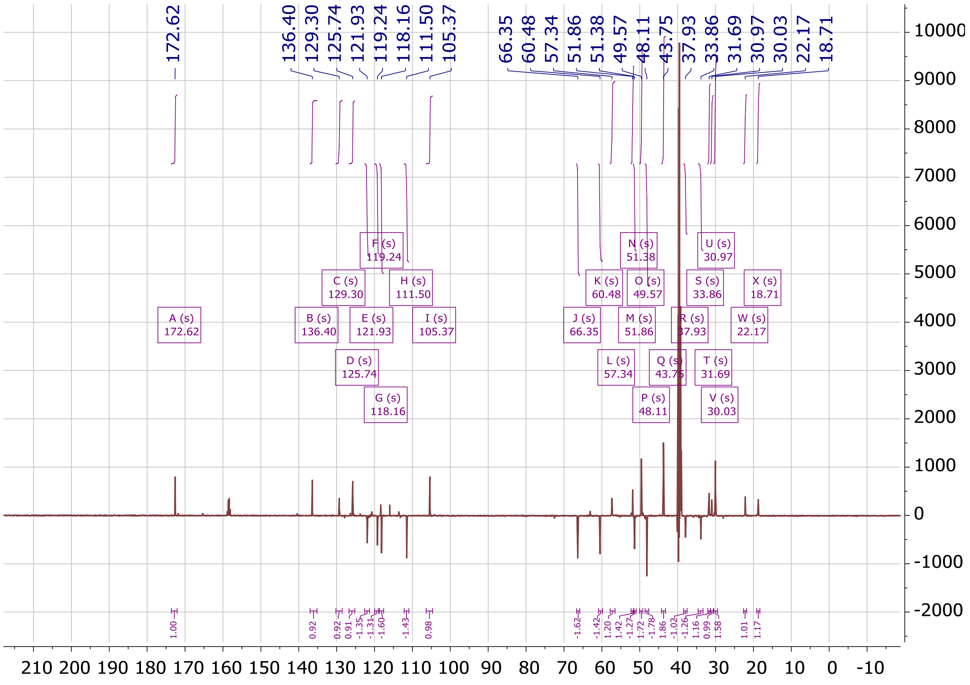

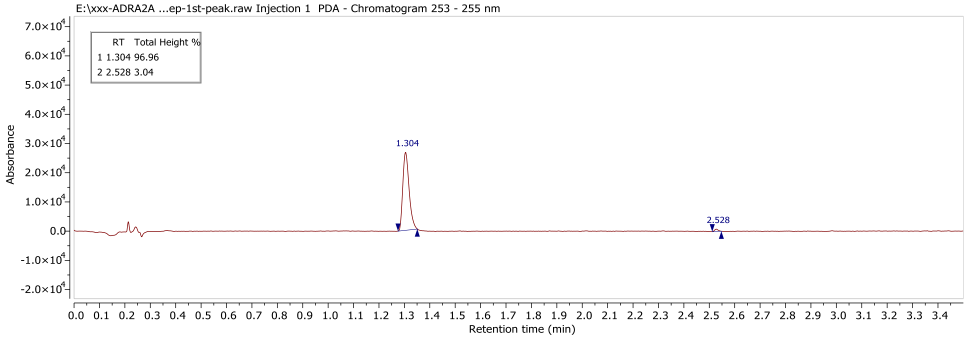

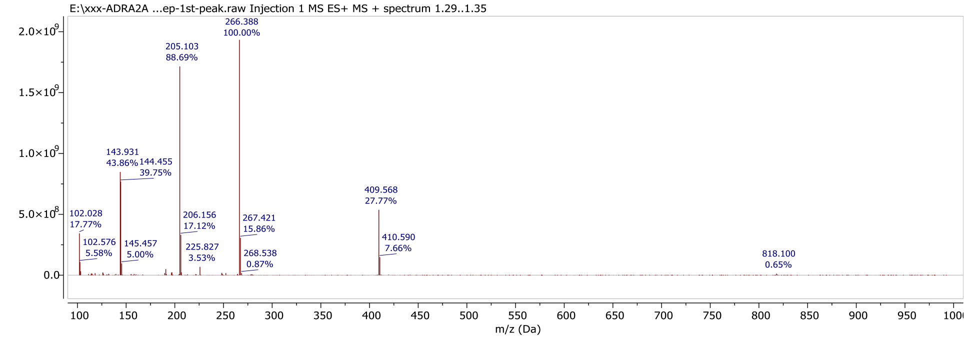

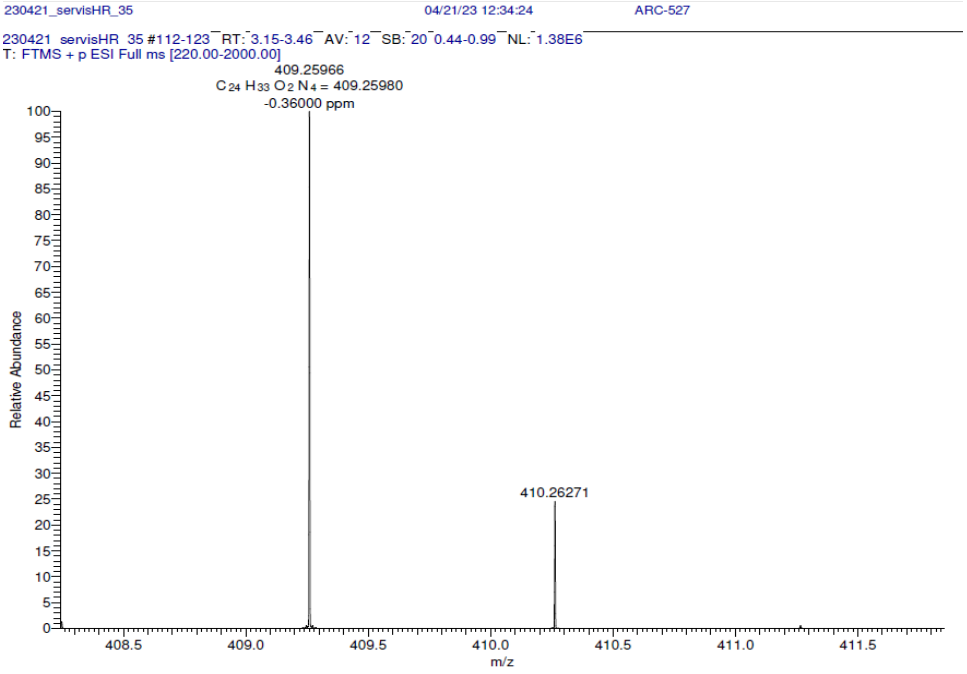

HRMS (ESI+): *m/z* $[M + H]^+$ calculated for $C_{24}H_{33}O_2N_4$ = 409.2598, found: 409.2597.

17-α-Hydroxyyohimban-16-α-carboxylic acid *N*-(azetidin-3-yl)amide (5b)

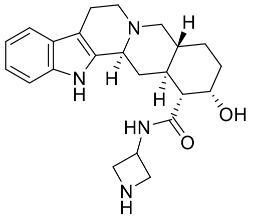

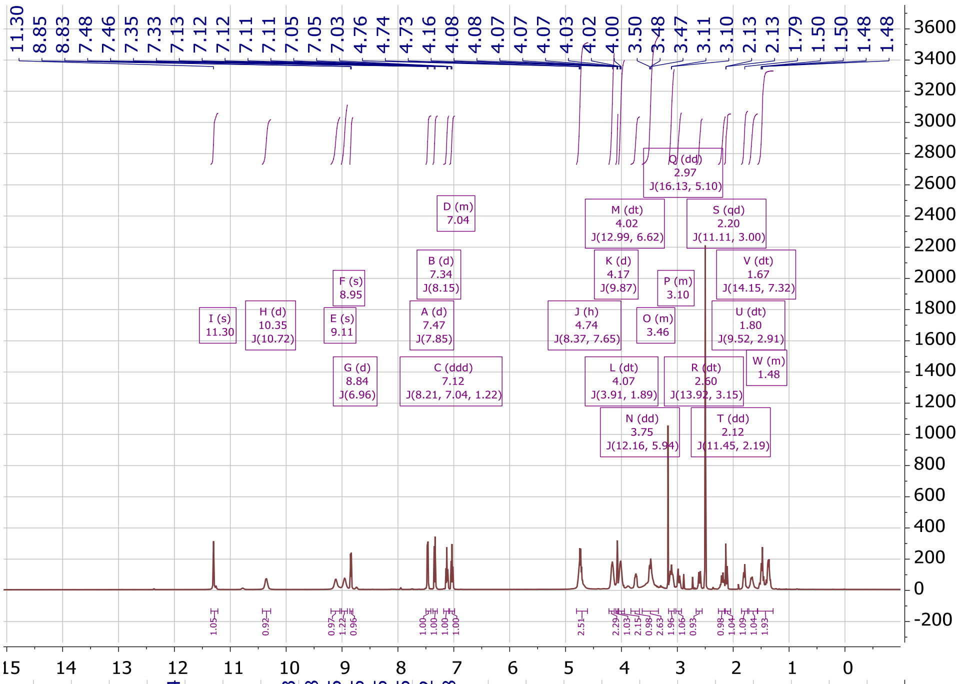

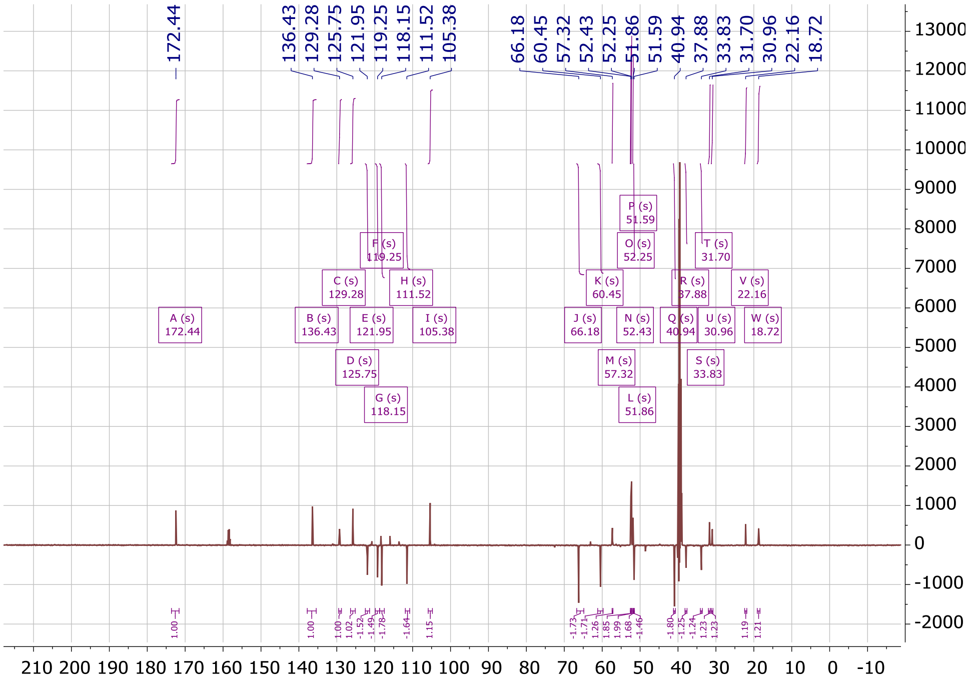

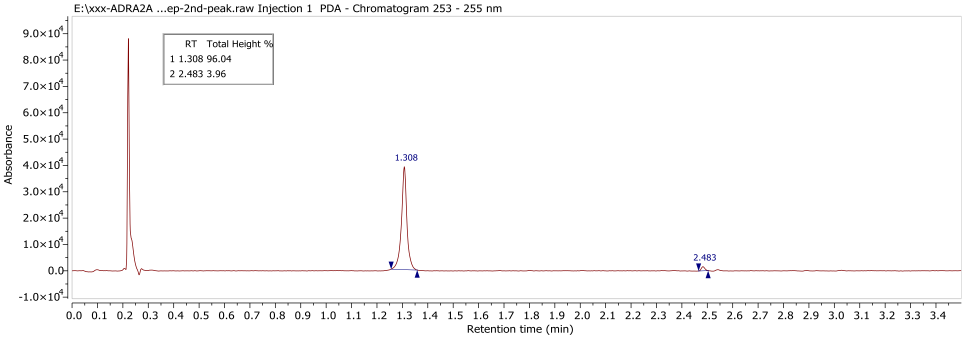

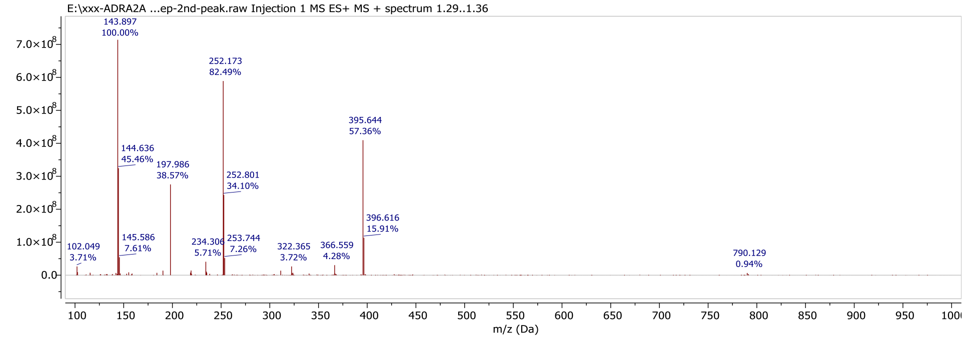

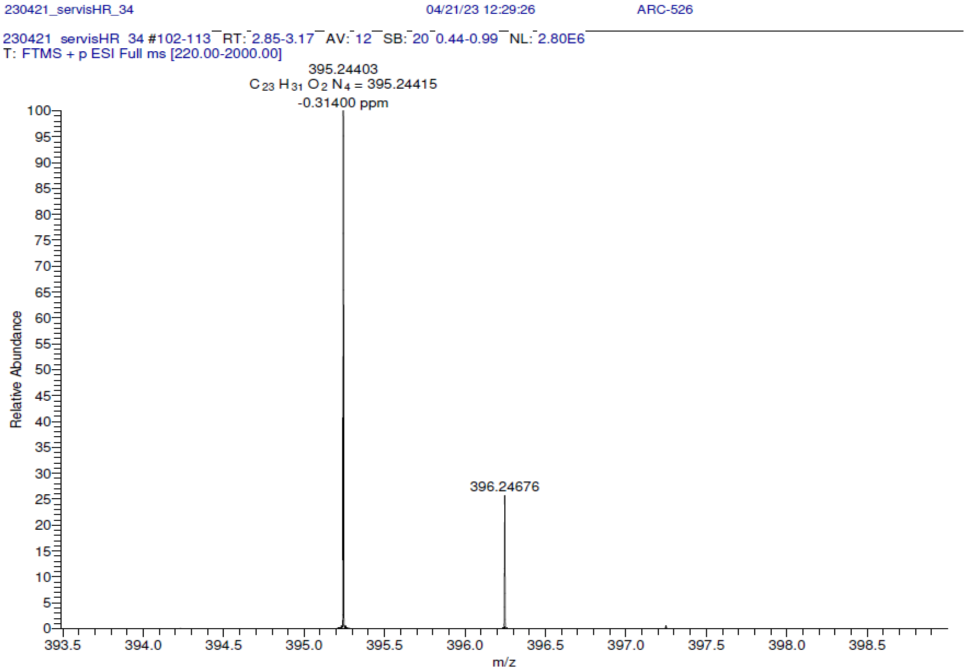

HRMS (ESI+): *m/z* [M + H]$^+$ calculated for $C_{23}H_{31}O_2N_4$ = 395.2442, found: 395.2440.
